# Somatostatin-evoked Aβ catabolism in the brain: Mechanistic involvement of α-endosulfine-K_ATP_ channel pathway

**DOI:** 10.1038/s41380-021-01368-8

**Published:** 2021-11-04

**Authors:** Naoto Watamura, Naomasa Kakiya, Per Nilsson, Satoshi Tsubuki, Naoko Kamano, Mika Takahashi, Shoko Hashimoto, Hiroki Sasaguri, Takashi Saito, Takaomi C. Saido

**Affiliations:** 1 Laboratory for Proteolytic Neuroscience, RIKEN Center for Brain Science, 2-1 Hirosawa, Wako, Saitama, 351-0198 Japan; 2 Karolinska Institutet, Center for Alzheimer Research, Dept. of Neurobiology, Care Science and Society, Division for Neurogeriatrics, Visionsgatan 4, Solna, 171-64 Sweden; 3 Department of Neurocognitive Science, Institute of Brain Science, Nagoya City University Graduate School of Medical Sciences, Nagoya, Aichi 467-8601 Japan; 4 Department of Neuroscience and Pathobiology, Research Institute of Environmental Medicine, Nagoya University, Nagoya, Aichi 464-8601 Japan

**Keywords:** Neuroscience, Molecular biology, Diseases

## Abstract

Alzheimer’s disease (AD) is characterized by the deposition of amyloid β peptide (Aβ) in the brain. The neuropeptide somatostatin (SST) regulates Aβ catabolism by enhancing neprilysin (NEP)-catalyzed proteolytic degradation. However, the mechanism by which SST regulates NEP activity remains unclear. Here, we identified α-endosulfine (ENSA), an endogenous ligand of the ATP-sensitive potassium (K_ATP_) channel, as a negative regulator of NEP downstream of SST signaling. The expression of ENSA is significantly increased in AD mouse models and in patients with AD. In addition, NEP directly contributes to the degradation of ENSA, suggesting a substrate-dependent feedback loop regulating NEP activity. We also discovered the specific K_ATP_ channel subtype that modulates NEP activity, resulting in the Aβ levels altered in the brain. Pharmacological intervention targeting the particular K_ATP_ channel attenuated Aβ deposition, with impaired memory function rescued via the NEP activation in our AD mouse model. Our findings provide a mechanism explaining the molecular link between K_ATP_ channel and NEP activation, and give new insights into alternative strategies to prevent AD.

## Introduction

Alzheimer’s disease (AD) is a progressive neurodegenerative disease characterized by the deposition of amyloid β peptide (Aβ). The identification of pathogenic mutations in the *APP, PSEN1*, and *PSEN2* genes supports the amyloid cascade hypothesis underlying the etiology of AD [1], and verify that these mutations cause early-onset AD due to the abnormal production and accumulation of toxic Aβ species such as Aβ_42_ and Aβ_43_ [2, 3]. In contrast, the exact causes of Aβ deposition in sporadic AD cases remain unclear, although some genetic risk factors related to Aβ metabolism have been identified [4].

Previously, we identified Neprilysin (NEP; neutral endopeptidase 24.11) as a major physiological Aβ-degrading enzyme in brain [5, 6], the expression and activity of which in brain decline with aging and in early stages of AD progression [7–10]. Deficiency of NEP (*Mme*) gene induced ~ twofold Aβ levels in the brain of mice [6]. Therefore, identification of the mechanism(s) that regulate NEP expression and/or activity may contribute to develop ways to prevent AD. Indeed, gene therapeutic approaches in mice using adeno-associated virus carrying *MME* gene reduced amyloid deposition and alleviated abnormal memory function [11, 12]. A meta-analysis of genome-wide association studies identified a variant in the *MME* gene, which leads to a change in the amino acid sequence, as a risk factor for AD, implying the potential significance of NEP in the etiological processes underlying AD development [13].

We previously showed that somatostatin (SST), a neuropeptide known as a somatotropin-release inhibiting hormone [14], regulates Aβ_42_ levels in the brain via the upregulation of NEP [15]. SST mRNA levels were significantly decreased with aging, particularly in the postmortem brain with AD [16–21], suggesting that the aging-induced downregulatiuon of SST expression may be a trigger for the Aβ pathology in late-onset AD. In addition, we discovered that, of the five SST receptor (SSTR1–5) subtypes, SSTR1 and SSTR4 redundantly regulate NEP activity and modulate Aβ_42_ levels in the brain [22, 23]. However, the mechanism by which SST signaling regulates NEP activity remains unclear. In the present study, we address how NEP activity is regulated in the signaling cascade downstream of SST.

## Materials and Methods

### Animals

All animal experiments were conducted according to guidelines of the RIKEN Center for Brain Science. *Srif* KO and *Sst*_*4*_ KO mice were kindly provided by Oklahoma Medical Research Foundation as described previously [22]. *Sst*_*1*_ KO mice were purchased from Jackson laboratory. *Sst*_*1*_*/Sst*_*4*_ dKO mice were generated as described previously [22]. *Mme* KO mice were used as negative controls [24]. *Abcc8* KO mice were generated as described previously [25], and kindly provided by Department of Phamacology, Tübingen University. *Kncj8* KO and *Kncj11* KO mice were generated as described previously [26, 27], and kind gift from Center for Animal Resources and Development, Kumamoto University and RIKEN BioResource Research Center. C57BL/6 J and ICR mice were used as zygote donors and foster mothers. C57BL/6 J mice were also used for backcrossing with *Ensa* KO mice. *App*^*NL-F*^ mice harbor the humanized sequence of Aβ, and the Swedish (KM670/671NL) and Iberian (I716F) mutations, while *App*^*NL-G-F*^ mice harbor the Arctic (E693G) mutation in addition to the humanized sequence of Aβ, and Swedish (KM670/671NL) and Iberian (I716F) mutations as previously described [28]. Male mice were used in all experiments.

### Antibodies

Antibodies used in this research are listed in Supplementary Table S1. The specificity of ENSA antibody was confirmed using the *Ensa* KO mouse.

### Primary neurons

Neurons from the cerebral cortex, hippocampus and basal ganglia regions of brains from embryonic day (E) 16–18 C57BL/6Ncr mice were isolated and cultured. Briefly, brains were excised and placed in culture plates (FALCON) containing neurobasal medium (Thermo Fisher Scientific). The aforementioned brain regions were excised by scalpel and treated with 5 ml of 0.25% trypsin solution (Nacalai tesque 32777-44) at 37 ˚C for 15 min. 250 µl of 1% DNase I was added by pipette and mixed. Subsequently, centrifugation was performed at 1500 rpm for 5 min and 5 ml of Hank’s buffered salt solution containing 250 µl of 1% DNase I was added to the pellet and incubated in a water bath at 37 ˚C for 5 min. An additional 10 ml of Hank’s buffered salt solution was added to the mixture and centrifuged at 1500 rpm for a further 5 min. The resulting pellet was added to neurobasal medium with B27 Plus Supplement (Thermo Fisher Scientific 17504044) and 25 µM glutamine (Thermo Fisher Scientific 05030-149). The cells were filtered using a cell strainer with 100 µm nylon mesh (Falcon 2360), and seeded on 6- or 96-well plates (Falcon 353046 or Corning 356640). Cortical/hippocampal and basal ganglia neurons were mixed in a 9:1 ratio as co-cultured neuron.

### Neprilysin activity

NEP activity measurements were performed on primary neurons after 15–28 days of in vitro (DIV15–28) culture as previously described [29]. Somatostatin (Peptide institute 4023), TT232 (Tocris 3493), recombinant ENSA (abcam ab92932), recombinant NSG-1 (Creative BioMart NSG1–332H), recombinant NUCKS-1 (Creative BioMart NUCKS1–10956M) and diazoxide (Wako 364-98-7) were added as appropriate concentrations, and cells were incubated for a further 24 h. Neurons were then incubated with substrate mixture 50 µM suc-Ala-Ala-Phe-MCA (Sigma S8758), 10 nM benzyloxycarbonyl Z-Leu-Leu-Leucinal (Peptide institute 3175-V) and cOmplete EDTA-Free-Protease inhibitor (Roche Diagnostics 4693132) in 0.2 M MES buffer (pH6.5) with or without Thiorphan (Sigma T6031) for 1 h at 37 ˚C. Following this, 0.1 mM phosphoramidon (Peptide Institute 4082) and 0.1 mg/ml leucine aminopeptidase (Sigma L-5006) were added, and the reaction mixture was incubated at 37 ˚C for a further 30 min. 7-Amino-4-methylcoumarin fluorescence was measured at excitation and emission wavelengths of 380 nm and 460 nm, respectively. Centrifugal 10 and 30 kDa filters (Merck UFC503096, 501096) were used to separate conditioned media obtained from cortical/hippocampal neurons.

### Preparation of membrane fractions from brain tissue

Brain tissues were homogenized in Tris-buffer (50 mM Tris pH 8, 0.25 M sucrose, EDTA-free cOmplete protease inhibitor cocktail (Roche Diagnostics 05056489001)) and centrifuged at 3600 rpm and 4 ˚C for 30 min. Collected supernatants were centrifuged at 70,000 rpm at 4 ˚C for 20 min. Resultant pellets were solubilized in Tris-buffer containing 1% Triton X-100 and incubated on ice for 1 h before centrifugation at 70,000 rpm at 4 ˚C for 20 min. Protein concentrations of membrane fractions in collected supernatant samples were measured by BCA protein assay kit (Thermo Fisher Scientific 23225).

### LC-MS/MS analysis

50 mM Ammonium bicarbonate, 10% acetonitrile and 20 mM dithiothereitol were added to the conditioned media and incubated for 30 min at 56 ˚C. Samples were then treated with 30 mM iodoacetamide and incubated for 30 min at 37 ˚C and digested by incubation with 100 ng/µl trypsin overnight at 37 ˚C. Peptide sequences were determined by Q Exactive Orbital Mass Spectrometers (Thermo Fisher Scientific) [30]. We used Proteome Discoverer Software (Thermo Fishier Scientific) for identification of proteins and peptides. Proteins identified in conditioned media are listed in Supplementary Tables S2 and 3.

### Preparation for Cas9 and sgRNAs

For synthesis of Cas9 mRNA in vitro, plasmid vector pCAG-T3-hCAS-pA (Addgene 48625) was linearized by Sph I, then transcribed with T3 RNA polymerase (Promega) in the presence of Ribo m^7^G Cap Analog (promega) as previously described [31]. The MEGAshortscript T7 (Thermo Fisher Scientific AM1354) and MEGAclear (Thermo Fisher Scientific AM1908) kits were used for in vitro transcription of sgRNAs, while the CRISPR Design tool was used for creating sgRNAs [32]. All oligonucleotide sequences used for in vitro transcription are listed in Supplementary Table S4.

### Microinjection of mouse zygotes

The SpCas9 mRNA (60 ng/µl) and sgRNAs (30 ng/µl) were injected into the cytoplasm of C57BL/6 J zygotes. After incubation at 37 ˚C for 24 h, embryos developed to the 2-cell-stage were transplanted into host ICR mice.

### Off-target analysis

Off-target sites that accepted up to three mismatches were determined by COSMID (https://crispr.bme.gatech.edu/) [33], listed in Supplementary Table S5. Target sites were amplified from tail genomic DNA by PCR using the Ex Taq Polymerase kit (Takara RR001A) with primers listed in Supplementary Table S6. Target sequencing was performed using a DNA sequencer (ABI 3730xl).

### Genotyping

Genomic DNA was extracted from mouse tail using lysis buffer (100 mM Tris pH 8.5, 5 mM EDTA, 0.2% SDS, 200 mM NaCl, 20 µg/µl Proteinase K) and PCR performed using the specific primer set listed in Supplementary Table S7. PCR products were analyzed by MultiNa (Shimadzu) to evaluate the efficiency of the CRISPR-mediated deletion of the *Ensa* gene. Sanger sequencing analyses were conducted using a DNA sequencer (ABI 3730xl).

### Western blot analysis

Mouse brains were homogenized with lysis buffer (50 mM Tris pH 7.6, 0.15 M NaCl and cOmplete protease inhibitor cocktail (Roche Diagnostics 11697498001)) using a Multi-bead shocker MB (Yasui-Kikai). Samples were rotated at 4 ˚C for 1 h and centrifuged at 15000 rpm for 30 min. Supernatants were collected as lysates and then subjected to sodium dodecyl sulfate-polyacrylamide gel electrophoresis (SDS-PAGE) and transferred to a PVDF or nitrocellulose membranes. For detection of ENSA and CTF-APP, membranes were boiled in PBS for 5 min, treated with ECL prime blocking buffer (GE healthcare RPN418) for 1 h and incubated with antibody at 4 ˚C. Dilution ratios of antibodies are listed in Supplementary Table S1. Immunoreactive bands were visualized by ECL Select (GE Healthcare RPN2235) and a LAS-3000 Mini Lumino image analyzer (Fujifilm).

### Co-incubation of ENSA and NEP

In total, 25 ng/µl of ENSA were co-incubated with 2.5 ng/µl NEP, 0–500 nM Aβ_42_ (Peptide Institute 4420-s) and arctic Aβ_42_ (Peptide Institute AF-721), 0.1 mM thiorphan, 1 mM phosphoramidon, and 10 mM EDTA at 37 ˚C for 24 h in 0.2 M MES buffer pH 6.5.

### Immunohistochemical analysis

After deparaffinization of paraffin-embedded mouse brain sections, antigen retrieval was performed by autoclaving at 121 ˚C for 5 min. Sections were then treated with 0.3% H_2_O_2_ in methanol to inactivate endogenous peroxidases. Next, sections were rinsed several times with TNT buffer (0.1 M Tris pH 7.5, 0.15 M NaCl, 0.05% Tween20), blocked for 30 min (TSA Biotin System kit), and incubated overnight at 4 ˚C with primary antibody diluted in TNB buffer (0.1 M Tris pH 7.5, 0.15 M NaCl). Sections were rinsed several times and incubated for 1 h at room temperature with biotinylated secondary antibody (Vector Laboratories). Next, sections were incubated with HRP-conjugated avidin for 30 min and tyramide-enhanced FITC or rhodamine for 10 min. Finally, sections were treated with DAPI (Cell Signaling Technology 4083 S) diluted in TNB buffer before mounting with PermaFluor (Thermo Fisher Scientific TA-030-FM). Sections were scanned on a confocal laser scanning microscope FV-1000 (Olympus) and a NanoZoomer Digital Pathology C9600 (Hamamatsu Photonics) followed by quantification with Metamorph Imaging Software (Molecular Devices) and Definiens Tissue Studio (Definiens). Quantitative methods for colocalized signals of NEP and VGAT have been described previously [15].

### Enzyme-linked immunosorbent assay

Mouse cortices were homogenized in TBS buffer (50 mM Tris pH 7.6, 150 mM NaCl, protease inhibitor cocktail) by a Multi-bead shocker (YASUI KIKAI), centrifuged at 70,000 rpm for 20 min, and supernatants collected as Tris-soluble fractions. Pellets were rinsed with TBS buffer following which 6 M guanidine-HCl solution was added and mixed with a Pellet Pestle (KIMBLE). The mixture was then incubated for 1 h at room temperature. Next, samples were centrifuged at 70,000 rpm for 20 min and supernatants collected as guanidine-soluble fractions. Tris-soluble fractions and guanidine-soluble fractions were applied to 96-well plates. Aβ_40_ and Aβ_42_ levels were measured with the aid of an Aβ-ELISA kit (Wako 294–62501,294–62601).

### Determination of amino acid sequence of NEP-cleaved ENSA

After co-incubation of ENSA and NEP with or without thiorphan, MALDI-TOF analysis was performed using Autoflex speed (BRUKER) to detect the specific fragment of ENSA cleaved by NEP. LC-MS/MS analysis was then performed to determine the specific amino acid sequences. Data from LC-MS/MS analyses are listed in Supplementary Table S8.

### SFV injection

WT mice at 3 months were used for this experiment. SFV-NEP vectors (active and inactive forms) were developed previously [34]. Mice were anesthetized with a triple mixed anesthetic (Domitor 0.3 mg/Kg, Dormicum 4 mg/kg, Bettlefar 5 mg/kg), with SFV then injected into the bilateral hippocampus (stereotaxic coordinates: anteroposterior, −2.6 mm; mediolateral, ±3.1 mm; dorsoventral, −2.4 mm) in a total volume of 1 μl using a Hamilton syringe (Altair Corporation), at a constant flow rate of 0.1 μl/min using a Legato 130 syringe pump (KD Scientific, Hollistoon, MA). After injection, mice were administrated with Antisedan 3 mg/kg and maintained for 48 h in cages with free access to food and water.

### K_ATP_ channel human studies

GSE15222, 95587, and 125583 microarray and RNA-Seq datasets were downloaded from the Gene Expression Omunibus (GEO, https://www.ncbi.nlm.nih.gov/geo/) to compare the mRNA levels of each K_ATP_ channel component in samples from pathophysiologically diagnosed AD and control tissue. These data were published previously [35–38], but are publicly available from GEO. Statistical data summaries were downloaded from Srinivasan et al. that allowed us to appreciate the considerable differences in gene expression existing between healthy control and AD subjects in each dataset [36]. For additional information about the sample processing and quality control steps, please see [35–38].

### Diazoxide treatment

Diazoxide was diluted in drinking water and administered to WT and *App*^*NL-F*^ mice (10 mg/kg/day). For the short-term treatment, diazoxide was administrated to 3-month-old WT mice for 1 month, while in the long-term treatment, diazoxide was administrated to 15-month-old WT and *App*^*NL-F*^ mice for 3 months. After diazoxide treatment for 3 months, mice were subjected to behavioral tests followed by brain dissection.

### Open field test

WT mice and *App*^*NL-F*^ mice were placed individually in a white noise box for at least 1 h before starting the test. They were then placed in the middle of an open field maze (600 × 600 mm) and allowed to explore in the area for 10 min. The amount of time that mice spent in the central region was measured as an anxiety parameter.

### Contextual fear conditioning test

Before the start of test, the mice were put in the white noise box for at least 1 h. Subsequently, the mice were placed into a sound-attenuating chamber and allowed to explore the chamber for 5 min. The percent freezing time was measured until mice received an electric shock (7.5 mA) to the foot after 4 min. As a long-term retention test, the same conditioning experiments were repeated daily for 4 days. The training box was cleaned with water and wiped dry with paper toweling before the next mouse was placed in the chamber. Mice were returned to their cages and provided with free access to food and water.

### Human tissues

Brain samples were kindly provided by Dr. John Trojanowski (University of Pennsylvania) in compliance with RIKEN ethics committee guidelines (approval number Wako3 30-4(2)). Other human samples were obtained from Bio Chain and Tissue solutions. All samples are listed in Supplementary Table S9.

### Statistics

All data are shown as the mean ± SEM. For comparisons between two groups, data were analyzed by Student’s-, Welch’s- *t* test or Mann–Whitney test. For comparisons among more than three groups, we used one-way analysis of variance (ANOVA) followed by Dunnett’s post hoc analysis or Tukey’s post hoc analysis. In the contextual fear conditioning test, we used two-way ANOVA followed by Tukey’s post hoc analysis. All data were analyzed by Prism7 software (San Diego, CA, USA).

## Results

### Identification of ENSA as a regulator of NEP activity in vitro

**Fig. 1:**
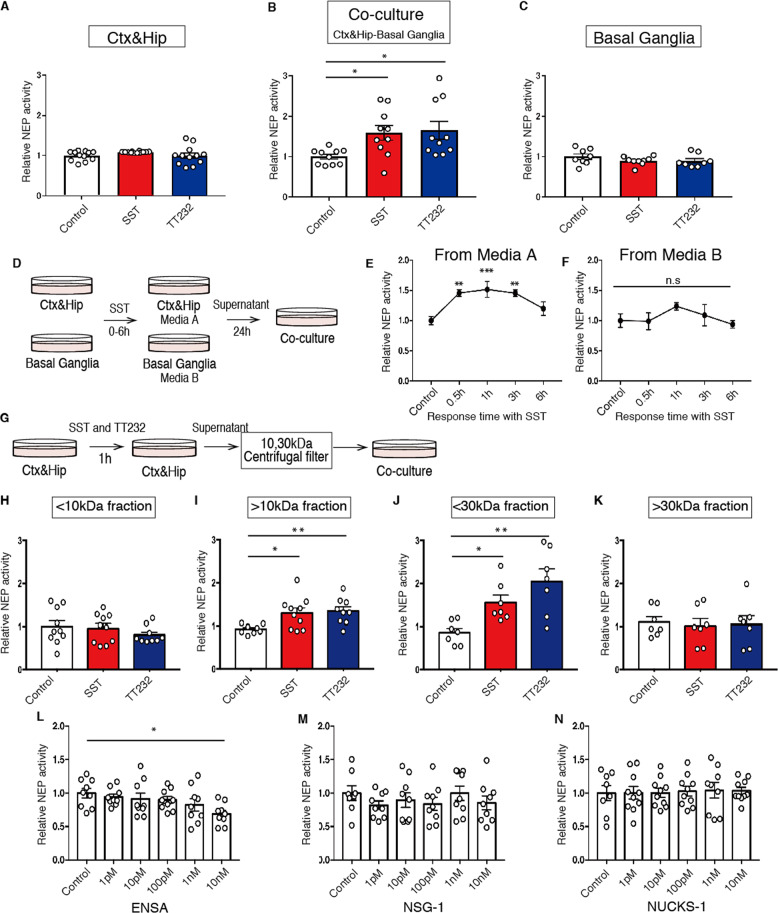
Identification of ENSA as a NEP regulator in vitro. A-C. NEP activity after treatment of co-cultured cells with 1 µM somatostatin or TT232 for 24 h. **A** Cortical/hippocampal (Ctx&Hip) neurons (*n* = 12 wells per treatment), (**B**) co-cultured neurons (*n* = 10 wells per treatment), and (**C**) basal ganglia neurons (*n* = 8 or 9 wells per treatment) were used. **D**–**F** NEP activity in co-cultured neurons after the replacement of the culture medium with conditioned media from (**E**) Ctx&Hip and (**F**) basal ganglia neurons treated with 1 µM somatostatin for 0–6 h. *n* = 6–10 wells per treatment in co-cultured neurons. **G**–**K** NEP activity of co-cultured neurons after replacement of the culture medium with separated conditioned media from Ctx&Hip neurons treated with SST or TT232. **H**, **I** 10 and (**J** and **K**) 30kDa centrifugal filters were used for the separation (*n* = 7–10 for each group). NEP activity in co-cultured neurons after incubation with (**L**) ENSA, (**M**) NSG-1 and (**N**) NUCKS-1 recombinant proteins for 24 h. *n* = 8–10 wells per treatment in co-cultured neurons. Data represent the mean ± SEM. *P < 0.05, **P < 0.01, ***P < 0.001 (one-way ANOVA with Dunnett’s post-hoc test).

We previously developed a method for measuring NEP activity in the co-culture system composed of cortical/hippocampal and basal ganglia neurons, which contain both SSTR1 and SSTR4 [22]. Treatment of co-cultured neurons of E16–18 C57BL/6Ncr mice, but not of neurons from the individual brain regions, with SST or TT232, a selective agonist for SSTR1 and SSTR4, respectively, elevated NEP activity (Fig. 1A–C). Co-cultured neurons derived from SSTR1 and SSTR4 double knockout (*Sst*_*1*_*/Sst*_*4*_ dKO) mice failed to exhibit the SST-induced NEP upregulation (Supplementary Fig. S1A–C). We next examined if neurons from cortical/hippocampal and/or basal ganglia generate a secretory factor that activates NEP in the mixed culture neurons. Cultured cortical/hippocampal and basal ganglia neurons were treated separately with SST, and the collected conditioned media added to co-cultured neurons (Fig. 1D). We collected media from SST-treated WT cortical/hippocampal neurons (Media A) and from basal ganglia neurons (Media B), and found that only Media A significantly elevated the NEP activity of co-cultured neurons (Fig. 1E and F). Media A also elevated NEP activity in co-cultured neurons derived from *Sst*_*1*_*/Sst*_*4*_ dKO mice (Supplementary Fig. S1D and E), indicating that the NEP-stimulating element in question is secreted by cortical/hippocampal neurons but not by basal ganglia neurons, and that NEP activity is upregulated downstream of the SST-SSTR binding.

A centrifugal filter was used to concentrate NEP activity modulators to the 10–30 kDa molecular weight range (Fig. 1G–K), and this fraction was then subjected to LC-MS/MS analysis to identify candidate mediators. Initially, we performed a qualitative comparison between proteins identified in the conditioned media from wild-type primary neurons treated with or without SST and TT232. We also used conditioned media from *Sst*_*1*_*/Sst*_*4*_ dKO mice as a negative control. We then searched for proteins absent or present only in the media of the SST- and TT232-treated WT neurons, but not in the media of *Sst*_*1*_*/Sst*_*4*_ dKO neurons. In this way, we identified three candidate proteins: (1) ENSA, (2) Neuron-specific protein family member 1 (NSG-1) and (3) Nuclear ubiquitous casein and cyclin-dependent substrate 1 (NUCKS-1) (Supplementary Tables S2 and S3). To determine which of the candidates is involved in the regulation of NEP activity, we analyzed the effects of corresponding recombinant proteins on NEP activity in co-cultured neurons. Only the recombinant ENSA decreased NEP activity in co-cultured neurons from WT and *Sst*_*1*_*/Sst*_*4*_ dKO mice in a dose-dependent manner (Fig. 1L–N, and Supplementary Fig. S1F).

We next analyzed ENSA levels in the brains of *Sst*_*1*_*/Sst*_*4*_ dKO mice and SST knock out (*Srif* KO) mice and found that ENSA levels were significantly elevated in the cortex and hippocampus of both mouse strains (Supplementary Fig. 2A–C). In addition, immunohistochemical analyses indicated that ENSA-positive signals were heightened in the cortical and hippocampal CA1 and CA3 regions in these animals (Supplementary Fig. 2D–G). Taken together, these results suggest that ENSA functions as a negative NEP regulator downstream of SST signaling.

### Activation of NEP by genetic deficiency of ENSA in vivo

ENSA, an endogenous blocker of SUR1 which is a regulatory subunit of K_ATP_ channel, is highly expressed in brain, skeletal muscle and pancreas [39, 40]. Although we found that ENSA is a negative regulator of NEP in vitro, the function of ENSA in vivo is largely unknown. We therefore generated ENSA knock out (*Ensa* KO) mice using CRISPR/Cas9 technology. Dual adjacent single-guide RNAs (sgRNAs) were designed that targeted exon 1 of the *Ensa* gene including the initiation codons (Supplementary Fig. S3A). This strategy facilitates CRISPR-mediated genome targeting [41]. We injected sgRNA1-*Ensa*-Exon1 (30 ng/ml) and sgRNA2-*Ensa-*Exon1 (30 ng/ml) together with *Streptococcus pyogenes* Cas9 (SpCas9) mRNA (60 ng/ml) into WT mouse zygotes. Sanger sequencing analysis and PCR-based genotyping indicated the deletion of exon 1, including the initiation codons (Supplementary Fig. S3B and C). Expression of ENSA in homozygous F2 mutant lines, generated by crossbreeding the heterozygous F1 mutant lines with each other, was fully deleted (Supplementary Fig. S3D and E). To assess the off-target effects of CRISPR/Cas9 in the founder, we searched for potential off-target sites using COSMID [33], with 53 candidate sites being identified (Supplementary Table S5). Of note, there was no off-target mutation on chromosome 3, which contains the *Ensa* gene. PCR-based genotyping and Sanger sequencing analyses for each candidate site revealed that founder had an off-target mutation in an intergenic region of chromosome 2 which was removed by backcrossing with WT mice (Supplementary Fig. S3F and G).

**Fig. 2:**
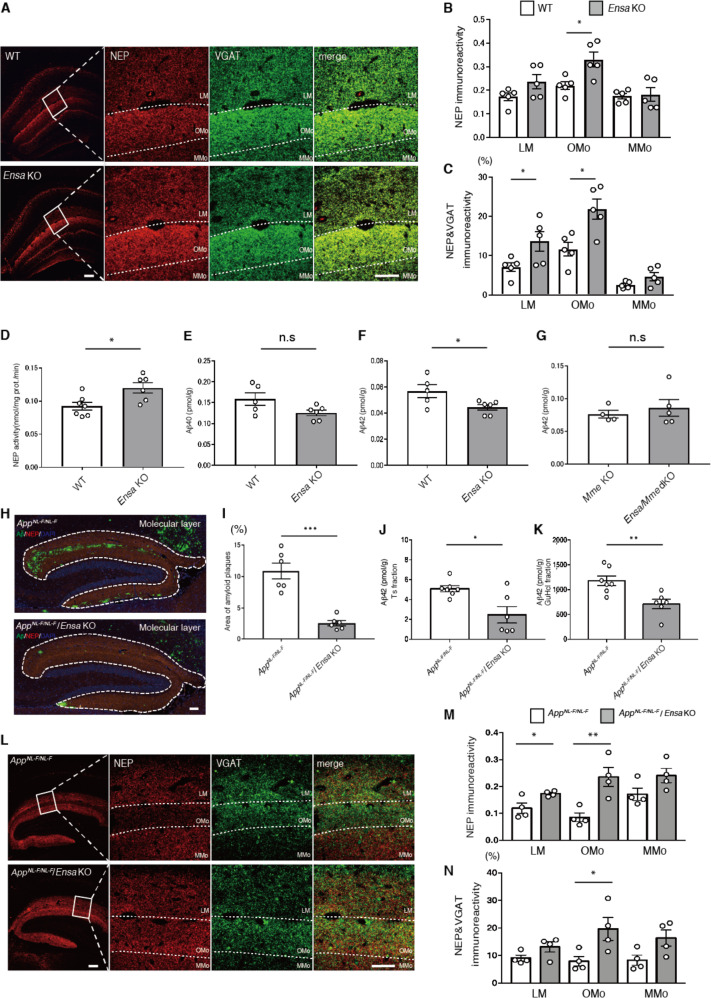
Elevation of NEP activity in *Ensa* KO mice. **A** Immunostaining of NEP (Red) and VGAT (Green) from hippocampi of 3-month-old WT and *Ensa* KO mice. Scale bar is 100 µm in low magnification image and 50 µm in high-magnification image. **B** Statistical analysis of NEP immunoreactivity (*n* = 5 for each group). LM: lacunosum-molecular layer, Omo: Outer molecular layer and MMo: middle molecular layer. **C** Statistical analysis of colocalized NEP and VGAT signals (*n* = 5 for each group). **D** NEP activity in membrane fractions from hippocampi of 3-month-old WT and *Ensa* KO mice (WT: *n* = 7, *Ensa* KO: *n* = 6). **E**. Aβ_40_ ELISA of hippocampi of 3-month-old WT and *Ensa* KO mice (WT: *n* = 5, *Ensa* KO: *n* = 6). **F**. Aβ_42_ ELISA of hippocampi of 3-month-old WT and *Ensa* KO mice (WT: *n* = 5, *Ensa* KO: *n* = 6). **G** Aβ_42_ ELISA of hippocampi of 3-month-old *Mme* KO mice and *Ensa*/*Mme* dKO (*Mme* KO: *n* = 4, *Ensa*/*Mme* dKO: *n* = 5). **H** and **I**. Immunostaining of Aβ (Green), NEP (Red) and DAPI (blue) from 18-month-old *App*^*NL-F*^ and *App*^*NL-F*^/*Ensa* KO mice. Statistical analysis of amyloid plaque area in 18-month-old *App*^*NL-F*^ and *App*^*NL-F*^/*Ensa* KO mice (*n* = 6 for each group). Scale bar is 100 µm. **J** Aβ_42_ ELISA of Tris-HCl-buffered saline-soluble (Ts) hippocampal fractions from 18-month-old *App*^*NL-F*^ and *App*^*NL-F*^/*Ensa* KO mice (*App*^*NL-F*^: n = 7, *App*^*NL-F*^/*Ensa* KO: *n* = 6). **K** Aβ_42_ ELISA of guanidine-HCl-soluble (GuHCl) hippocampal fractions from *App*^*NL-F*^ and *App*^*NL-F*^/*Ensa* KO mice (*App*^*NL-F*^: *n* = 7, *App*^*NL-F*^/*Ensa* KO: *n* = 6). **L** Immunostaining of NEP (Red) and VGAT (Green) in hippocampi of 18-month-old *App*^*NL-F*^ and *App*^*NL-F*^/*Ensa* KO mice. Scale bar is 100 µm in low-magnification image and 50 µm in high-magnification image. **M** Statistical analysis of NEP immunoreactivity (*n* = 4 for each group). **N** Statistical analysis of colocalized NEP and VGAT signals (*n* = 4 for each group). Data represent the mean ± SEM. *P < 0.05, **P < 0.01, ***P < 0.001 (Student’s or Welch’s *t* test).

NEP, a pH-sensitive enzyme, efficiently degrades Aβ_42_ at the presynaptic region where the pH is neutral rather than inside secretory vesicles where the pH is acidic [15], without altering Aβ_40_ levels under different pH conditions. To determine whether a deficiency of ENSA affects the localization of NEP, we used immunohistochemistry to analyze the expression of NEP and vesicular GABA transporter (VGAT; a presynaptic marker) in the brains of *Ensa* KO mice. We found that NEP signals in the outer molecular layer of the dentate gyrus (OMo) were significantly increased (Fig. 2A and B), and that colocalization of NEP and VGAT was increased in both the lacunosum molecular layer (LM) and OMo (Fig. 2A and C). Next, we measured NEP activity in hippocampal membrane fractions from *Ensa* KO mice and found that a deficiency of ENSA paralleled that of a significantly increased NEP activity (Fig. 2D). We then quantified Aβ_40_ and Aβ_42_ levels in the hippocampi of *Ensa* KO mice by enzyme-linked immunosorbent assay (ELISA) and found that Aβ_42_ levels were significantly reduced compared to those of control mice (Fig. 2F), with Aβ_40_ levels remaining relatively stable (Fig. 2E). This reduction of Aβ_42_ was reproduced in another line of *Ensa* KO mice (*Ensa* KO #2) that was generated by CRISPR/Cas9 with different sgRNAs (Supplementary Fig. S4). Aβ_42_ levels in ENSA and NEP double knock out (*Ensa/Mme* dKO) mice did not differ from those of single *Mme* KO mice (Fig. 2G), indicating that NEP mediated the reduction of Aβ_42_ in the hippocampi of *Ensa* KO mice.

We next investigated whether the deficiency of ENSA affected the processing of Aβ production or expression of other Aβ-degrading enzymes. We performed Western blot analysis of full-length APP, its C-terminal fragments generated by α-secretase (CTF-α) and β-secretase (CTF-β), insulin-degrading enzyme (IDE), and endothelin converting enzyme 1 (ECE-1). No significant differences were observed in the expression levels of these proteins and ratio of the CTF-β/α fragments (Supplementary Fig. S3H and I).

To examine the effect of ENSA deficiency on Aβ pathology, we next crossbred *Ensa* KO mice with *App*^*NL-F/NL-F*^ Knock-in (*App*^*NL-F*^) mice. *App*^*NL-F*^ mice harbor two familial AD-causing mutations (Swedish (KM670/671NL) and Beyreuther/Iberian (I716F)) in the endogenous *App* gene as well as humanized Aβ sequences, and develop amyloid pathology in the hippocampus and cortex from around 6 months of age [28]. Immunohistochemical analyses using specific antibodies against the unmodified amino-terminus Aβ, N1D, and modified amino-terminus of Aβ, Ν3(pΕ), respectively [42], revealed that both N1D- and Ν3(pΕ)−positive amyloid depositions in hippocampal molecular layer area were significantly reduced in *App*^*NL-F*^/*Ensa* KO mice, where NEP expression was elevated (Fig. 2H and I, and Supplementary Fig. S3J and K). Aβ ELISA on the hippocampi of *App*^*NL-F*^/*Ensa* KO mice also showed significant reduction of Aβ_42_ levels (Fig. 2J and K). We consistently found that NEP expression in the LM and OMo of *App*^*NL-F*^/*Ensa* KO mice was upregulated, particularly in the presynaptic region of OMo (Fig. 2L-N). Taken together, these observations suggest that ENSA is a negative regulator of NEP in vivo and that a deficiency of ENSA attenuates Aβ pathology by allowing NEP activity to be upregulated.

To explore the involvement of ENSA in the etiology of AD, we analyzed ENSA levels in an AD mouse model and in postmortem brain of patients with AD. Western blot analyses revealed that ENSA expression was significantly increased in the cortices and hippocampi of *App*^*NL-F*^ mice at 24 months (Supplementary Fig. 5A-D). In immunohistochemical analyses, ENSA signals in the cerebral cortices and hippocampal CA1 and CA3 regions of *App*^*NL-F*^ mice were also increased at 24 months (Supplementary Fig. 5E and F). Consistent with these observations, ENSA levels were markedly increased in the cortices of patients with AD (Supplementary Fig. 5G-J).

### Feedback mechanism regulating NEP activity

**Fig. 3:**
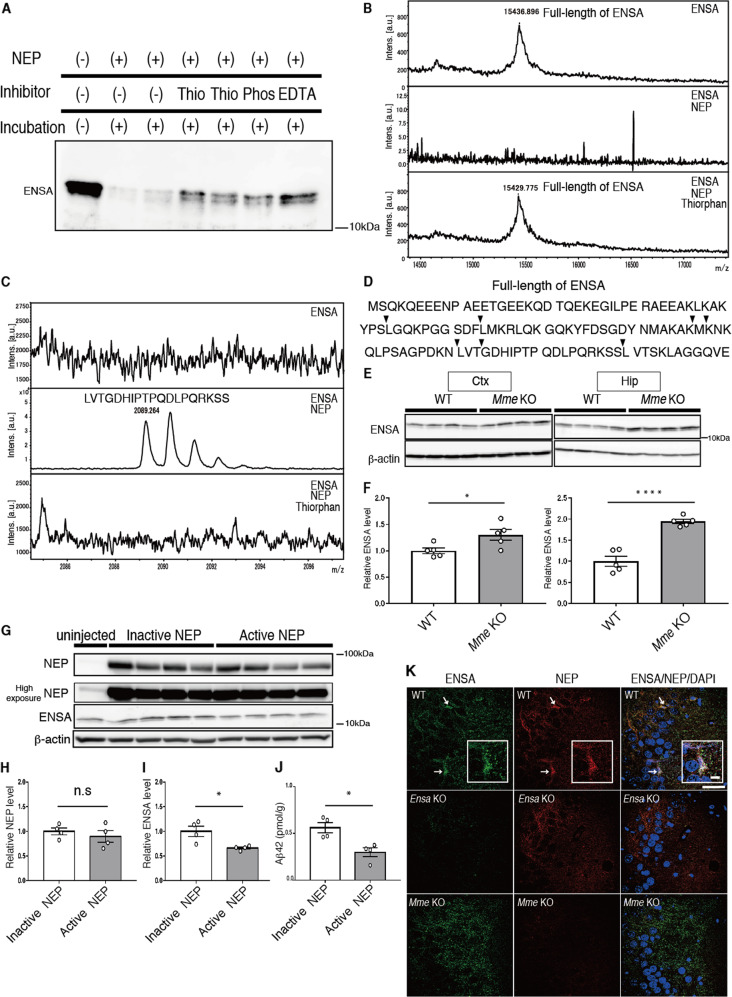
Identification of ENSA as a substrate for NEP. **A** Immunoblotting of ENSA incubated with or without NEP and mentioned inhibitors for 24 h at 37 ˚C. Thio: Thiorphan, Phos: Phosphoramidon. **B** Specific peak of full-length of ENSA after incubation with or without NEP and thiorphan. **C** Specific peak of cleaved ENSA after incubation with or without NEP and thiorphan. **D** Sequence of full-length of ENSA. Arrowheads indicate cleavage site by NEP. **E**, **F** Immunoblotting of ENSA from cortices and hippocampi of 6-month-old WT and *Mme* KO mice. Values indicated in the graph show ENSA band intensities normalized to that of β-actin (*n* = 5 for each group). **G**–**I** Immunoblotting of (**H**) NEP and (**I**) ENSA from hippocampi of 3-month-old WT mice after overexpression of active or inactive mutant NEP by SFV gene expression system. Values indicated in the graph show NEP and ENSA band intensities normalized to that of β-actin (*n* = 4 for each group). **J** Aβ_42_ ELISA of Tris-HCl-buffered saline-soluble fractions containing 1% Triton-X from hippocampi of WT mice after overexpression of active or inactive mutant NEP by the SFV gene expression system (*n* = 4 for each group). **K** Immunostaining of ENSA (Green), NEP (Red) and DAPI (Blue) in CA3 from 3-month-old WT, *Ensa* KO and *Mme* KO mice. Scale bar is 50 µm in low-magnification image and 10 µm in high-magnification image. White arrows indicate colocalized signals. Data represent the mean ± SEM. *P < 0.05, ****P < 0.0001 (Student’s or Welch’s *t* test).

SST, an endogenous regulator of NEP, is degraded by NEP in a substrate-dependent feedback manner [15, 43]. We hypothesized that NEP might also directly degrade ENSA in a similar feedback manner. Co-incubation of recombinant ENSA with NEP resulted in a remarkable decrease in ENSA levels (Fig. 3A). Several NEP inhibitors such as thiorphan, phosphoramidon and EDTA attenuated this effect, indicating that NEP degrades ENSA in vitro (Fig. 3A). To identify the NEP-mediated cleavage sites in ENSA, we performed MALDI-TOF analysis after incubation of recombinant ENSA with NEP. Multiple ENSA fragments were detected in the NEP-treated sample, but not in a sample treated in the presence of thiorphan (Fig. 3B and C, and Supplementary Fig. S6). We determined the amino acid sequences of these fragments by LC-MS/MS analysis (Supplementary Table S8), and found that NEP partially cleaved ENSA on the amino-terminal side of hydrophobic amino acids in a manner similar to that of other NEP substrates, including Aβ (Fig. 3D).

ENSA levels in the brains of *Mme* KO mice were subsequently examined by Western blotting and we observed that ENSA was significantly increased in the cortices and hippocampi of these animals (Fig. 3E and F). We then overexpressed WT and inactivated mutant NEP in the hippocampi of WT mice using the Semliki Forest virus gene expression system [34]. Exogenously expressed active NEP, but not the inactive mutant, significantly lowered ENSA levels as well as Aβ_42_ levels (Fig. 3G–J). We also performed immunohistochemical analyses of ENSA and NEP, and found that these proteins were colocalized in the CA3 region (Fig. 3K). These results suggest that NEP directly contributes to the degradation of ENSA both in vitro and in vivo and that NEP activity is regulated by a substrate-dependent feedback mechanism.

### K_ATP_ channel subtype SUR1/Kir6.2 modulates NEP action

**Fig. 4:**
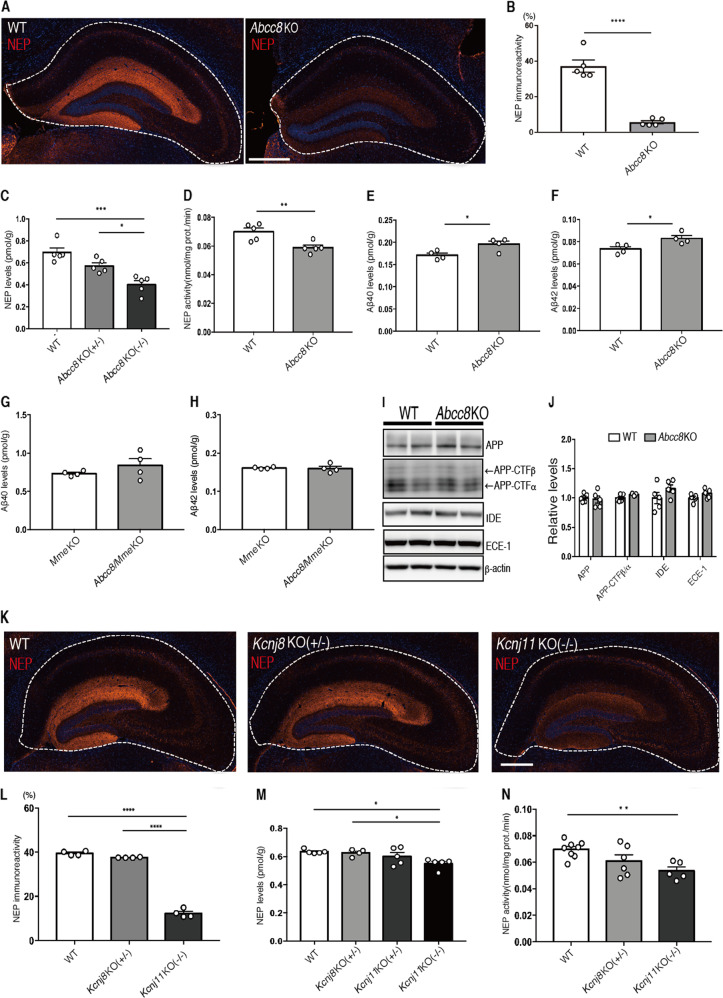
SUR1/Kir6.2 regulates NEP expression and activity. **A**, **B** Immunostaining of NEP in hippocampi of WT and *ABCC8* KO mice (*n* = 6 for each group). Scale bar represents 500 µm. **C** NEP ELISA with hippocampi of WT, heterozygous and homozygous *ABCC8* KO mice (*n* = 5 for each group). **D** NEP activity in hippocampi of 3-month-old WT and *Abcc8* KO mice (*n* = 5 for each group). **E** Aβ_40_ ELISA of hippocampi from 3-month-old WT and *Abcc8* KO mice (*n* = 4 for each group). **F** Aβ_42_ ELISA of hippocampi from 3-month-old WT and *Abcc8* KO mice (*n* = 4 for each group). **G** Aβ_40_ ELISA of hippocampi from 3-month-old *Mme* KO and *Abcc8*/*Mme* dKO mice (*n* = 4 for each group). **H** Aβ_42_ ELISA of hippocampi from 3-month-old *Mme* KO and *Abcc8*/*Mme* dKO mice (*n* = 4 for each group). **I** Immunoblotting of APP, CTFs, IDE and ECE-1 in hippocampi of 3-month-old WT and *Abcc8* KO mice. **J** Values indicated in graphs show band intensities for APP, CTFs, IDE, and ECE-1 normalized to that of β-actin (*n* = 5 for each group). **K**, **L**. Immunostaining of NEP in hippocampi of WT and heterozygous *Kcnj8* KO and homozygous *Kcnj11* KO mice (*n* = 4 for each group). Scale bar represents 500 µm. **M** NEP ELISA with hippocampi from WT and heterozygous *Kcnj8* KO and homozygous *Kcnj11* KO mice (*n* = 5 for each group). **N** NEP activity from hippocampi of 3-month-old WT and heterozygous *Kcnj8* KO and homozygous *Kcnj11* KO mice (WT: *n* = 8, *Kcnj8* KO: *n* = 6, *Kcnj11* KO: *n* = 5). In (**B**), (**D**), (**E**), (**F**), the data represent the mean ± SEM. *P < 0.05, **P < 0.01, ****P < 0.0001 (Student’s *t* test). In (**C**), (**L**), (**M**), (**N**), the data represent the mean ± SEM.**P < 0.01, ***P < 0.001, ****P < 0.0001 (one-way ANOVA with Turkey’s multiple comparison test).

The K_ATP_ channel is composed of four regulatory sulfonylurea receptor subunits (SUR1, SUR2A or SUR2B) and four inwardly rectifying K^+^ channel (Kir6.1 or Kir6.2) subunits as heteromeric complexes. Its structural heterogeneity leads to a variety of functions in different tissues. The SUR1/Kir6.1 or Kir6.2 and SUR2B/Kir6.2 K_ATP_ channel subtypes are mainly expressed in the brain [44], whereas the Kir6.1 subunit is dominantly present in astrocytes, and the Kir6.2 subunit in neurons [45, 46]. Given that ENSA has been proposed as a ligand of SUR1, we assessed whether SUR1 regulates NEP expression and/or activity in vivo. Immunohistochemical analyses and NEP ELISA revealed that the expression level of NEP in the hippocampi of SUR1 knockout (*Abcc8* KO) mice was strikingly reduced (Fig. 4A–C). In addition, NEP activity in the hippocampi of *Abcc8* KO mice was also significantly decreased compared to that of WT mice (Fig. 4D), resulting in a significant increase of both Aβ_40_ and Aβ_42_ levels (Fig. 4E and F). In contrast, Aβ_40_ and Aβ_42_ levels in SUR1 and NEP double knockout (*Abcc8/Mme* dKO) mice did not differ from those of single *Mme* KO mice (Fig. 4G and H). No significant differences were observed in the expression levels of APP, CTF-β/α, IDE, or ECE-1 (Fig. 4I and J), indicating that NEP mediated the increase of both Aβ_40_ and Aβ_42_ levels in the hippocampi of *Abcc8* KO mice. Indeed, genetic deficiency of SUR1 also significantly lowered NEP positive signals in the hippocampal molecular layer of *App*^*NL-F*^/*Abcc8* KO mice, thereby elevating the amyloid deposition (Supplementary Fig. S7A-C).

Next, to identify which Kir subunit regulates NEP activity, we evaluated the expression and activity of NEP in the hippocampi of both kir6.1 knockout (*Kcnj8* KO) and Kir6.2 knockout (*Kcnj11* KO) mice. Because homozygous *Kncj8* KO mice are prone to premature death, and most die within 1.5 months of birth [27], we employed the heterozygous mutants (*Kncj8*^+/−^ KO) in these experiments. Immunohistochemical analyses and NEP ELISA revealed a significant reduction of NEP levels in *Kcnj11* KO mice (Fig. 4K–M). We also measured lower levels of NEP activity in the hippocampi of *Kncj11* KO mice than that of WT mice (Fig. 4N). These results suggest that the SUR1/Kir6.2 K_ATP_ channel subtype is the main regulator of NEP action in the brain.

To determine the impact of the SUR1/Kir6.2 subtype on the etiology of AD, we analyzed the transcripts of each K_ATP_ channel component in datasets from human postmortem brains, with or without AD, obtained from the Gene Expression Ominibus (GEO) of the National Center for Biotechnology Information (NCBI). The GSE15222 dataset, which included 135 controls and 106 late-onset AD samples, revealed that *ABCC8* gene expression was significantly reduced among the K_ATP_ channel components in AD patients (Supplementary Fig. S8A, and Supplementary Table S10) [35]. Consistently, this reduction was reproduced in other cohort datasets (GSE95587 and GSE125583) (Supplementary Fig. S8B and C, and Supplementary Table S10) [36, 37]. Furthermore, Van Rooji *et al*., also indicated that robust reductions of both *ABCC8* and *KCNJ11* mRNA expression levels were observed in AD patients (Supplementary Table S10) [38]. According to gene expression analyses with differentiating Braak stages in both GSE95587 and GSE125583 datasets, reductions of *ABCC8* and *KCNJ11* mRNA levels were observed with the progression of AD (Supplementary Fig. S8D-G, and Supplementary Table S11).

### Improvement of Aβ pathology and memory function by diazoxide in an AD mouse model

**Fig. 5:**
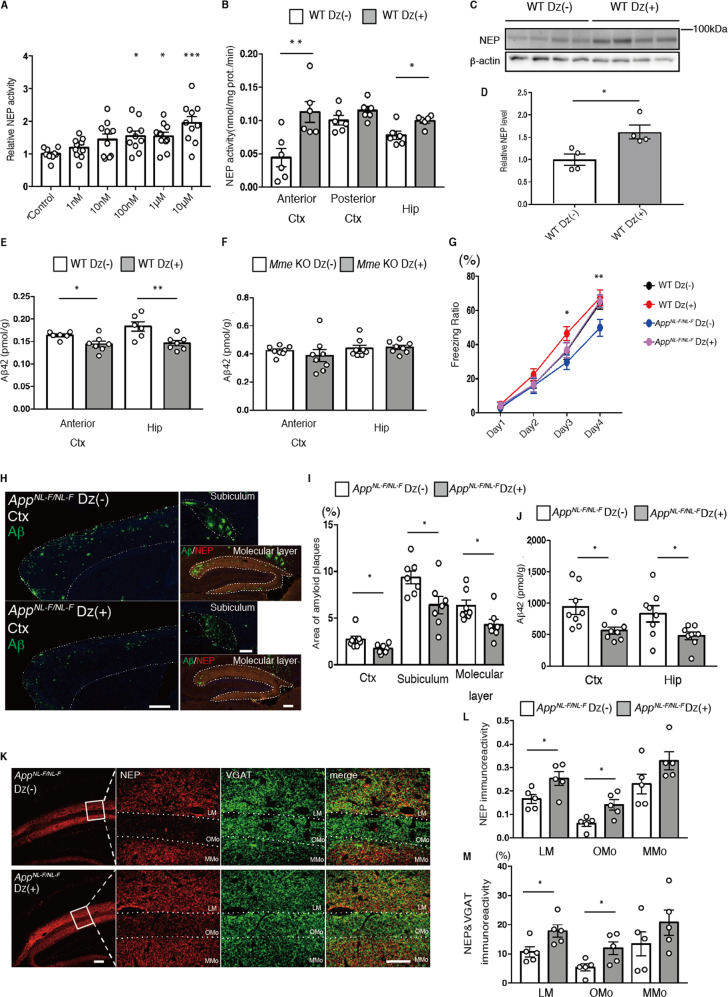
Improvement of Aβ pathology and memory function in *App*^*NL-F*^ mice via enhancement of NEP activity by Dz treatment. **A** NEP activity after treatment of co-cultured neurons for 24 h with different doses of diazoxide (Dz) (*n* = 9–10 for each group). **B** NEP activity in membrane fractions from anterior cortex (Ctx), posterior Ctx and hippocampus (Hip) of 4-month-old WT mice treated with or without Dz (*n* = 6 for each group). **C**, **D**. Immunoblotting of NEP in anterior Ctx of 4-month-old WT mice treated with or without Dz. Values indicated in the graph Values indicated in the graph show NEP band intensities normalized to that of β-actin (*n* = 4 for each group). **E** Aβ_42_ ELISA of GuHCl fractions from anterior Ctx and Hip of 4-month-old WT mice with or without Dz (Dz (–): *n* = 6, Dz (+): *n* = 7). **F** Aβ_42_ ELISA of GuHCl fractions from anterior Ctx and Hip of 6-month-old *Mme* KO mice with or without Dz (*n* = 8 for each group). **G** Freezing ratio of 18-month-old WT and *App*^*NL-F*^ mice treated with or without Dz (WT Dz (–): *n* = 12, WT Dz (+): *n* = 13, *App*^*NL-F*^ Dz (–): *n* = 10, *App*^*NL-F*^ Dz (+): *n* = 11). **H**, **I** Immunostaining of Aβ (Green) and NEP (Red) in Ctx, Subiculum and Molecular layer from 18-month-old *App*^*NL-F*^ with or without Dz (*n* = 7 for each group). Scale bar in cortical image = 500 µm and hippocampal image = 200 µm. **J** Aβ_42_ ELISA of GuHCl fractions from cortices and hippocampi of 18-month old *App*^*NL-F*^ with or without Dz (*n* = 8 for each group). **K** Immunostaining of NEP (Red) and VGAT (Green) in hippocampi from 18-month old *App*^*NL-F*^ with or without Dz. Scale bar is 100 µm in low-magnification image and 50 µm in high-magnification image. L. Statistical analysis of immunoreactivity of NEP (*n* = 5 for each group). LM: lacunosum-molecular layer, Omo: Outer molecular layer and MMo: middle molecular layer. **M** Statistical analysis of colocalized signals of NEP and VGAT (*n* = 5 for each group). In (**A**), the data represent the mean ± SEM. *P < 0.05, ***P < 0.001 (one-way ANOVA with Dunnett’s post-hoc test). In (**B**), (**D**), (**E**), (**I**), (**J**), (**L**), (**M**), the data represent the mean ± SEM. *P < 0.05, **P < 0.01, (Student’s *t* test). In (**G**) the data represent the mean ± SEM. On day 3, WT Dz (+) vs *App*^*NL-F*^ Dz (–) *P < 0.05. On day 4, WT Dz (–) vs *App*^*NL-F*^ Dz (–) *P < 0.05, WT Dz (+) vs *App*^*NL-F*^ Dz (–) **P < 0.01, *App*^*NL-F*^ Dz (–) vs *App*^*NL-F*^ Dz (+) *P < 0.05 (two-way ANOVA with Turkey’s multiple comparison test).

On the basis of these observations, we hypothesized that agonist stimulation of SUR1/Kir6.2 in brain might be beneficial for the prevention of AD via the NEP upregulation. To investigate whether diazoxide (Dz), a well-known K_ATP_ channel activator with a high affinity for SUR1 [47], regulates NEP action, we incubated co-cultured primary neurons with Dz, and found that NEP is activated in a dose-dependent manner (Fig. 5A). As Dz has been reported cross the blood brain barrier [48–50], we treated WT mice by oral administration of Dz for 1 month. This treatment significantly increased NEP activity in the anterior cortex and hippocampus of these animals (Fig. 5B), with elevated levels of NEP expression also seen in the anterior cortex (Fig. 5C and D). In line with this, Dz significantly lowered Aβ_42_ levels in the anterior cortex and hippocampus, where NEP was activated (Fig. 5E), whereas Dz treatment had no effect on Aβ_42_ levels in the anterior cortex and hippocampus of *Mme* KO mice (Fig. 5F). These results suggest that Dz decreased Aβ_42_ levels in a NEP-mediated manner.

We next investigated the therapeutic effect of Dz on *App*^*NL-F*^ mice by carrying out contextual fear-conditioning tests to assess memory function after 3 months of Dz treatment from the age of 15 months. Dz treatment recovered the freezing ratio of *App*^*NL-F*^ mice to a level comparable to that of WT mice (Fig. 7G). We also performed open field maze tests to assess the anxiety phenotype as it has been shown that anxiety may affect performance in spatial memory tasks [51]. Dz treatment in WT and *App*^*NL-F*^ mice did not alter the amount of time spent in the central region (Supplementary Fig. S9A), indicating that Dz had no effect on psychological status. Dz treatment also decreased both N1D- and Ν3(pΕ)−positive amyloid depositions in the cortex, subiculum and hippocampal molecular layer (Fig. 5H and I and Supplemental Fig. S9D and E). Aβ_42_ levels in the cortices and hippocampi of Dz-treated *App*^*NL-F*^ mice were also significantly reduced (Fig. 5J). Immunohistochemical analyses indicated an increase of NEP expression in the anterior cortex of these mice (Supplementary Fig. S9B and C). In addition, colocalized signals of NEP and VGAT were also increased in the presynaptic regions of the hippocampal LM and OMo (Fig. 5K-M). Dz had no effect on behavior (Supplementary Fig. S9F and G) or Aβ pathology in *App*^*NL-F*^/*Mme* KO mice (Supplementary Fig. S9H and I). Taken together, these results suggest that Dz improves Aβ pathology and memory impairment in *App*^*NL-F*^ mice by upregulating NEP activity.

## Discussion

In the present study, we used in vitro and in vivo experimental paradigms to identify ENSA as a negative regulator of NEP activity downstream of SST signaling. To further understand the mechanism of NEP regulation downstream of ENSA, we revealed that, of the multiple possible subtypes of K_ATP_ channels, SUR1/Kir6.2 regulates NEP action. Finally, agonist stimulation of SUR1 by Dz prevented Aβ deposition via the upregulation of NEP, thereby improving memory function in *App*^*NL-F*^ mice.

SST mRNA levels were reported to decrease in brain with aging and in AD [16–21]. As such, ENSA, a downstream protein of SST signaling, may be related to the etiology of AD. Indeed, we showed elevation of ENSA levels in *App*^*NL-F*^ mice as well as in AD patients (Supplementary Fig. 5). Moreover, in vitro and in vivo experiments revealed that NEP degraded ENSA as a substrate, suggesting that NEP and ENSA form a negative feedback loop. This hypothesis is based on the fact that opioids and substance P, cell-specific ligands in monocytes and bone marrow cells, respectively [52, 53], regulate NEP via a feedback mechanism. It is possible that Aβ and ENSA compete against each other in the NEP-mediated degradation, additively exacerbating this feedback-loop and inducing a vicious cycle.

A selective agonist of the K_ATP_ channel such as Dz could serve as a beneficial approach to break this vicious cycle given that it is used as a drug for antihypertensive and hypoglycemic properties, and has the potential in the preclinical setting to improve Aβ pathology and behavioral abnormalities in AD [48, 49]. A previous study showed that Dz treatment reduced the extracellular accumulation of Aβ in 3xTg mice which display both amyloid and tau pathology due to overexpression of mutated *APP* and *MAPT* genes on a mutant *PSEN1* background [54, 55]. The mechanism by which Dz attenuated Aβ plaque deposition was, however, unclear. Our findings indicate that Dz reduced amyloid deposition in *App*^*NL-F*^ mice via the regulation of NEP activity in the anterior cortex and hippocampus. This regional selectivity of NEP action by Dz may be dependent on the dopaminergic system in the brain. The K_ATP_ channel is highly expressed in dopaminergic neurons in the midbrain and regulates dopamine release. These neurons project to the frontal cortex and hippocampus [56–61]. Recently we confirmed that dopamine regulates NEP expression and/or activity in the frontal cortex and hippocampus regions in *App*^*NL-F*^ mice. To further elucidate the mechanism for the regulation of NEP activity, it will be necessary to investigate pathways downstream of SUR1/Kir6.2. Likewise, it is important to develop a more specific opener of SUR1/Kir6.2 to avoid off-target effects given that different K_ATP_ channel subtypes are expressed in vascular smooth muscle cells, cardiac muscle cells and pancreatic β-cells [62]. In addition to promoting NEP-mediated Aβ degradation, K_ATP_ channel agonists may have beneficial effects in AD. Dz treatment prevents Aβ-induced neurotoxicity induced by oxidative stress and inflammatory damage and also shows neuroprotective effects against apoptosis in vitro [63–67]. Compared to Aβ-targeting immunotherapies, synthetic agonists for the K_ATP_ channel are less expensive and would be more acceptable in aging societies around the world.

Taken together, we have demonstrated a new preventive approach at the preclinical stage of AD based on the function of ENSA. This negative regulator of NEP and K_ATP_ channel could be a new therapeutic target for lowering Aβ.

## Supplementary information

Supplementary Materials

Supplementary Materials
