# Supplementary material for: Somatostatin-evoked Aβ catabolism in the brain: Mechanistic involvement of α-endosulfine-KATP channel pathway
Source: Mol Psychiatry. 2021 Nov 4;27(3):1816–28. doi: 10.1038/s41380-021-01368-8 (PMC9095489; doi:10.1038/s41380-021-01368-8)
Supplement: Supplementary file 1 — Supplementary Materials [file 41380_2021_1368_MOESM1_ESM.pdf]

Supplementary Materials

fig. S1

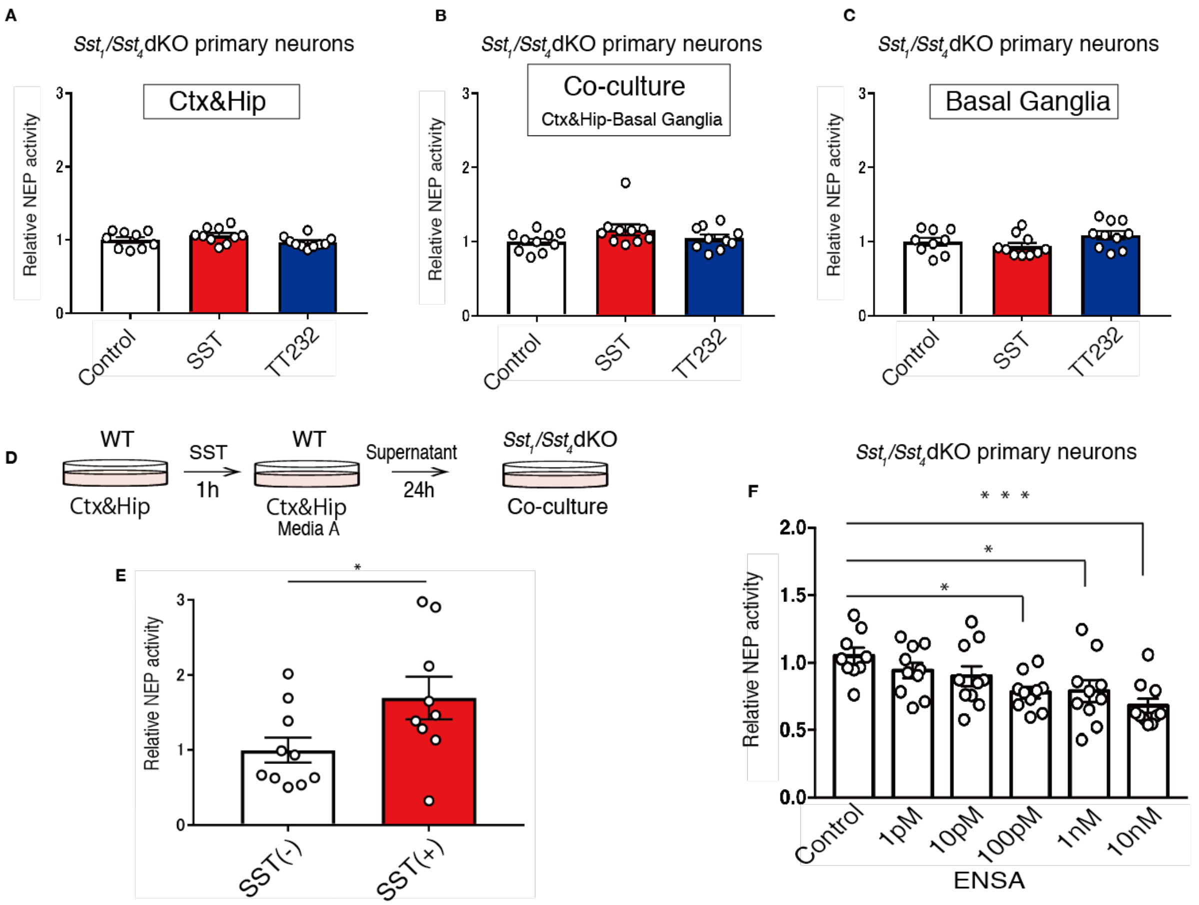

Supplementary Figure S1. NEP activity in primary neurons from *Sst_1/Sst_4* dKO mice.

A-C. NEP activity after treatment of primary neurons derived from *Sst_1*/*Sst_4* dKO mice with 1 μM SST or TT232 for 24 hours. (A) Cortical/hippocampal (Ctx&Hip) neurons (n = 9-10 wells per treatment), (B) co-cultured neurons (n = 10 wells per treatment), and (C) basal ganglia neurons (n = 9-10 wells per treatment) were used. D and E. NEP activity of co-cultured neurons from *Sst_1*/*Sst_4* dKO mice after replacement of the culture medium with conditioned media derived from SST-treated Ctx&Hip neurons from WT mice (n = 9-10 for each group). F. NEP activity of co-cultured neurons derived from *Sst_1*/*Sst_4* dKO mice after treatment with different doses of recombinant ENSA protein (n = 9-10 for each group). In (E)**,** the data represent the mean ±SEM. *P<0.05 (Student's *t*-test). In (F)**,** the data represent the mean ±SEM. *P<0.05, **P<0.01, ***P<0.001 (one-way ANOVA with Dunnett's post-hoc test).

fig. S2

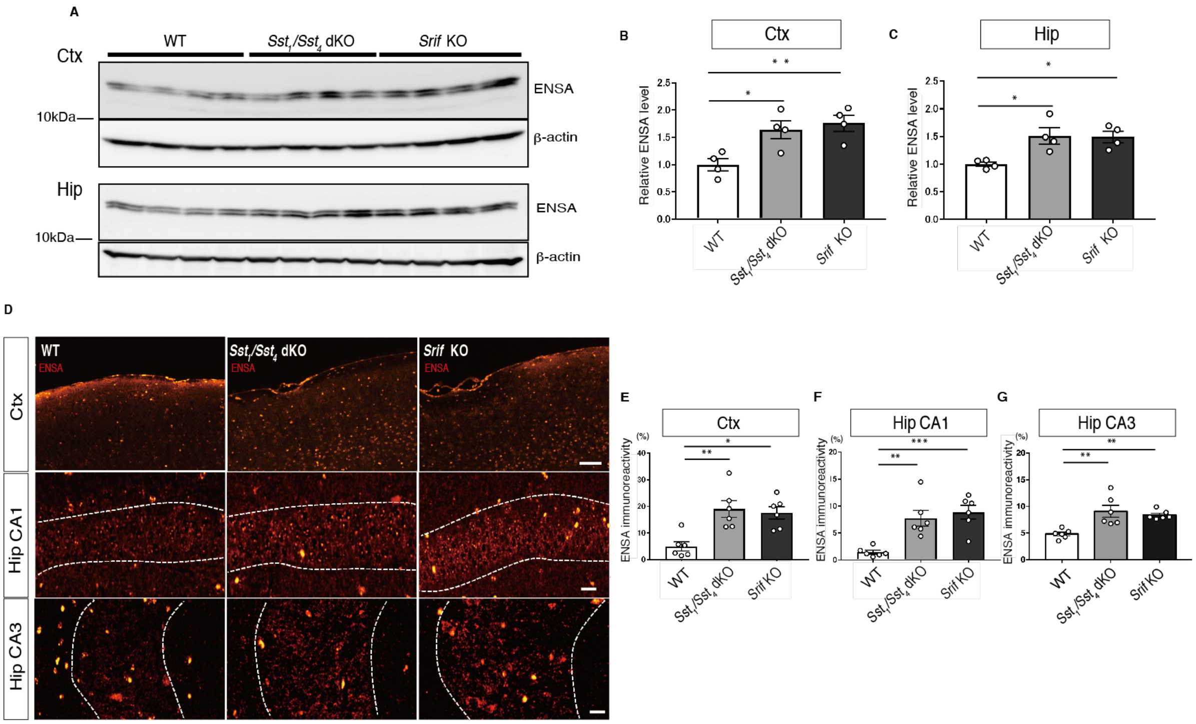

Supplementary Figure S2 ENSA levels in *Sst1/Sst4*- and *Srif*-deficient mice.

A-C. Immunoblotting of ENSA in the (B) cortices and (C) hippocampi from 3-month-old WT, *Sst1/Sst4* dKO and *Srif* KO mice (n = 4 for each group). Values indicated in the graph show ENSA band intensities normalized to that of β-actin. D-G. Immunostaining of ENSA in the (E) cortices and (F) hippocampal CA1 and (G) CA3 regions from 3-month-old WT, *Sst1/Sst4* dKO, and *Srif* KO mice (n = 6 for each group). Scale bar is 200 µm in cortical image and 20 µm in hippocampal image. Data represent the mean ±SEM. $*P<0.05$, $**P<0.01$, $***P<0.001$ (one-way ANOVA with Dunnett's post-hoc test).

fig. S3

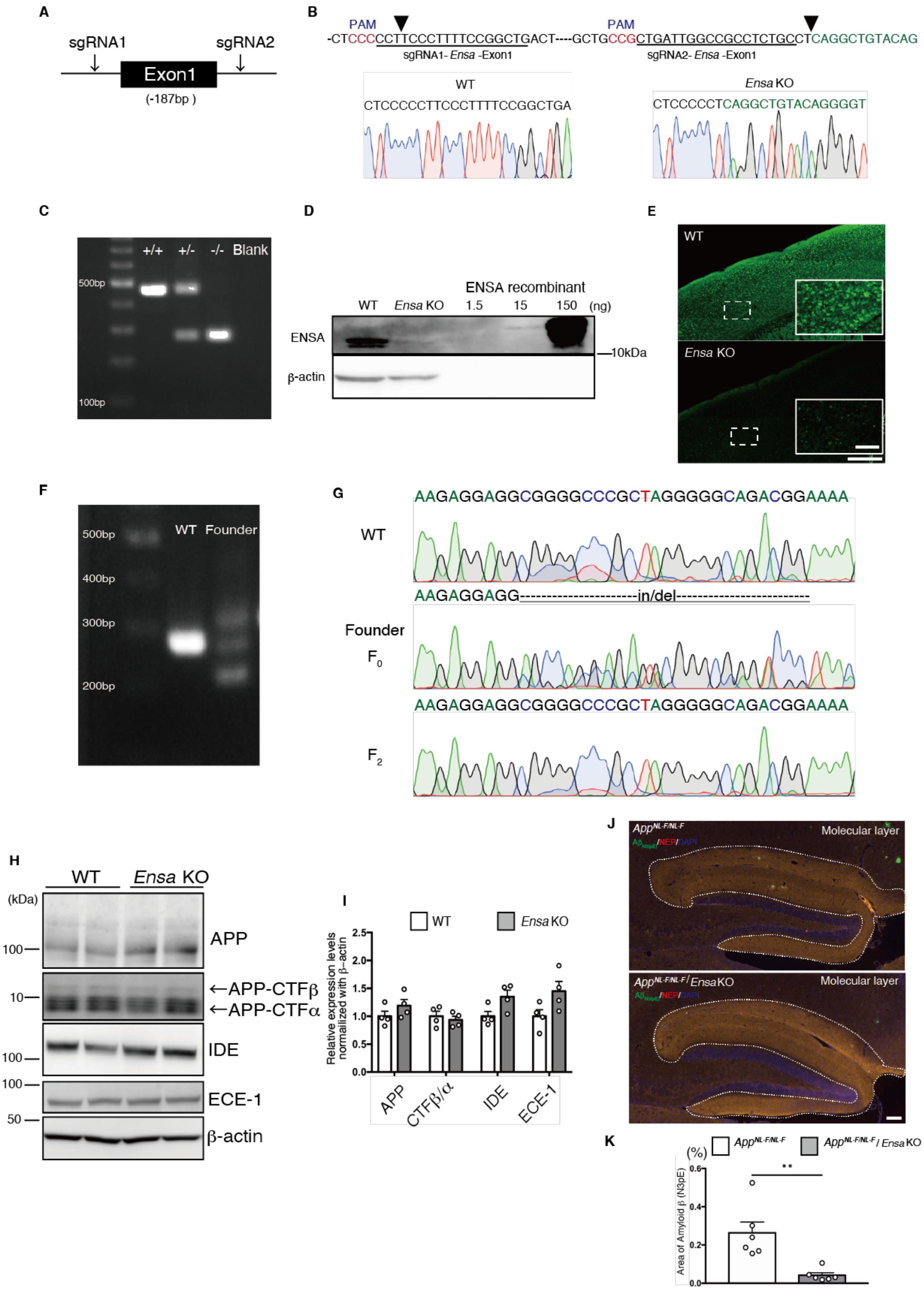

A
sgRNA1
sgRNA2
Exon1
(-187bp)
B
PAM
-CTCCCCCTTCCCTTTTCCGGCTGACT----GCTGCCGCTGATTGGCCGCCTCTGCCTCAGGCTGTACAG
sgRNA1- Ensa -Exon1
sgRNA2- Ensa -Exon1
WT
CTCCCCCTTCCCTTTTCCGGCTGA
Ensa KO
CTCCCCCTCAGGCTGTACAGGGGT
C
+/+
+/-
-/-
Blank
500bp
100bp
D
ENSA recombinant
WT
Ensa KO
1.5
15
150
(ng)
ENSA
β-actin
10kDa
E
WT
Ensa KO
F
WT
Founder
500bp
400bp
300bp
200bp
G
AAGAGGAGGCGGGGCCCGCTAGGGGGCAGACGGAAAA
WT
AAGAGGAGG---------------------in/del-----------------------
Founder
F_0
AAGAGGAGGCGGGGCCCGCTAGGGGGCAGACGGAAAA
F_2
H
WT
Ensa KO
(kDa)
100
APP
10
←APP-CTFβ
←APP-CTFα
100
IDE
100
ECE-1
50
β-actin
I
WT
Ensa KO
Relative expression levels normalized with β-actin
2.5
2.0
1.5
1.0
0.5
0.0
APP
CTFβ/α
IDE
ECE-1
J
$App^{NL-F/NL-F}$
Molecular layer
$App^{NL-F/NL-F}$/ Ensa KO
Molecular layer
K
$App^{NL-F/NL-F}$
$App^{NL-F/NL-F}$ / Ensa KO
(%)
Area of Amyloid β (N3pE)
0.6
0.4
0.2
0.0
**

Supplementary Figure S3. Generation of ENSA-deficient mouse using CRISPR/Cas9.

A. Schematic image for CRISPR/Cas9-mediated ENSA deficiency. B. Sanger sequence chromatograms near exon 1 of *Ensa* gene in WT and *Ensa* KO mice. Arrowheads show Cas9 cleavage sites. C. PCR-based genotyping results of WT, heterozygous and homozygous *Ensa* KO mice. Genotyping was performed using mouse tail genome. D. Immunoblotting of ENSA in WT and *Ensa* KO mice. E. Immunostaining of ENSA in WT and *Ensa* KO mice. ENSA immunoreactivity is absent in *Ensa* KO mice. Scale bar = 500 µm. Inset Scale bar = 50µm. F. PCR-based genotyping results of off-target sites in WT and founder *Ensa* KO mice. Genotyping was performed using mouse tail genome. G. Sanger sequence chromatograms of off-target sites in WT, founder mouse and F2 *Ensa* KO mouse. H. Immunoblotting of APP, CTFs, IDE and ECE-1 in 3-month-old WT and *Ensa* KO mice. I. Values indicated in graphs show band intensities for APP, CTFs, IDE and ECE-1 normalized to that of β-actin (n = 4 for each group). J and K. Immunostaining of $A\beta_{N3(pE)}$ (Green), NEP (Red) and DAPI (blue) from 18-month-old $App^{NL\text{-}F}$ and $App^{NL\text{-}F}$/*Ensa* KO mice. Statistical analysis of amyloid $\beta_{N3(pE)}$-positive area in 18-month-old $App^{NL\text{-}F}$ and $App^{NL\text{-}F}$/*Ensa* KO mice (n = 6 for each group). Scale bar is 100 µm. Results are expressed as the mean ±SEM. ***P*<0.01 (Student's *t*-test).

fig. S4

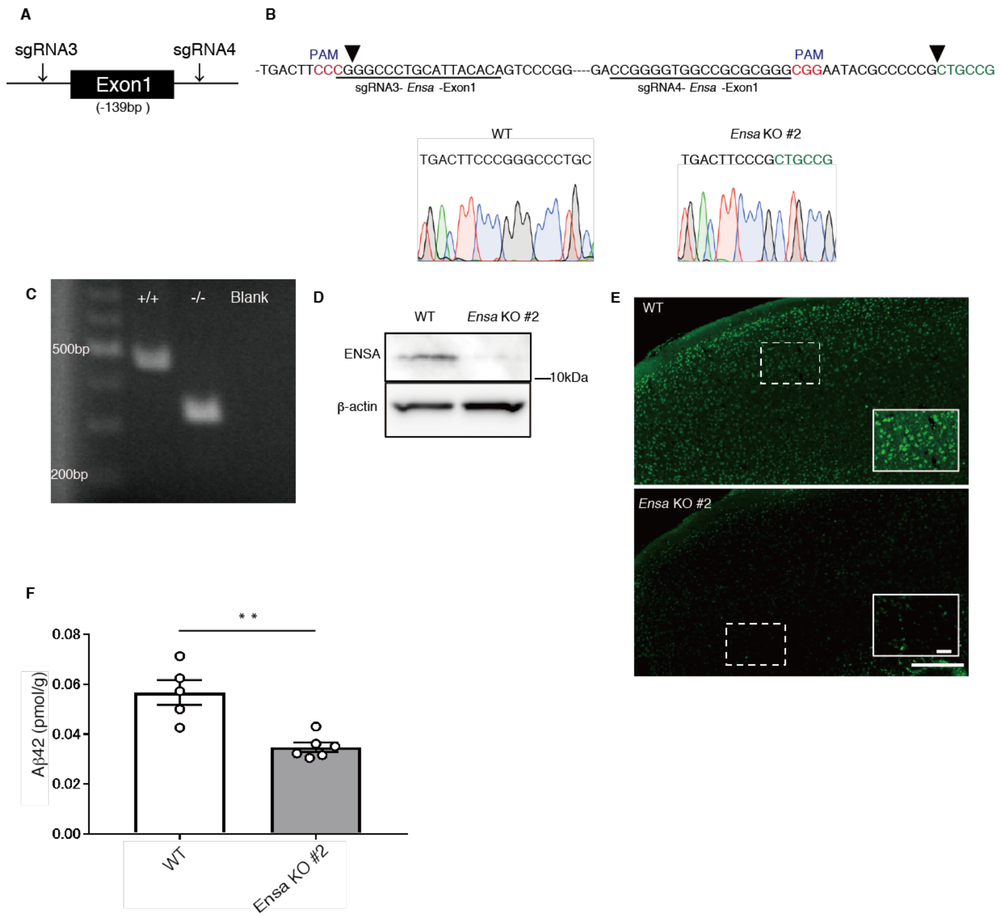

Supplementary Figure S4. Generation of 2nd line ENSA-deficient mouse using CRISPR/Cas9.

A. Schematic image for CRISPR/Cas9-mediated ENSA deficiency. B. Sanger sequence chromatograms near exon 1 of *Ensa* gene in WT and *Ensa* KO #2 mice. Arrowheads show cleavage sites by Cas9. C. PCR-based genotyping results of WT and *Ensa* KO #2 mice. Genotyping was performed using mouse tail genome. D. Immunoblotting of ENSA in WT and *Ensa* KO #2 mice. E. Immunostaining of ENSA in WT and *Ensa* KO #2 mice. ENSA immunoreactivity was absent in *Ensa* KO #2 mice. Scale bar = 500 µm. Inset scale bar = 50 µm. F. Aβ_{42} ELISA of hippocampi from 3-month-old WT and *Ensa* KO #2 mice (WT: n = 5, *Ensa* KO #2: n = 6). Results are expressed as the mean ±SEM. ***P*<0.01 (Student's *t*-test).

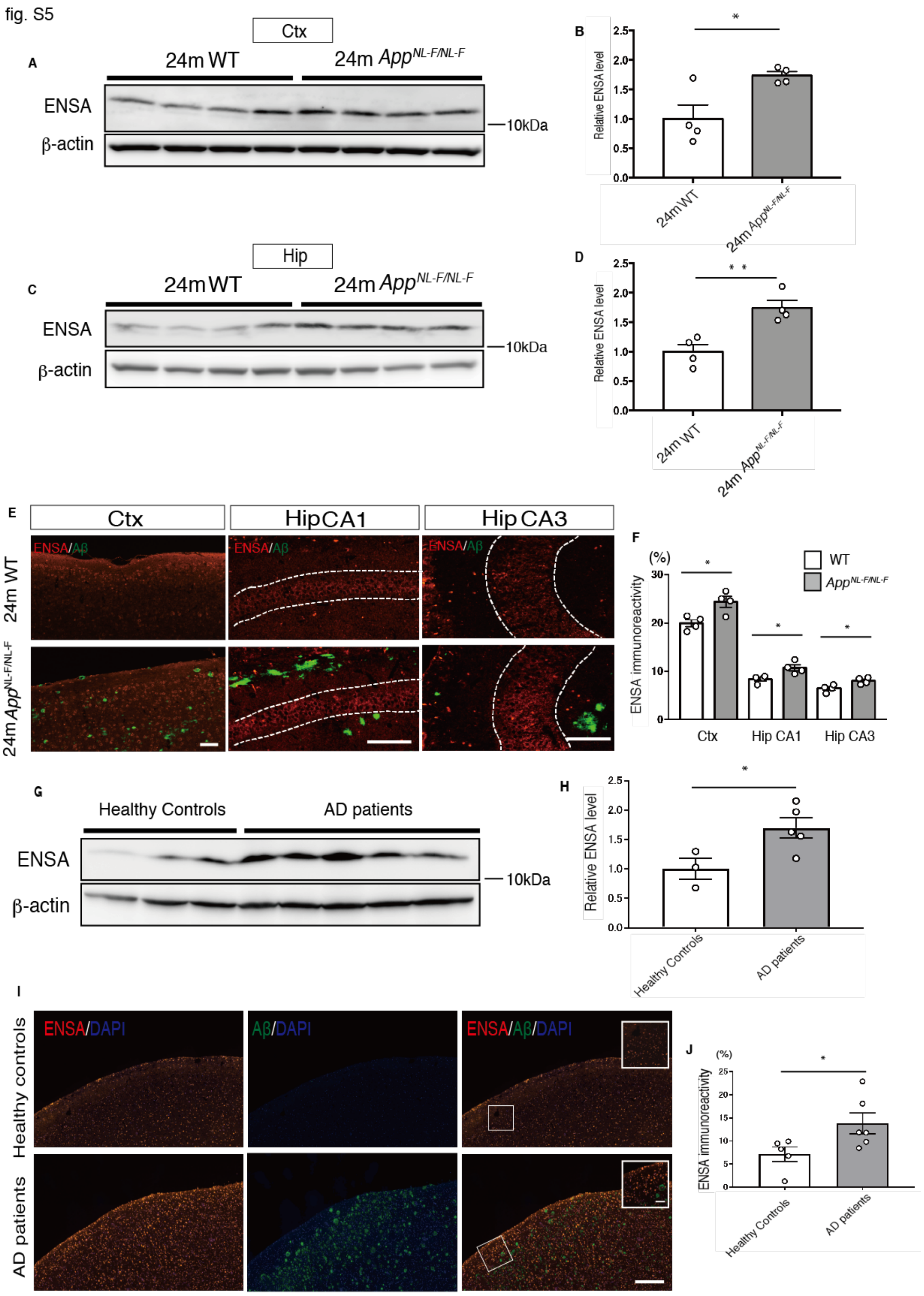

46

47 **Supplementary Figure S5. Increased levels of ENSA in AD model mouse and postmortem brain tissue**

from patients with AD. A-D. Immunoblotting of ENSA in (A and B) cortices and (C and D) hippocampi of 24-month-old WT and $App^{NL\text{-}F}$ mice. Values indicated in the graph show ENSA band intensities normalized to that of β-actin (n = 4 for each group). E and F. Immunostaining of ENSA (Red) and Aβ (Green) in cortex, and hippocampal CA1 and CA3 regions of 24-month-old WT and $App^{NL\text{-}F}$ mice (n = 4 for each group). Scale bar is 100 μm. G and H. Immunoblotting of ENSA in cortices of healthy controls and AD patients. Values indicated in the graph show ENSA band intensities normalized to that of β-actin (healthy controls: n = 3, AD patients: n = 5). I and J. Immunostaining of ENSA in cortices of healthy controls and AD patients (healthy controls: n = 5, AD patients: n = 6). Scale bar is 500 μm in low-magnification image and 100 μm in high-magnification image. Data represent the mean ±SEM. *P<0.05, **P<0.01, (Student's t-test). Information concerning human samples is given in Supplementary Table S9.

fig.S6

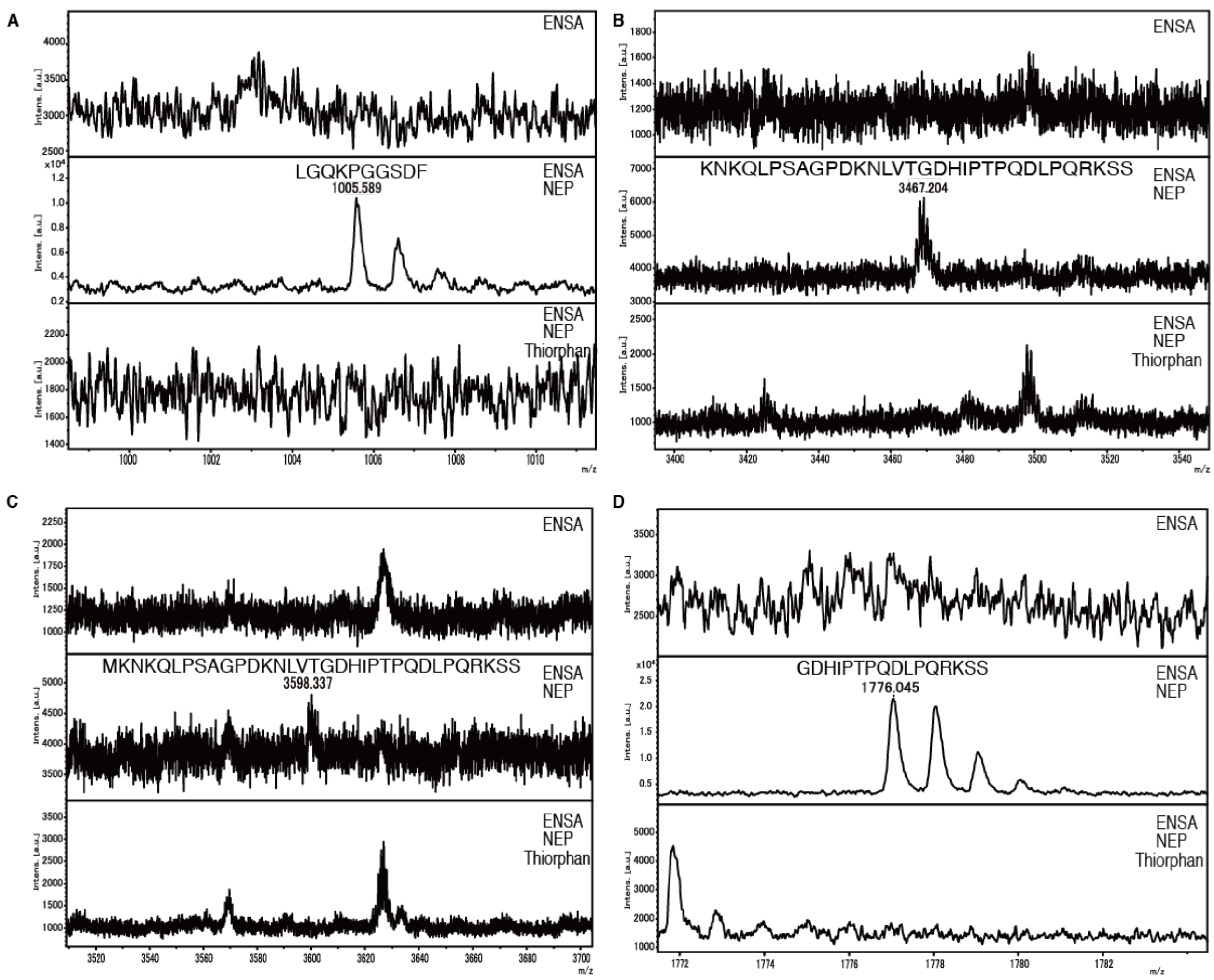

Supplementary Figure S6. Specific peaks of ENSA cleaved by NEP.

A-D. MALDI-TOF analyses showing specific peaks of cleaved ENSA after incubation in the presence or absence of NEP and thiorphan for 24 hours at 37˚C. LC-MS/MS analysis was used to determine specific amino acid sequences (Supplementary Table S8).

fig. S7

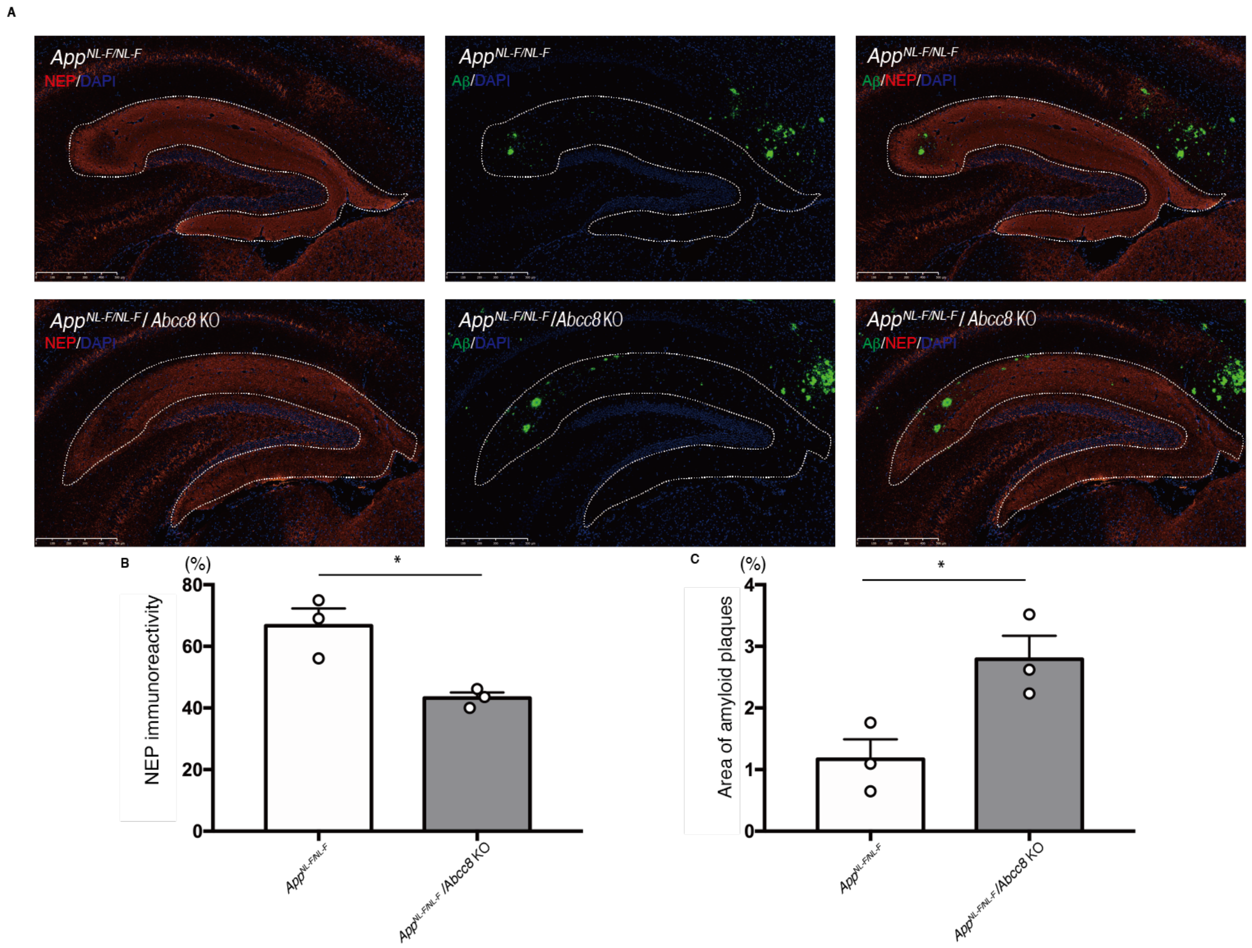

Supplementary Figure S7. Increase of amyloid deposition in *App*$^{NL\text{-}F}$/*Abcc8* KO mice.

A. Immunostaining of NEP (Red), Aβ (Green) and DAPI (blue) from 12-month-old *App*$^{NL\text{-}F}$ and *App*$^{NL\text{-}F}$/*Abcc8* KO mice. B and C. Statistical analysis of NEP and amyloid β positive signals in 12-month-old *App*$^{NL\text{-}F}$ and *App*$^{NL\text{-}F}$/*Abcc8* KO mice (n = 3 for each group). Scale bar is 500 µm. Results are expressed as the mean ±SEM. *P<0.05 (Student's *t*-test).

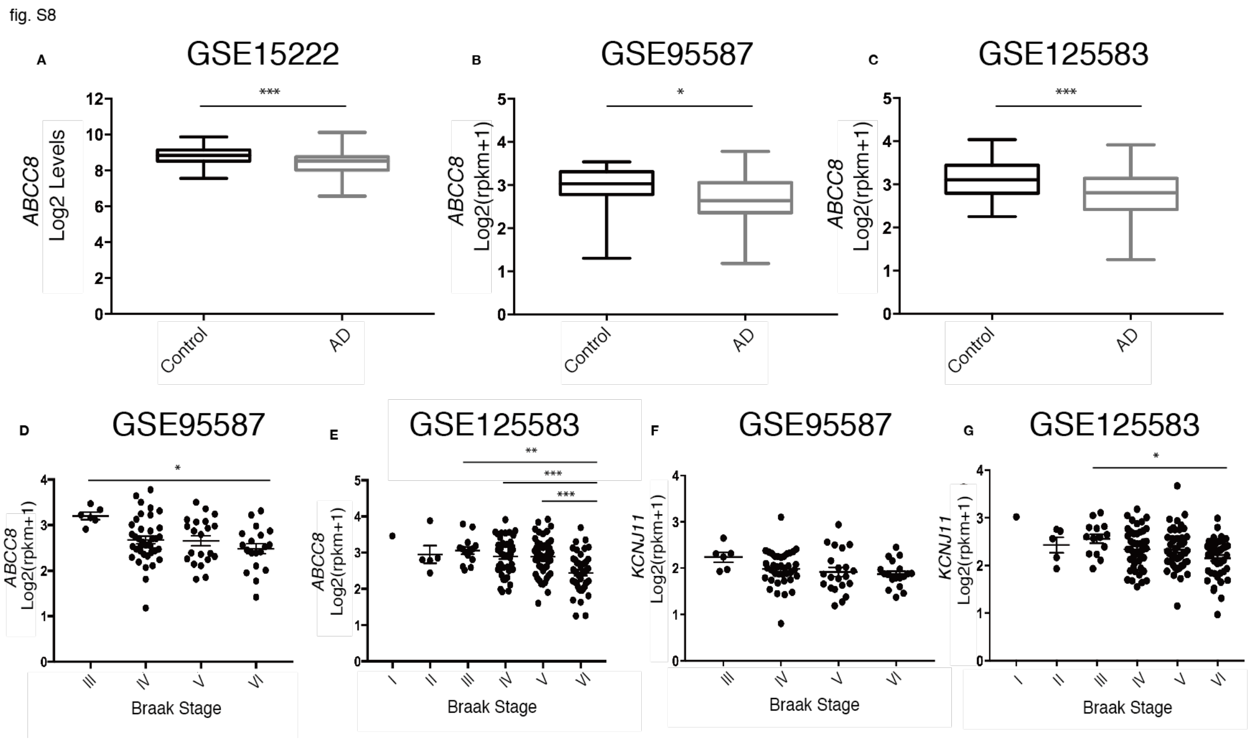

Supplementary Figure S8. Gene expression of K_{ATP} channel components in AD patients.

A-C. Gene expression of *ABCC8* mRNA in healthy controls and AD patients in the GSE15222, GSE95587 and GSE125583 cohorts. D-G. Gene expression of *ABCC8* and *KCNJ11* mRNA levels with differentiating Braak stage in the GSE95587 and GSE125583 cohorts. Statistical data are summarized in Supplementary Table S10 and S11.

fig. S9

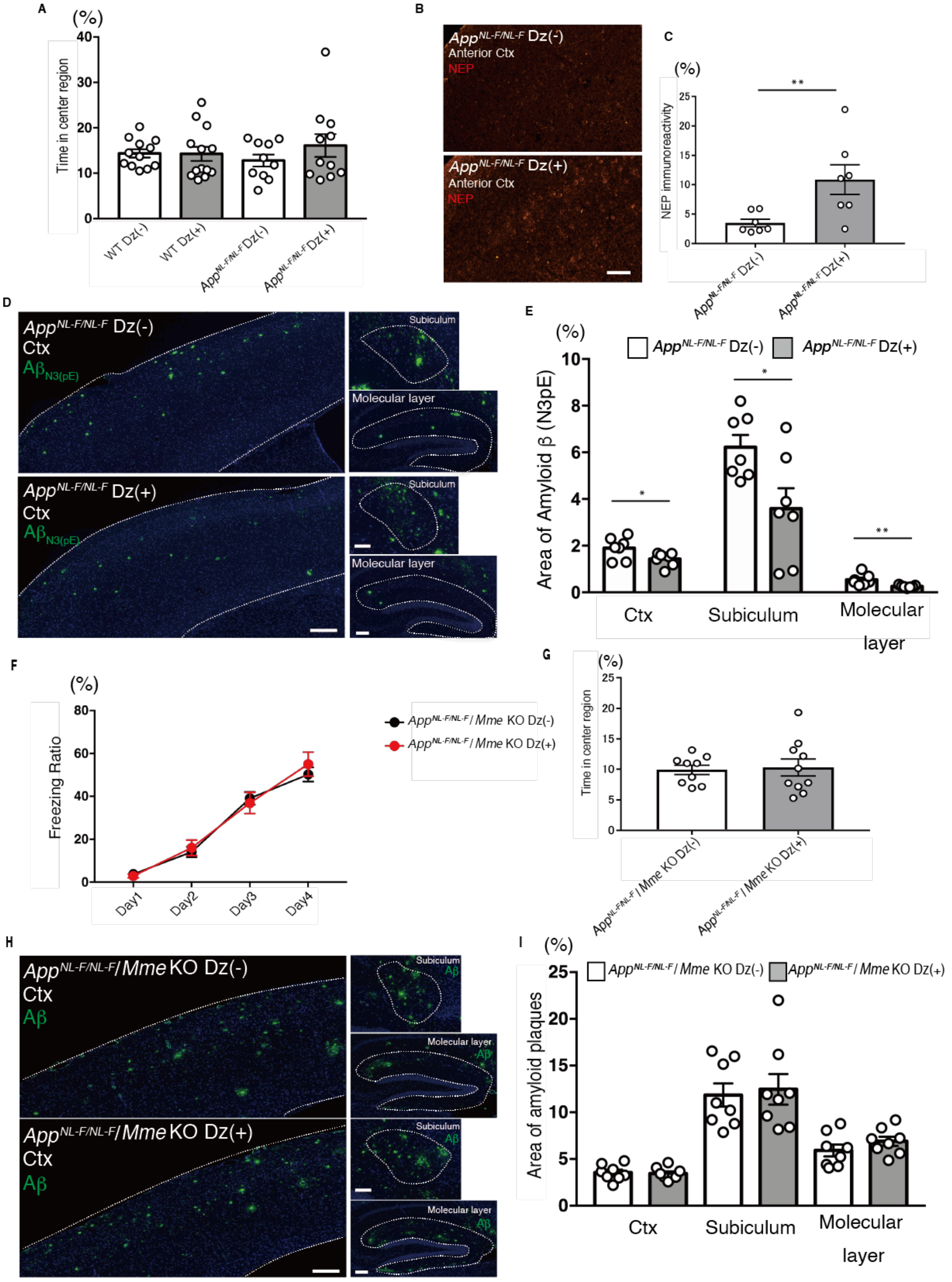

Supplementary Figure S9. Effect of Dz in *App*$^{NL-F}$ and *App*$^{NL-F}$/*Mme* KO mice.

A. Statistical analysis of open field test to measure time in central region. 18-month-old WT and *App*$^{NL-F}$ mice were treated with or without Dz for 3 months (WT Dz (-): n = 12, WT Dz (+): n = 13, *App*$^{NL-F}$ Dz (-): n = 10,

App^{NL-F} Dz (+): n = 11). B and C. Immunostaining of NEP in cortices of 18-month-old *App*$^{NL-F}$ mice treated with or without Dz for 3 months (n = 7 for each group). Scale bar = 500 µm. D. Immunostaining of A$\beta_{N3(pE)}$ (Green) in cortex, subiculum and molecular layer of 18-month-old *App*$^{NL-F}$ mice with or without Dz for 3 months. Scale bar in cortical image = 500 µm and in hippocampal image = 200µm. E. Statistical analysis of amyloid $\beta_{N3(pE)}$ positive area in 18-month-old *App*$^{NL-F}$ treated with or without Dz for 3 months (n = 7 for each group). F. Freezing ratio of 15-month-old *App*$^{NL-F}$/*Mme* KO mice treated with or without Dz for 3 months (*App*$^{NL-F}$/*Mme* KO Dz (-): n = 9, *App*$^{NL-F}$/*Mme* KO Dz (+): n = 10). G. Statistical analysis of open field test to measure time in central region of maze. 15-month-old *App*$^{NL-F}$/*Mme* KO were treated with or without Dz for 3 months (*App*$^{NL-F}$/*Mme* KO Dz (-): n = 9, *App*$^{NL-F}$/*Mme* KO Dz (+): n = 10). H. Immunostaining of Aβ (Green) in cortex, subiculum and molecular layer of 15-month-old *App*$^{NL-F}$/*Mme* KO mice with or without Dz for 3 months. Scale bar in cortical image = 500 µm and in hippocampal image = 200µm. I. Statistical analysis of amyloid plaque area in 15-month-old *App*$^{NL-F}$/*Mme* KO treated with or without Dz for 3 months (n = 8 for each group). In (C), the data represent the mean ±SEM. $^{**}P<0.01$ (Mann-Whitney test). In (E), the data represent the mean ±SEM. $^{*}P<0.05$ (Student's *t*-test).

Name	Catalog ID	WB	IHC
ENSA	Genetex GTX10493	1:2000	1:100
ENSA(human)	Cell signaling 11915 clone(D5Z1U)	1:1000	-
VGAT	Synaptic Systems 117G4	-	1:500
NEP	Leica Biosystems 56C6	1:1000	1:100
NEP	R&D systmes AF1126	1:500	-
Aβ	Saido et al. Neuron, 1995(N1D)	-	1:400
$A\beta_{N3(pE)}$	Saido et al. Neuron, 1995(N3(pE))	-	1:400
Aβ(human)	IBL 10323 clone(82E1)	-	1:500
APP	Chemicon 22C11	1:1000	-
APP-CTF	Sigma A8717	1:1000	-
β-actin	SIGMA #A5441	1:5000	-

Supplementary Table S1. Antibodies information.

The table shows the list of antibodies used in this study. Dilutions for Western blotting and immunostaining are described.

WT		Control				+SST				+TT232				
Accession	Description	score	coverage	peptide	PSMs	score	coverage	peptide	PSMs	score	coverage	peptide	PSMs	MW(kDa)
Q91XV3	Brain acid soluble protein 1 OS=Mus musculus GN=Basp1 PE=1 SV=3 - [BASP1_MOUSE]	604.47	72.12	10	16	231.84	57.96	8	8	382.12	58.41	9	11	22.1
P26645	Myristoylated alanine-rich C-kinase substrate OS=Mus musculus GN=Marcks PE=1 SV=2 - [MAI	356.46	36.25	6	7	51.49	5.83	1	1	133.72	11.00	2	3	29.6
P06837	Neuromodulin OS=Mus musculus GN=Gap43 PE=1 SV=1 - [NEUM_MOUSE]	259.68	33.04	8	11	37.55	13.66	2	2	121.71	18.06	4	5	23.6
Q9QWL7	Keratin, type I cytoskeletal 17 OS=Mus musculus GN=Krt17 PE=1 SV=3 - [K1C17_MOUSE]	447.27	24.02	14	18	423.10	21.02	11	15	234.34	9.47	5	7	48.1
Q6IFX2	Keratin, type I cytoskeletal 42 OS=Mus musculus GN=Krt42 PE=1 SV=1 - [K1C42_MOUSE]	378.20	21.90	11	14	468.64	21.68	10	15	168.93	15.49	7	7	50.1
P20065	Thymosin beta-4 OS=Mus musculus GN=Tmsb4x PE=1 SV=1 - [TYB4_MOUSE]	31.72	24.00	1	1									5.7
Q922U2	Keratin, type II cytoskeletal 5 OS=Mus musculus GN=Krt5 PE=1 SV=1 - [K2C5_MOUSE]	537.27	20.17	17	25	375.43	14.66	12	16	211.91	14.66	13	14	61.7
Q9Z2K1	Keratin, type I cytoskeletal 16 OS=Mus musculus GN=Krt16 PE=1 SV=3 - [K1C16_MOUSE]	288.16	16.63	8	10	216.31	10.45	7	9	112.22	11.94	7	7	51.6
P02798	Metallothionein-2 OS=Mus musculus GN=Mt2 PE=1 SV=2 - [MT2_MOUSE]	48.67	19.67	1	1	44.46	19.67	1	1		0.00			6.1
P02802	Metallothionein-1 OS=Mus musculus GN=Mt1 PE=1 SV=1 - [MT1_MOUSE]					52.93	19.67	1	1	62.32	19.67	1	1	6.0
P50446	Keratin, type II cytoskeletal 6A OS=Mus musculus GN=Krt6a PE=1 SV=3 - [K2C6A_MOUSE]	496.88	19.17	15	20	288.12	9.40	6	10	147.01	10.13	8	8	59.3
P28184	Metallothionein-3 OS=Mus musculus GN=Mt3 PE=1 SV=1 - [MT3_MOUSE]	60.08	17.65	1	1		0.00			59.93	17.65	1	1	7.0
Q9CQH7	Transcription factor BTF3 homolog 4 OS=Mus musculus GN=Btf3l4 PE=2 SV=1 - [BT3L4_MOUS	24.10	16.46	1	1									17.3
P08228	Superoxide dismutase [Cu-Zn] OS=Mus musculus GN=Sod1 PE=1 SV=2 - [SODC_MOUSE]	77.10	15.58	2	2	40.26	8.44	1	1	59.57	8.44	1	1	15.9
Q64387	Prepronociceptin OS=Mus musculus GN=Pnoc PE=2 SV=1 - [PNOC_MOUSE]	33.65	14.44	1	1					24.44	14.44	1	1	20.9
P07309	Transthyretin OS=Mus musculus GN=Ttr PE=1 SV=1 - [TTHY_MOUSE]	20.04	10.20	1	1		0.00			34.18	4.08	1	1	15.8
P26350	Prothymosin alpha OS=Mus musculus GN=Ptma PE=1 SV=2 - [PTMA_MOUSE]	303.03	13.51	3	4	175.44	12.61	2	2	79.55	12.61	2	2	12.2
P02535	Keratin, type I cytoskeletal 10 OS=Mus musculus GN=Krt10 PE=1 SV=3 - [K1C10_MOUSE]	378.56	10.53	7	11	347.81	10.88	7	10	272.76	12.11	8	10	57.7
P60710	Actin, cytoplasmic 1 OS=Mus musculus GN=Actb PE=1 SV=1 - [ACTB_MOUSE]	39.55	2.67	1	1	32.89	8.00	1	1	54.31	1.60	1	1	41.7
P70663	SPARC-like protein 1 OS=Mus musculus GN=Sparcl1 PE=2 SV=3 - [SPRL1_MOUSE]	155.95	12.15	6	6		0.00			77.66	3.23	1	1	72.2
Q9D0J8	Parathymosin OS=Mus musculus GN=Ptms PE=1 SV=3 - [PTMS_MOUSE]	66.86	11.88	1	2	27.56	11.88	1	1	32.69	11.88	1	1	11.4
P0CG49	Polyubiquitin-B OS=Mus musculus GN=Ubb PE=2 SV=1 - [UBB_MOUSE]					0.00	11.80	1	1					34.3
P60840	Alpha-endosulfine OS=Mus musculus GN=Ensa PE=1 SV=1 - [ENSA_MOUSE]	19.98	11.57	1	1		0.00							13.3
P26339	Chromogranin-A OS=Mus musculus GN=Chga PE=1 SV=1 - [CMGA_MOUSE]	192.67	9.72	5	6	36.09	2.38	1	1	115.24	5.83	3	3	51.8
Q6IFZ6	Keratin, type II cytoskeletal 1b OS=Mus musculus GN=Krt77 PE=1 SV=1 - [K2C1B_MOUSE]	219.79	5.42	6	10	350.42	9.62	7	11	106.97	5.42	5	5	61.3
P60041	Somatostatin OS=Mus musculus GN=Sst PE=3 SV=1 - [SMS_MOUSE]									61.79	9.48	1	1	12.7
Q8VED5	Keratin, type II cytoskeletal 79 OS=Mus musculus GN=Krt79 PE=2 SV=2 - [K2C79_MOUSE]	176.08	9.42	9	10	176.51	8.10	6	7	120.99	7.16	6	6	57.5
Q6NXH9	Keratin, type II cytoskeletal 73 OS=Mus musculus GN=Krt73 PE=1 SV=1 - [K2C73_MOUSE]	315.22	9.28	6	10	298.41	5.94	3	8	177.53	9.28	6	7	58.9
Q6P8I4	PEST proteolytic signal-containing nuclear protein OS=Mus musculus GN=Pcnp PE=1 SV=1 - [F	28.53	8.99	1	1									19.0
Q02257	Junction plakoglobin OS=Mus musculus GN=Jup PE=1 SV=3 - [PLAK_MOUSE]	73.17	6.17	4	4	85.59	4.03	2	2	27.39	2.42	1	1	81.7
P10639	Thioredoxin OS=Mus musculus GN=Txn PE=1 SV=3 - [THIO_MOUSE]					65.29	8.57	1	1					11.7
P17897	Lysozyme C-1 OS=Mus musculus GN=Lyz1 PE=1 SV=1 - [LYZ1_MOUSE]					73.64	8.11	1	1					16.8
P54227	Stathmin OS=Mus musculus GN=Stmn1 PE=1 SV=2 - [STMN1_MOUSE]	24.90	8.05	1	1						0.00			17.3
Q2YFS2	Paired immunoglobulin-like type 2 receptor beta OS=Mus musculus GN=Pilrb PE=1 SV=1 - [PILRB_MOUSE]					13.93	7.59	1	1					25.2
Q3UV17	Keratin, type II cytoskeletal 2 oral OS=Mus musculus GN=Krt76 PE=2 SV=1 - [K22O_MOUSE]	146.24	7.41	6	6	79.34	3.03	2	4	95.76	7.58	7	7	62.8
Q8CCT4	Transcription elongation factor A protein-like 5 OS=Mus musculus GN=Tceal5 PE=2 SV=1 - [TC	21.23	7.00	1	1									22.0
Q03517	Secretogranin-2 OS=Mus musculus GN=Scg2 PE=1 SV=1 - [SCG2_MOUSE]	88.34	6.97	4	4					59.25	3.40	2	2	70.6
Q3TTY5	Keratin, type II cytoskeletal 2 epidermal OS=Mus musculus GN=Krt2 PE=1 SV=1 - [K22E_MOU	197.40	6.65	9	10	164.08	5.37	6	8	131.74	6.65	8	9	70.9
P28667	MARCKS-related protein OS=Mus musculus GN=Marcksl1 PE=1 SV=2 - [MRP_MOUSE]	18.69	6.50	1	1		0.00				0.00			20.2
P01325	Insulin-1 OS=Mus musculus GN=Ins1 PE=1 SV=1 - [INS1_MOUSE]		0.00				0.00			19.76	6.48	1	1	12.2
P04104	Keratin, type II cytoskeletal 1 OS=Mus musculus GN=Krt1 PE=1 SV=4 - [K2C1_MOUSE]	409.24	6.44	8	15	561.17	6.44	7	16	252.49	6.44	7	9	65.6
Q62092	Neuron-specific protein family member 1 OS=Mus musculus GN=Nsg1 PE=2 SV=3 - [NSG1_MC	55.54	5.95	1	1									20.9
Q6IME9	Keratin, type II cytoskeletal 72 OS=Mus musculus GN=Krt72 PE=3 SV=1 - [K2C72_MOUSE]	102.92	5.77	4	6	53.09	1.73	1	2	73.38	4.04	3	3	56.7
P17742	Peptidyl-prolyl cis-trans isomerase A OS=Mus musculus GN=Ppia PE=1 SV=2 - [PPIA_MOUSE]	23.63	5.49	1	1					28.97	5.49	1	1	18.0
Q8C854	Myelin expression factor 2 OS=Mus musculus GN=Myef2 PE=1 SV=1 - [MYEF2_MOUSE]	131.79	5.25	2	2		0.00			77.58	5.25	2	2	63.3
Q80XU3	Nuclear ubiquitous casein and cyclin-dependent kinase substrate 1 OS=Mus musculus GN=Nuc	60.60	5.13	1	1									26.3
Q62446	Peptidyl-prolyl cis-trans isomerase FKBP3 OS=Mus musculus GN=Fkbp3 PE=1 SV=2 - [FKBP3_I	23.66	4.91	1	1									25.1
Q9QXV0	ProSAAS OS=Mus musculus GN=Pcsk1n PE=1 SV=2 - [PCSK1_MOUSE]	44.67	4.65	1	1		0.00			43.12	4.65	1	1	27.3
P07724	Serum albumin OS=Mus musculus GN=Alb PE=1 SV=3 - [ALBU_MOUSE]	735.53	4.44	2	20	344.25	4.44	2	10	819.12	4.44	2	24	68.6
P16014	Secretogranin-1 OS=Mus musculus GN=Chgb PE=1 SV=2 - [SCG1_MOUSE]	125.24	4.43	2	2		0.00			72.58	4.43	2	2	77.9
P47867	Secretogranin-3 OS=Mus musculus GN=Scg3 PE=1 SV=1 - [SCG3_MOUSE]	23.88	4.03	2	2									53.3
P35700	Peroxiredoxin-1 OS=Mus musculus GN=Prdx1 PE=1 SV=1 - [PRDX1_MOUSE]					38.52	4.02	1	1					22.2
P17182	Alpha-enolase OS=Mus musculus GN=Eno1 PE=1 SV=3 - [ENOA_MOUSE]					0.00	3.92	2	2					47.1
P46737	Lys-63-specific deubiquitinase BRCC36 OS=Mus musculus GN=Brcc3 PE=2 SV=1 - [BRCC3_MO	24.85	3.44	1	1		0.00							33.3
P22005	Proenkephalin-A OS=Mus musculus GN=Penk PE=2 SV=2 - [PENK_MOUSE]	36.02	3.36	1	1	31.60	3.36	1	1	28.55	3.36	1	1	31.0
E9Q557	Desmoplakin OS=Mus musculus GN=Dsp PE=1 SV=1 - [DESP_MOUSE]	100.83	0.94	3	3	183.37	2.71	7	7		0.00			332.7
Q8BLK3	Limbic system-associated membrane protein OS=Mus musculus GN=Lsamp PE=1 SV=1 - [LSAI	36.84	3.23	1	1									38.1
P20152	Vimentin OS=Mus musculus GN=Vim PE=1 SV=3 - [VIME_MOUSE]	37.76	3.22	2	2		0.00			24.29	1.50	1	1	53.7
P07356	Annexin A2 OS=Mus musculus GN=Anxa2 PE=1 SV=2 - [ANXA2_MOUSE]					26.60	2.95	1	1					38.7
P30681	High mobility group protein B2 OS=Mus musculus GN=Hmgb2 PE=1 SV=3 - [HMGB2_MOUSE]	30.54	2.86	1	1					13.62	2.86	1	1	24.1
Q99L43	Phosphatidate cytidylyltransferase 2 OS=Mus musculus GN=Cds2 PE=1 SV=1 - [CDS2_MOUSE]									41.62	2.70	1	1	51.3
Q9R0P9	Ubiquitin carboxyl-terminal hydrolase isozyme L1 OS=Mus musculus GN=Uchl1 PE=1 SV=1 - [U	28.81	2.69	1	1						0.00			24.8
P16858	Glyceraldehyde-3-phosphate dehydrogenase OS=Mus musculus GN=Gapdh PE=1 SV=2 - [G3P_MOUSE]		0.00			23.45	2.40	1	1		0.00			35.8
P61979	Heterogeneous nuclear ribonucleoprotein K OS=Mus musculus GN=Hnrnpk PE=1 SV=1 - [HNR	38.12	2.38	1	1									50.9
P13595	Neural cell adhesion molecule 1 OS=Mus musculus GN=Ncam1 PE=1 SV=3 - [NCAM1_MOUSE]	77.58	2.24	3	3	43.85	1.26	1	1	42.97	1.26	1	1	119.4
P11499	Heat shock protein HSP 90-beta OS=Mus musculus GN=Hsp90ab1 PE=1 SV=3 - [HS90B_MOUS	52.00	2.21	1	1									83.2
Q7TQF7	Amphiphysin OS=Mus musculus GN=Amph PE=1 SV=1 - [AMPH_MOUSE]	31.49	2.19	1	1									75.0
Q9D518	Protein FAM227B OS=Mus musculus GN=Fam227b PE=2 SV=3 - [F227B_MOUSE]	16.57	2.07	1	3					0.00	2.07	1	1	62.7
Q00PI9	Heterogeneous nuclear ribonucleoprotein U-like protein 2 OS=Mus musculus GN=Hnrnpul2 PE=	52.24	1.88	1	1						0.00			84.9
Q9CPT0	Apoptosis facilitator Bcl-2-like protein 14 OS=Mus musculus GN=Bcl2l14 PE=1 SV=1 - [B2L14_MOUSE]									27.59	1.83	1	1	37.0
P99024	Tubulin beta-5 chain OS=Mus musculus GN=Tubb5 PE=1 SV=1 - [TBB5_MOUSE]	26.58	1.80	1	1									49.6
P68368	Tubulin alpha-4A chain OS=Mus musculus GN=Tuba4a PE=1 SV=1 - [TBA4A_MOUSE]	36.44	1.79	1	2	18.91	1.79	1	1	26.87	1.79	1	2	49.9
Q61316	Heat shock 70 kDa protein 4 OS=Mus musculus GN=Hspa4 PE=1 SV=1 - [HSP74_MOUSE]	60.05	1.66	1	1									94.1
Q7TSG5	SH3 domain-containing protein 21 OS=Mus musculus GN=Sh3d21 PE=2 SV=1 - [SH321_MOUS	83.21	1.64	1	7	26.13	1.64	1	1	286.16	1.64	1	18	60.3
Q7TPC1	Corneodesmosin OS=Mus musculus GN=Cdsn PE=2 SV=2 - [CDSN_MOUSE]	28.45	1.60	1	1		0.00				0.00			54.3
P35396	Peroxisome proliferator-activated receptor delta OS=Mus musculus GN=Ppard PE=2 SV=1 - [PPARD_MOU		0.00				0.00			21.71	1.59	1	1	49.7
Q7TQK5	Coiled-coil domain-containing protein 93 OS=Mus musculus GN=Ccdc93 PE=2 SV=1 - [CCD93_MOUSE]									17.50	1.59	1	1	72.6
P12023	Amyloid beta A4 protein OS=Mus musculus GN=App PE=1 SV=3 - [A4_MOUSE]	60.64	1.56	1	1	64.68	1.56	1	1	38.70	1.56	1	1	86.7
B9EKR1	Receptor-type tyrosine-protein phosphatase zeta OS=Mus musculus GN=Ptprz1 PE=1 SV=1 - [	133.65	1.34	2	2	53.02	0.52	1	1	61.17	0.52	1	1	254.2
Q9EPL2	Calsyntenin-1 OS=Mus musculus GN=Clstn1 PE=1 SV=1 - [CSTN1_MOUSE]	23.86	1.33	1	1					25.73	1.33	1	1	108.8
Q61147	Ceruloplasmin OS=Mus musculus GN=Cp PE=1 SV=2 - [CERU_MOUSE]	19.22	1.23	2	2									121.1
Q61495	Desmoglein-1-alpha OS=Mus musculus GN=Dsg1a PE=2 SV=2 - [DSG1A_MOUSE]	25.67	1.23	1	1		0.00							114.5
Q8C3X4	Translation factor Guf1, mitochondrial OS=Mus musculus GN=Guf1 PE=2 SV=1 - [GUF1_MOUS	0.00	1.08	1	1		0.00				0.00			72.4
P08113	Endoplasmin OS=Mus musculus GN=Hsp90b1 PE=1 SV=2 - [ENPL_MOUSE]	42.74	1.00	1	1		0.00			18.31	1.00	1	1	92.4
Q99P72	Reticulon-4 OS=Mus musculus GN=Rtn4 PE=1 SV=2 - [RTN4_MOUSE]	22.41	0.86	1	1									126.5
P20357	Microtubule-associated protein 2 OS=Mus musculus GN=Map2 PE=1 SV=2 - [MTAP2_MOUSE]	19.13	0.66	1	1									199.0
Q8CGK3	Lon protease homolog, mitochondrial OS=Mus musculus GN=Lonp1 PE=1 SV=2 - [LONM_MOU	0.00	0.63	1	1									105.8
P01027	Complement C3 OS=Mus musculus GN=C3 PE=1 SV=3 - [CO3_MOUSE]	21.28	0.60	1	1					24.27	0.60	1	1	186.4
Q9WTL4	Insulin receptor-related protein OS=Mus musculus GN=Insrr PE=1 SV=2 - [INSRR_MOUSE]	0.00	0.54	1	1									144.8
P11627	Neural cell adhesion molecule L1 OS=Mus musculus GN=L1cam PE=1 SV=1 - [L1CAM_MOUSE]		0.00							30.68	0.48	1	1	140.9
Q9CWL2	Zinc finger protein castor homolog 1 OS=Mus musculus GN=Casz1 PE=2 SV=3 - [CASZ1_MOUSE]									0.00	0.40	1	1	191.1
Q811D2	Ankyrin repeat domain-containing protein 26 OS=Mus musculus GN=Ankrd26 PE=1 SV=2 - [AN	27.70	0.38	1	1		0.00				0.00			180.5
Q60841	Reelin OS=Mus musculus GN=Reln PE=1 SV=3 - [RELN_MOUSE]	33.47	0.29	1	1									387.2
Q9QYX7	Protein piccolo OS=Mus musculus GN=Pclo PE=1 SV=4 - [PCLO_MOUSE]		0.00				0.00			19.47	0.18	1	1	550.5

Supplementary Table S2. The list of proteins identified in LC-MS/MS analysis of conditioned media from WT neuron.

Candidate proteins regulating NEP activity were screened by the absence or presence only in the media of the SST- and TT232-treated WT neurons in the range of 10 to 30 kDa molecular weight, but not in the media of *Sst*$_1$/*Sst*$_4$ dKO neurons (Supplementary Table S3). Red: Control samples, Blue: SST-treated samples, Green:

TT232-treated samples. ENSA, NSG1 and NUCKS1 were highlighted in yellow.

Sst_1/Sst_4 dKO		Control				+SST				+TT232				
Accession	Description	score	coverage	peptide	PSMs	score	coverage	peptide	PSMs	score	coverage	peptide	PSMs	MW(kDa)
Q91XV3	Brain acid soluble protein 1 OS=Mus musculus GN=Basp1 PE=1 SV=3 - [BASP1_MOUSE]	216.17	46.02	5	6	253.04	51.77	6	8	237.87	46.02	5	6	22.1
P08228	Superoxide dismutase [Cu-Zn] OS=Mus musculus GN=Sod1 PE=1 SV=2 - [SODC_MOUSE]	122.39	23.38	3	3	105.38	24.68	3	4	148.60	24.68	3	5	15.9
P06837	Neuromodulin OS=Mus musculus GN=Gap43 PE=1 SV=1 - [NEUM_MOUSE]	130.16	19.82	3	4	150.53	19.82	3	5	158.96	24.67	4	5	23.6
P02798	Metallothionein-2 OS=Mus musculus GN=Mt2 PE=1 SV=2 - [MT2_MOUSE]	24.08	19.67	1	1	48.88	19.67	1	1					6.1
P02802	Metallothionein-1 OS=Mus musculus GN=Mt1 PE=1 SV=1 - [MT1_MOUSE]	55.98	19.67	1	1	63.77	19.67	1	1	42.90	19.67	1	1	6.0
Q9QXV0	ProSAAS OS=Mus musculus GN=Pcsk1n PE=1 SV=2 - [PCSK1_MOUSE]	96.81	18.60	3	3	64.01	10.47	2	2	17.35	18.60	3	3	27.3
P28184	Metallothionein-3 OS=Mus musculus GN=Mt3 PE=1 SV=1 - [MT3_MOUSE]					48.20	17.65	1	1	59.43	17.65	1	1	7.0
Q9QWL7	Keratin, type I cytoskeletal 17 OS=Mus musculus GN=Krt17 PE=1 SV=3 - [K1C17_MOUSE]	175.37	8.78	5	6	189.10	13.63	6	6	231.72	15.01	7	8	48.1
Q9Z2K1	Keratin, type I cytoskeletal 16 OS=Mus musculus GN=Krt16 PE=1 SV=3 - [K1C16_MOUSE]	123.37	7.04	4	5	189.63	14.07	6	7	306.07	15.35	7	11	51.6
Q6IFX2	Keratin, type I cytoskeletal 42 OS=Mus musculus GN=Krt42 PE=1 SV=1 - [K1C42_MOUSE]	157.17	13.50	6	7	148.55	9.07	5	5	128.48	15.71	7	7	50.1
Q922U2	Keratin, type II cytoskeletal 5 OS=Mus musculus GN=Krt5 PE=1 SV=1 - [K2C5_MOUSE]	274.04	13.10	10	13	238.84	11.72	8	9	206.09	10.34	7	10	61.7
P70663	SPARC-like protein 1 OS=Mus musculus GN=Sparcl1 PE=2 SV=3 - [SPRL1_MOUSE]	111.28	10.00	4	4	116.05	10.15	4	4	165.05	15.38	7	8	72.2
P50446	Keratin, type II cytoskeletal 6A OS=Mus musculus GN=Krt6a PE=1 SV=3 - [K2C6A_MOUSE]	266.68	10.13	8	11	229.41	10.67	7	8	212.89	11.39	7	10	59.3
Q64387	Prepronociceptin OS=Mus musculus GN=Pnoc PE=2 SV=1 - [PNOC_MOUSE]	27.74	14.44	1	1	33.87	14.44	1	1	63.70	14.44	1	1	20.9
P26350	Prothymosin alpha OS=Mus musculus GN=Ptma PE=1 SV=2 - [PTMA_MOUSE]	182.72	13.51	2	4	257.78	12.61	2	3	285.13	13.51	3	6	12.2
P26339	Chromogranin-A OS=Mus musculus GN=Chga PE=1 SV=1 - [CMGA_MOUSE]	294.99	10.58	7	8	231.32	8.42	5	6	222.38	12.96	7	8	51.8
Q9D0J8	Parathymosin OS=Mus musculus GN=Ptms PE=1 SV=3 - [PTMS_MOUSE]	34.78	11.88	1	2	75.91	11.88	1	2	23.95	11.88	1	2	11.4
P02535	Keratin, type I cytoskeletal 10 OS=Mus musculus GN=Krt10 PE=1 SV=3 - [K1C10_MOUSE]	283.46	11.75	8	8	211.23	10.35	6	6	247.60	9.30	6	7	57.7
P60840	Alpha-endosulfine OS=Mus musculus GN=Ensa PE=1 SV=1 - [ENSA_MOUSE]	39.26	11.57	1	1	32.47	11.57	1	1	68.48	11.57	1	1	13.3
P21460	Cystatin-C OS=Mus musculus GN=Cst3 PE=2 SV=2 - [CYTC_MOUSE]									46.65	11.43	1	1	15.5
P26645	Myristoylated alanine-rich C-kinase substrate OS=Mus musculus GN=Marcks PE=1 SV=2 - [MARCS_MOUSE]	57.36	5.50	1	1	56.58	10.36	2	2	65.57	5.50	1	1	29.6
Q02257	Junction plakoglobin OS=Mus musculus GN=Jup PE=1 SV=3 - [PLAK_MOUSE]	20.22	3.36	2	2	13.46	1.61	1	1	81.78	6.44	4	4	81.7
P16014	Secretogranin-1 OS=Mus musculus GN=Chgb PE=1 SV=2 - [SCG1_MOUSE]	58.83	4.43	2	2	73.81	4.43	2	2	60.54	9.60	5	5	77.9
P60041	Somatostatin OS=Mus musculus GN=Sst PE=3 SV=1 - [SMS_MOUSE]	25.94	9.48	1	1	59.83	9.48	1	1	55.93	9.48	1	1	12.7
Q3TTY5	Keratin, type II cytoskeletal 2 epidermal OS=Mus musculus GN=Krt2 PE=1 SV=1 - [K22E_MOUSE]	217.27	8.63	9	10	191.84	8.49	8	9	232.60	7.36	7	9	70.9
P62264	40S ribosomal protein S14 OS=Mus musculus GN=Rps14 PE=2 SV=3 - [RS14_MOUSE]									16.41	8.61	1	1	16.3
P54227	Stathmin OS=Mus musculus GN=Stmn1 PE=1 SV=2 - [STMN1_MOUSE]	27.87	8.05	1	1									17.3
P0C0S6	Histone H2A.Z OS=Mus musculus GN=H2afz PE=1 SV=2 - [H2AZ_MOUSE]	26.65	7.81	1	1	24.52	7.81	1	1	16.76	7.81	1	1	13.5
Q8VED5	Keratin, type II cytoskeletal 79 OS=Mus musculus GN=Krt79 PE=2 SV=2 - [K2C79_MOUSE]	183.40	5.46	4	6	150.81	7.72	5	6	181.23	7.72	5	6	57.5
Q6NXH9	Keratin, type II cytoskeletal 73 OS=Mus musculus GN=Krt73 PE=1 SV=1 - [K2C73_MOUSE]	146.52	7.61	6	7	151.85	5.38	4	4	142.62	5.94	4	4	58.9
P35564	Calnexin OS=Mus musculus GN=Canx PE=1 SV=1 - [CALX_MOUSE]	27.32	3.38	1	1	44.87	2.03	1	2	96.47	6.94	3	4	67.2
P28667	MARCKS-related protein OS=Mus musculus GN=Marcksl1 PE=1 SV=2 - [MRP_MOUSE]	25.84	6.50	1	1		0.00			24.36	6.50	1	1	20.2
P01325	Insulin-1 OS=Mus musculus GN=Ins1 PE=1 SV=1 - [INS1_MOUSE]	25.20	6.48	1	1		0.00			17.43	6.48	1	1	12.2
P10923	Osteopontin OS=Mus musculus GN=Spp1 PE=1 SV=1 - [OSTP_MOUSE]									21.66	6.46	1	1	32.4
P04104	Keratin, type II cytoskeletal 1 OS=Mus musculus GN=Krt1 PE=1 SV=4 - [K2C1_MOUSE]	232.85	6.44	6	9	225.51	6.44	6	8	298.85	6.44	6	8	65.6
Q62092	Neuron-specific protein family member 1 OS=Mus musculus GN=Nsg1 PE=2 SV=3 - [NSG1_MOUSE]	87.12	5.95	1	1	67.68	5.95	1	1	54.36	5.95	1	1	20.9
P63101	14-3-3 protein zeta/delta OS=Mus musculus GN=Ywhaz PE=1 SV=1 - [1433Z_MOUSE]		0.00				0.00			17.36	5.71	1	1	27.8
P60710	Actin, cytoplasmic 1 OS=Mus musculus GN=Actb PE=1 SV=1 - [ACTB_MOUSE]	66.20	5.60	2	2	38.90	2.93	1	1	37.85	5.60	2	2	41.7
Q80XU3	Nuclear ubiquitous casein and cyclin-dependent kinase substrate 1 OS=Mus musculus GN=Nucks1 PE=1 SV=1 - [NU	34.69	5.13	1	1	43.73	5.56	2	2	64.60	5.56	2	4	26.3
Q61171	Peroxiredoxin-2 OS=Mus musculus GN=Prdx2 PE=1 SV=3 - [PRDX2_MOUSE]	23.43	5.56	1	1	38.31	5.56	1	1		0.00			21.8
Q8C854	Myelin expression factor 2 OS=Mus musculus GN=Myef2 PE=1 SV=1 - [MYEF2_MOUSE]	75.78	3.05	1	1	32.68	3.05	1	1	54.23	5.25	2	2	63.3
Q03517	Secretogranin-2 OS=Mus musculus GN=Scg2 PE=1 SV=1 - [SCG2_MOUSE]	75.24	3.08	2	2	56.70	3.57	2	2	88.90	3.08	2	2	70.6
Q9R1P4	Proteasome subunit alpha type-1 OS=Mus musculus GN=Psma1 PE=1 SV=1 - [PSA1_MOUSE]	25.24	4.94	1	1	51.34	4.94	1	1	19.27	4.94	1	1	29.5
Q62446	Peptidyl-prolyl cis-trans isomerase FKBP3 OS=Mus musculus GN=Fkbp3 PE=1 SV=2 - [FKBP3_MOUSE]	42.26	4.91	1	1									25.1
Q3UV17	Keratin, type II cytoskeletal 2 oral OS=Mus musculus GN=Krt76 PE=2 SV=1 - [K22O_MOUSE]	82.43	4.55	5	5	84.82	2.86	3	3	78.86	3.03	3	3	62.8
Q06335	Amyloid-like protein 2 OS=Mus musculus GN=Aplp2 PE=1 SV=4 - [APLP2_MOUSE]	63.72	4.53	2	2	40.66	4.53	2	2	63.34	4.53	2	2	80.4
P08226	Apolipoprotein E OS=Mus musculus GN=Apoe PE=1 SV=2 - [APOE_MOUSE]									27.76	4.50	1	1	35.8
P07724	Serum albumin OS=Mus musculus GN=Alb PE=1 SV=3 - [ALBU_MOUSE]	359.87	4.44	2	13	399.12	2.30	1	10	477.36	4.44	2	12	68.6
Q9DAR7	m7GpppX diphosphatase OS=Mus musculus GN=Dcps PE=1 SV=1 - [DCPS_MOUSE]									27.03	4.44	1	1	39.0
P07356	Annexin A2 OS=Mus musculus GN=Anxa2 PE=1 SV=2 - [ANXA2_MOUSE]									40.15	4.13	1	1	38.7
Q6IME9	Keratin, type II cytoskeletal 72 OS=Mus musculus GN=Krt72 PE=3 SV=1 - [K2C72_MOUSE]	52.32	1.73	2	2	95.84	4.04	3	3	94.63	4.04	3	4	56.7
P11499	Heat shock protein HSP 90-beta OS=Mus musculus GN=Hsp90ab1 PE=1 SV=3 - [HS90B_MOUSE]	79.39	3.87	2	2	143.38	2.21	2	2	137.39	4.01	3	4	83.2
Q61147	Ceruloplasmin OS=Mus musculus GN=Cp PE=1 SV=2 - [CERU_MOUSE]	48.45	0.94	1	1	73.90	2.26	2	2	84.63	2.45	3	4	121.1
P63017	Heat shock cognate 71 kDa protein OS=Mus musculus GN=Hspa8 PE=1 SV=1 - [HSP7C_MOUSE]		0.00			21.43	1.24	1	1	21.00	3.72	2	2	70.8
P49312	Heterogeneous nuclear ribonucleoprotein A1 OS=Mus musculus GN=Hnrnpa1 PE=1 SV=2 - [ROA1_MOUSE]	23.99	3.44	1	1	15.83	3.44	1	1	18.57	3.44	1	1	34.2
P12023	Amyloid beta A4 protein OS=Mus musculus GN=App PE=1 SV=3 - [A4_MOUSE]	42.57	1.56	1	2	32.30	1.56	1	1	86.87	3.25	2	2	86.7
Q99JF8	PC4 and SFRS1-interacting protein OS=Mus musculus GN=Psip1 PE=1 SV=1 - [PSIP1_MOUSE]					25.30	2.65	1	1	22.05	2.65	1	1	59.7
Q64299	Protein NOV homolog OS=Mus musculus GN=Nov PE=2 SV=1 - [NOV_MOUSE]									23.45	2.54	1	1	38.9
P16858	Glyceraldehyde-3-phosphate dehydrogenase OS=Mus musculus GN=Gapdh PE=1 SV=2 - [G3P_MOUSE]	17.28	2.40	1	1					19.81	2.40	1	1	35.8
P80560	Receptor-type tyrosine-protein phosphatase N2 OS=Mus musculus GN=Ptprn2 PE=1 SV=2 - [PTPR2_MOUSE]	16.65	2.10	1	1									111.4
Q9EPL2	Calsyntenin-1 OS=Mus musculus GN=Clstn1 PE=1 SV=1 - [CSTN1_MOUSE]	24.91	1.33	1	1		0.00			24.14	0.72	1	1	108.8
Q00PI9	Heterogeneous nuclear ribonucleoprotein U-like protein 2 OS=Mus musculus GN=Hnrnpul2 PE=1 SV=2 - [HNRL2_MO	44.04	1.88	1	1	42.72	1.88	1	1	44.78	1.88	1	1	84.9
E9Q557	Desmoplakin OS=Mus musculus GN=Dsp PE=1 SV=1 - [DESP_MOUSE]	42.65	0.52	1	1		0.00			57.43	1.35	5	5	332.7
Q03157	Amyloid-like protein 1 OS=Mus musculus GN=Aplp1 PE=1 SV=1 - [APLP1_MOUSE]	53.38	1.84	1	1	42.95	1.84	1	1	42.41	1.84	1	1	72.7
P20152	Vimentin OS=Mus musculus GN=Vim PE=1 SV=3 - [VIME_MOUSE]						0.00			41.35	1.72	1	1	53.7
Q61316	Heat shock 70 kDa protein 4 OS=Mus musculus GN=Hspa4 PE=1 SV=1 - [HSP74_MOUSE]									19.95	1.66	1	1	94.1
P35396	Peroxisome proliferator-activated receptor delta OS=Mus musculus GN=Ppard PE=2 SV=1 - [PPARD_MOUSE]	27.94	1.59	1	1	35.81	1.59	1	1	24.81	1.59	1	1	49.7
Q99MR6	Serrate RNA effector molecule homolog OS=Mus musculus GN=Srrt PE=1 SV=1 - [SRRT_MOUSE]	40.37	1.49	1	1	34.23	1.49	1	1	25.83	1.49	1	1	100.4
P28862	Stromelysin-1 OS=Mus musculus GN=Mmp3 PE=2 SV=2 - [MMP3_MOUSE]	29.67	1.47	1	1	33.24	1.47	1	1	44.91	1.47	1	1	53.8
P12960	Contactin-1 OS=Mus musculus GN=Cntn1 PE=1 SV=1 - [CNTN1_MOUSE]									26.63	1.37	1	1	113.3
P38647	Stress-70 protein, mitochondrial OS=Mus musculus GN=Hspa9 PE=1 SV=3 - [GRP75_MOUSE]	23.59	1.33	1	1		0.00							73.4
P13595	Neural cell adhesion molecule 1 OS=Mus musculus GN=Ncam1 PE=1 SV=3 - [NCAM1_MOUSE]					41.79	1.26	1	1	38.49	1.26	1	1	119.4
Q61495	Desmoglein-1-alpha OS=Mus musculus GN=Dsg1a PE=2 SV=2 - [DSG1A_MOUSE]		0.00							21.41	1.23	1	1	114.5
Q8BPM0	Disheveled-associated activator of morphogenesis 1 OS=Mus musculus GN=Daam1 PE=1 SV=4 - [DAAM1_MOUSE]									16.78	1.11	1	1	123.3
O70228	Probable phospholipid-transporting ATPase IIA OS=Mus musculus GN=Atp9a PE=2 SV=3 - [ATP9A_MOUSE]						0.00			0.00	1.05	1	1	118.5
P97350	Plakophilin-1 OS=Mus musculus GN=Pkp1 PE=2 SV=1 - [PKP1_MOUSE]	0.00	0.96	1	1		0.00							80.8
Q99P72	Reticulon-4 OS=Mus musculus GN=Rtn4 PE=1 SV=2 - [RTN4_MOUSE]					16.52	0.86	1	1	25.55	0.86	1	1	126.5
B9EKR1	Receptor-type tyrosine-protein phosphatase zeta OS=Mus musculus GN=Ptprz1 PE=1 SV=1 - [PRPTZ_MOUSE]									34.12	0.52	1	1	254.2
Q9QXS1	Plectin OS=Mus musculus GN=Plec PE=1 SV=3 - [PLEC_MOUSE]									17.60	0.17	1	1	533.9

Supplementary Table S3. The list of proteins identified in LC-MS/MS analysis of conditioned media from *Sst_1/Sst_4* dKO neuron.

Candidate proteins regulating NEP activity were screened by the absence or presence only in the media of the SST- and TT232-treated WT neurons in the range of 10 to 30 kDa molecular weight (Supplementary Table S2), but not in the media of *Sst_1/Sst_4* dKO neurons. Red: Control samples, Blue: SST-treated samples, Green: TT232-treated samples. ENSA, NSG1 and NUCKS1 were highlighted in yellow.

sgRNAs	sequence
sgRNA1-*ENSA*-Exon1	TAATACGACTCACTATACAGCCGGAAAAGGGAAGGGTTTTAGAGCTAGAA
sgRNA2-*ENSA*-Exon1	TAATACGACTCACTATAGCAGAGGCGGCCAATCAGGTTTTAGAGCTAGAA
sgRNA3-*ENSA*-Exon1	TAATACGACTCACTATACTGTGTAATGCAGGGCCCGTTTTAGAGCTAGAA
sgRNA4-*ENSA*-Exon1	TAATACGACTCACTATAGACCGGGGGTGGCCGCGCGTTTTAGAGCTAGAA
CRISPRscan tail primer	AAAAGCACCGACTCGGTGCCACTTTTTCAAGTTGATAACGGACTAGCCTTATTTTA ACTTGCTATTTCTAGCTCTAAAAC

Supplementary Table S4. The list of primers for *in vitro* transcription.

The table shows the list of primers for *in vitro* transcription. *In vitro* transcription was performed as previously described [32].

sgRNA1-*Ensa*-Exon1	DNA sequence	Chromosome	Position	Mismatches	exon or intron or intergenic	Gene	off-target
ON1	GGCAGCCGGAAAAGGGAAGGNGG						
OFF1	ATCAGCCAGAAAAGGGAAGGGGG	Chr17	22734904-22734926	3	non-coding RNA	Gm5224	-
OFF2	AGCACCCGGAAAAGGGAAGGCTG	Chr19	42449768-42449790	3	intergenic		-
OFF3	AGCAGCCGGGAATGGGAAGGAGG	Chr1	21328151-21328173	3	non-coding RNA	Gm28836	-
OFF4	AGCAGCCGGAGAAGGGAAGGAGA	Chr19	45150701-45150723	3	intron	Tlx1	-
OFF5	CGCAGCCGGGACAGGGAAGGCGG	Chr16	3884664-3884686	3	intergenic		-
OFF6	GACAGACGGAAAAGGGAAAGAGG	Chr14	75826276-75826298	3	intergenic		-
OFF7	GACAGCTGGAAGAGGGAAGGGGG	Chr3	88616844-88616866	3	intron	Arhgef2	-
OFF8	GCCAGCAGGAAAAGGGAAGGAGG	Chr1	5484362-5484384	2	intergenic		-
OFF9	GCCAGCAGGAACAGGGAAGGAGG	Chr14	23208188-23208210	3	intergenic		-
OFF10	GCCAGCAGGAACAGGGAAGGGGG	Chr9	6372785-6372807	3	intron	Pdgfd	-
OFF11	GCCAGCAGGAAAAGGGACGGAGG	ChrX	69241928-69241950	3	intergenic		-
OFF12	GCCAGCCAGGAAAGGGAAGGAGG	Chr14	70176714-70176736	3	exon	Pdlim2	-
OFF13	GTCAGCAGGAAAAAGGAAGGAGG	Chr2	121048751-121048773	3	intron	Tgm5	-
OFF14	GTCAGCAGGAAAAAGGAAGGTGG	Chr7	92274412-92274434	3	intron	Dlg2	-
OFF15	GGAAGACGGAAGAGGGAAGGGGG	Chr4	40899677-40899699	3	non-coding RNA	Gm34289	-
OFF16	GGAAGGGGGAAAAGGGAAGGGGG	Chr6	58528517-58528539	3	non-coding RNA	Gm35077	-
OFF17	GGGGGCAGGAAAAGGGAAGGAGG	Chr8	94971295-94971317	3	non-coding RNA	Gm30968	-
OFF18	GGGGGCGGGAAAAGGGAAGGGGG	Chr10	5698475-5698497	3	intergenic		-
OFF19	GGGAACCGGAAAAGGGAAAGTGG	Chr8	43330475-43330497	3	intergenic		-
OFF20	GGGAGCCGGACAAGGGGAGGGGG	Chr7	24610358-24610380	3	non-coding	Phldb3	-
OFF21	GGCGGCAGGGAAAGGGAAGGCGG	Chr15	99973187-99973209	3	inton	Larp4	-
OFF22	GGCTGCCGGAGAAGGCAAGGCGG	Chr2	11503395-11503417	3	intron	Pfkfb3	-
OFF23	GGCAACAGGAAAAAGGAAGGTGG	Chr4	43230789-43230811	3	intron	Unc13b	-
OFF24	GGCAACTGGGAAAGGGAAGGAGG	Chr17	59981721-59981743	3	intergenic		-
OFF25	GGCAGAGGGAAAAGGGAGGGAGG	Chr7	108688197-108688219	3	intergenic		-
OFF26	GGCAGAGGGAAAAGGGAAGAAGG	ChrX	71980668-71980690	3	intron	Fate1	-
OFF27	GGCAGACGGAAAAGGGAGGGTGG	Chr2	148875517-148875539	2	intergenic		○
OFF28	GGCAGGAGAAAAAGGGAAGGGGG	Chr1	38903926-38903948	3	intergenic		-
OFF29	GGCAGGGGGAAAGGGGAAGGGGG	Chr3:	133630455-133630477	3	intergenic		-
OFF30	GGCAGGCAGAAAAGGGAGGGAGG	Chr1	143917569-143917591	3	intergenic		-
OFF31	GGCAGGCGGAAGAGGGAAGAGGG	Chr16	50360874-50360896	3	intron	Bbx	-
OFF32	GGCAGGCGGAAAAGGGAGGGCTG	Chr9	12553043-12553065	3	intergenic		-
OFF33	GGCAGGCGGAAAAGGGAGGGCTG	Chr12	43779383-43779405	3	non-coding RNA	Gm33384	-
OFF34	GGCAGCAGGAACAGGGAAGATGG	Chr5	36058754-36058776	3	intron	Sorcs2	-
OFF35	GGCAGCAGGAAGAGGGAGGGAGG	Chr2	17311178-17311200	3	intergenic		-
OFF36	GGCAGCAGGAAAGGGGAAGATGG	Chr1	132335196-132335218	3	intergenic		-
OFF37	GGCAGCAGGAAAAGTGAGGGTGG	Chr6	108314289-108314311	3	intron	Itpr1	-
OFF38	GGCAGCTGGAAGAGGGATGGAGG	Chr1	185474707-185474729	3	intergenic		-
OFF39	GGCAGCTGGAAAACGGAAGGGGA	Chr1	127556223-127556245	3	intron	Tmem163	-
OFF40	GGCAGCCAGAAAAGGGTAGGTGG	Chr17	71302546-71302568	2	intron	Emilin2	-
OFF41	GGCAGCCGGGAAGGGGAAAGGGG	Chr8	11053937-11053959	3	non-coding RNA	9530052E02Rik	-
OFF42	GGCAGCCGGAACAGGACAGGGGG	Chr2	167656445-167656467	3	intron	Tmem189	-
OFF43	GGCCAGCAGGAAAAGGGAAGGAGG	Chr1:	5484362-5484385	1 (insertion)	intergenic		-
OFF44	GGCGGCCGGAGAAAGGGAAGGGGG	Chr10:	18318596-18318619	1 (insertion)	intron	Nhsl1	-

sgRNA2-*Ensa*-Exon1	DNA sequence	Chromosome	Position	Mismatches	exon or intron or intergenic	Gene	off-target
ON1	GGGCAGAGGCGGCCAATCAGNGG						
OFF1	GCGCGGAGGCGGCGAATCAGAGG	Chr4	152178127-152178149	3	exon	Acot7	-
OFF2	GTGCAGAGGCAGCCAATCAGAAG	Chr7	48886629-48886651	3	intergenic		-
OFF3	GGACAGAGGCAGACAATCAGAGG	Chr12	78944736-78944758	3	non-coding RNA	Gm46359	-
OFF4	GGTCAGAGGAGGCCATTCAGTGG	Chr14	67739776-67739798	3	intron	Kctd9	-
OFF5	GGGGAGAGGAGGCCAATCAGAGT	Chr3	152064575-152064597	3	intergenic		-
OFF6	GGGCACAGGCAGCCAATCCGAGG	Chr14	59116156-59116178	3	intergenic		-
OFF7	GGGCAGATGCGGCCAATCAAAGA	Chr3	95069511-95069533	3	intron	Pip5k1a	-
OFF8	GGGCAGAGCTGGCTAATCAGGGG	Chr7	45936086-45936108	3	intron	Ccdc114	-
OFF9	GGGCAGAGGCAGCCAATTAGTGT	Chr11	60145612-60145634	3	intron	Rai1	-
OFF10	GGGCAGAGGCGGCCAATCTGTGT	Chr9	21617732-21617754	2	intron	Smarca4	-
OFF11	AGGCAGAGCGGCCAATCAGGGG	Chr11	95842203-95842224	1	intron	Abi3	-

Supplementary Table S5. Predicted Off-target regions in *Ensa* KO mice generated by CRISPR/Cas9.

The table shows the list of off-target regions predicted by COSMID in *Ensa* KO mice [33].

	Forward	Reverse
**PCR Primers (sgRNA1)**		
ON1	CCATTTTGACTGAGCAACCA	TTCATTCGCCTCATCTTTCC
OFF1	CTTGTGTATCTTGCTTCTCCTCCC	GAGGCTGTCACTATGGCTCATTAC
OFF2	TCTTGGTGACAGTGACCTCCAG	GCCACTGTCTTAGCACTTCTTAGC
OFF3	CCAGGTCTTCTTGCTTCACTATCC	TTTATTTCCTCCTCCTCCTCCTCC
OFF4	CACGCACAGCCAATGGAGAG	ATCGCTGCGCTGGACTACC
OFF5	GACGGTGTCTCAAGGCTCAG	AGCTGTCTTGAGCTTCCGCC
OFF6	CTGGGATGTAGGCACAGGAG	CTGATGAGAAGAGGGCATAACTGG
OFF7	ACCCTCTGAATAAGGGAAAGGCAC	CCCAAAGCTCCTGTTAGAGACTCA
OFF8	TGTTAGGGGTTCTGTGGCGTG	CCCTGCCATCTTTTTTCTGAACCC
OFF9	GGTCCTGTGGCATGGAGAGT	GGCCACCCCTGCCATATTCT
OFF10	GAGGTGTGGAGAGTTCTCTGG	CACTGCTCAACAGGAAGACGTAC
OFF11	GGGGGTTATGGGCGACTTTT	GCTCGCTTGATATCACTGGGA
OFF12	ATCACATGGGCGGAGGCAGGT	TCAGGGGAGAACCCAAGCCA
OFF13	CTGGTTCCATTCTGGCAGGAC	CCTACAGACACCAAGCTTTGCTGA
OFF14	CTGTGATCACAGCAGGCAGC	GGGTATAGGGGACTTTCAGGTTAG
OFF15	GTGAGCCAGGTTTTGTGGGCA	GTCCACGGGGAAGTAGATGTC
OFF16	AGGAGGAGAGGAGGAGAGGA	CTTTACCCCTCCCCCCTTTC
OFF17	CAGTCTACAGACAGCAAGTCCTGA	ACCGAATTCCTCCCCTCCTC
OFF18	AAGGGAAGGGAAAGGGGAGG	CTTTTGTCCCTTTTCTCCTCTCCC
OFF19	TCACGTCGACTTTCTGTTCTCGAG	ACTAGTAAGAGTGTAAGTGGGGCG
OFF20	CCTGTTTCTGCCCAGAGAGGA	CTTAGCCACCTGCATACAGCAGC
OFF21	TGATCGCCCTTTCTCCCGCAG	TGACTTTCCAGGCCGTCCAC
OFF22	GTGGAAAAGTCTGCGCAGAGG	CTGGGGTTGGGGACAGGTAT
OFF23	GCTTAGCTGCTGCCCATAACTG	GGCTGGCCTTTTCAAACAATGTGC
OFF24	CCCATTGTTCAAGTCACCTGTGC	GCACAGTAACCCTGGTTCAGTAG
OFF25	GTCATAAGACTTAGCTGCAGTCCC	TAGATGGTCACGCTACCTCTCAC
OFF26	CAGCTGGACTTTGGTAATCAGTCG	TAGCCTATCTGCTGAGTTCGGCTT
OFF27	GGAATCTGGCAGCTCTTTAAGAGG	GGCTAGGACAAAGGGATAAAGCTC
OFF28	GCTCCTCCAAGAGATCTGTGAC	GTCAGCCCAAGGTCAATGAAGC
OFF29	TCCTTTGTGTACAGAAAGCCCA	AAAGGGTAAAGGGCAGGGGG
OFF30	GGGGAAAAACGTGCCAGGAG	CAACCTTTCCTAGCTTCTCCCAG
OFF31	CGAGCTGATTCCTCAGGAGAC	GTGTGTGATCCACTCATGGATTTGCTTTTC
OFF32	CCTGATGGGAACAAAAAAAGGGGG	TCAGTGTTGGGAACCTCTTCCCT
OFF33	ACCACTCTTGTCCATCACTGTGGG	TGGGAACCTCTCTCCCTAAGC
OFF34	TGCAAAGTCACCAGGGATGTAGG	TAACTGCTGCTCAGCTTCACAGG
OFF35	CTGTGAAGAAACATGACCACCGC	GGCAGTATTAGGAGTTGTGGCCTT
OFF36	ACCACGGGAACTCCACCAAC	GCAAACATCTACACCTACCAGGG
OFF37	GATGTTGGCAGTCACCTGAGAG	CCTGTTCCTGCCTTACTCAGTATC
OFF38	ACTTCCTTCTTTGCATTCCCTGCC	AGGTTGTCCATGCTCCACGG
OFF39	CTTGGTGGCTCCCTTCTGTATTTC	GCTACATCACAGAAGGCACACAAC
OFF40	CGACACATGTTGTCATCCACAAGC	CCCCTCCACATACACACAAAACC
OFF41	CTGGCACTACATAGGACAGGTCA	GGGAACTTCTACATTCTGCACCCA
OFF42	TGGTAGAACGTTCGTTCTTGGGG	CAGTTACTGTTACCAGGCACCAAG
OFF43	TGTTAGGGGTTCTGTGGCGTG	CCCTGCCATCTTTTTTCTGAACCC
OFF44	GAGAAAGTTCCCGGAGCAG	GAGCTGCCGAGTTCTTGGTT
**PCR Primers (sgRNA2)**		
	Forward	Reverse
ON1	CCATTTTGACTGAGCAACCA	TTCATTCGCCTCATCTTTCC
OFF1	CGGCGGAATCTGAGAACAGG	TCTGGAAGCGGAGTCCCACA
OFF2	TTCAGACATGTAAAGAAAGTCCCTGACG	GGGCCTTACAAGGAGAAGATGAG
OFF3	AAGACTGGGACGCCACCTTG	GTCAGAACCAGGAAGAACATCCTC
OFF4	ACTTGCCACCACACTCTTTGCC	GGAGCAGAGCAGCAAGATCAAC
OFF5	CACAAGACAGATATGAGTGGGGAC	GTAAGCAGGCATCTCCAGGC
OFF6	ATATCTTGCAGGCAGGGCAC	CAGAGACCCACAACGGAGTC
OFF7	CCAGGTAAATAAAGTGAGAAAGGACCAI	ACCCTTGGCTAAACTGCACTGTC
OFF8	AGCCAGGGATATACAGTAAGGCC	CCTGGGAGAGATGCATCAAGGA
OFF9	CCCGTTAACCCAAGGAACGC	CTTGCCAGGAAAAGCTACCGTC
OFF10	TATGGGGACGTTTCTCCTTG	AGGGCTTTGCTAATGCAAGA
OFF11	CGGCCCGTGGGGATATCAAA	CCTTCTCCTGAGCAGGGTAC

Supplementary Table S6. The list of primers to search for off-target in *Ensa* KO mice.

The table shows the list of primers for off-target analysis in *Ensa* KO mice.

Primers (For Genotyping and RT-PCR)		
Target gene	Forward	Reverse
Ensa	5'-CCATTTTGACTGAGCAACCA-3'	5'-TTCATTCGCCTCATCTTTCC-3'
Abcc8	5'-GTGTGGTGGTCCCTGCTTAG-3'	5'-CCTCACGCACTTCAGTCACT-3'
Kcnj8	5'-AATGGCCGATCCCATATTGG-3'	5'-CTGAACTGGTGATGAGTGCA-3'
Kcnj11	5'-TAGGCCAAGCCAGTGTAGTG-3'	5'-GCCCTGCTCTCGAATGTTCT-3'

Supplementary Table S7. Primers for Genotyping and RT-PCR.

The table shows the list of primers for genotyping and RT-PCR.

Sequence	# PSMs	# Proteins	Protein Grou	rotein Group Accessio	Modifications	ΔCn	IonScore	Exp Value	Charge	MH+ [Da]	ΔM [ppm]	RT [min]	# Missed Cleavages
LVTGDHIPTPQDLPQRKSS	3	1	1	sp		0.0000	35	0.000289048	3	2089.10556	0.88	24.71	0
GDHIPTPQDLPQRKSS	3	1	1	sp		0.0000	33	0.00055077	3	1775.90476	0.67	20.62	0
MKNKQLPSAGPDKNLVTGDHIPTPQDLPQRKSS	1	1	1	sp		0.0000	28	0.001674827	5	3597.88499	1.18	25.29	0
KNKQLPSAGPDKNLVTGDHIPTPQDLPQRKSS	2	1	1	sp		0.0000	24	0.004295068	5	3466.84431	1.17	24.71	0
LGQKPGGSDF	1	1	1	sp		0.0000	18	0.016179683	2	1005.50029	0.25	17.83	0

Supplementary Table S8. Amino acid sequences of ENSA cleaved by NEP.

The table shows the list of amino acid sequences of ENSA cleaved by NEP.

For histological analysis								
Sample	Catalog/Sample ID	Lot	Gender	age	PMI	Region	Neuropathological diagnosis	Import source
non-AD	TS2013-104	TS2013-104	M	79	15	Frontal Cortex	Healthy control	Tissue solutions
non-AD	TS2013-106	TS2013-106	M	79	17	Frontal Cortex	Healthy control	Tissue solutions
non-AD	TS2013-108	TS2013-108	F	91	19.5	Frontal Cortex	Healthy control	Tissue solutions
non-AD	T2234051	A711156	M	73	5	Frontal Cortex	Healthy control	Biochain
non-AD	T2234051	C301017	M	54	5	Frontal Cortex	Healthy control	Biochain
AD	-	-	F	84	17	Motor Cortex	AD	from Pennsylvania
AD	-	-	F	77	5	Parietal Cortex	AD	from Pennsylvania
AD	-	-	F	74	6	Frontal Cortex	AD	from Pennsylvania
AD	TS2013-100	TS2013-100	M	79	5.4	Frontal Cortex	AD	Tissue solutions
AD	TS2013-102	TS2013-102	F	94	12.8	Frontal Cortex	AD	Tissue solutions
AD	TT2236051Alz	B901022	M	73	4-8	Frontal Cortex	AD	Biochain
For biochemical analysis								
Sample	Catalog/Sample ID	Lot	Gender	age	PMI	Region	Neuropathological diagnosis	Import source
non-AD	P1234042	B909014	M	66	8	Cerebral Cortex	Healthy control	Biochain
non-AD	P1234042	B211002	M	75	4	Cerebral Cortex	Healthy control	Biochain
non-AD	P1234042	C110125	M	78	5	Cerebral Cortex	Healthy control	Biochain
AD	-	-	M	80	n/a	Frontal/Temporal Cortex	AD	from Pennsylvania
AD	-	-	M	76	n/a	Frontal/Temporal Cortex	AD	from Pennsylvania
AD	-	-	F	62	n/a	Parietal/Temporal Cortex	AD	from Pennsylvania
AD	-	-	M	70	n/a	Frontal/Temporal Cortex	AD	from Pennsylvania
AD	-	-	F	92	n/a	Parietal/Temporal Cortex	AD	from Pennsylvania

Supplementary Table S9. The list of human samples.

The table shows information of human samples from left to right: Sample, Catalog/Sample ID, Lot, Gender, Age, Postmortem time, Region, Neuropathological diagnosis, Import source.

Analysis	Cohort	Number of samples	Gene	Region	Log Fold Change	Adjusted P-Value	significant
AD vs Control	GSE15222	Control n = 135<br>AD n = 106	*ABCC8*	Temporal Cortex	-0.416	**1.49E-07**	***
			ABCC9		0.0094	0.944	n.s
			KCNJ8		0.189	**4.20E-03**	*
			KCNJ11		-0.138	0.171	n.s
	GSE95587	Control n = 33<br>AD n = 84	*ABCC8*	Fusiform gyrus	-0.377	**6.08E-03**	*
			ABCC9		0.226	0.154	n.s
			KCNJ8		0.351	**9.06E-03**	*
			KCNJ11		-0.0262	0.848	n.s
	GSE125583	Control n = 70<br>AD n = 219	*ABCC8*	Fusiform gyrus	-0.363	**3.59E-05**	***
			ABCC9		0.264	0.0452	*
			KCNJ8		0.362	**1.09E-05**	***
			KCNJ11		-0.074	0.379	n.s
	Van Rooji *et al.*, 2019	Control n = 10<br>AD n = 18	*ABCC8*	Hippocampus	-1.29	**2.08E-11**	***
			ABCC9		0.055	0.795	n.s
			KCNJ8		0.653	**5.67E-07**	***
			KCNJ11		-0.505	**4.89E-04**	***

Supplementary Table S10. Comparison of gene expression for each K_{ATP} channel component between AD patients and controls.

Table showing the microarray and RNA sequencing (RNA-seq) statistical data for gene expression relating to each K_{ATP} channel component between AD patients and controls as derived from public cohort studies.

Gene	Cohort	Braak stage	Sample number	Mean of Log2(rpkm+1)	P-value	significant
ABCC8	GSE95587	Ⅲ	6	3.202	Ⅲ vs. Ⅵ p=0.0123	*
		Ⅳ	38	2.671		
		Ⅴ	21	2.655		
		Ⅵ	19	2.482		
	GSE125583	Ⅰ	1	3.46	Ⅲ vs. Ⅵ p=0.0015	**
		Ⅱ	5	2.95	Ⅳ vs. Ⅵ p=0.0006	***
		Ⅲ	14	3.059	Ⅴ vs. Ⅵ p=0.0006	***
		Ⅳ	44	2.897		
		Ⅴ	48	2.887		
		Ⅵ	44	2.442		
KCNJ11	GSE95587	Ⅲ	6	2.237		
		Ⅳ	38	1.986		
		Ⅴ	21	1.917		
		Ⅵ	19	1.871		
	GSE125583	Ⅰ	1	3.02	Ⅲ vs. Ⅵ p=0.0199	*
		Ⅱ	5	2.428		
		Ⅲ	14	2.562		
		Ⅳ	44	2.342		
		Ⅴ	48	2.333		
		Ⅵ	44	2.155		

Supplementary Table S11. Gene expression of *ABCC8* and *LCNJ11* with differentiating Braak stages.

Table showing RNA-seq statistical data for *ABCC8* and *KCNJ11* gene expression with differentiating Braak stages as derived from public cohort studies.
