# Supplementary material for: Somatostatin-evoked Aβ catabolism in the brain: Mechanistic involvement of α-endosulfine-KATP channel pathway
Source: Mol Psychiatry. 2021 Nov 4;27(3):1816–28. doi: 10.1038/s41380-021-01368-8 (PMC9095489; doi:10.1038/s41380-021-01368-8)
Supplement: Supplementary file 2 — Supplementary Materials [file 41380_2021_1368_MOESM2_ESM.docx]

Supplementary Materials


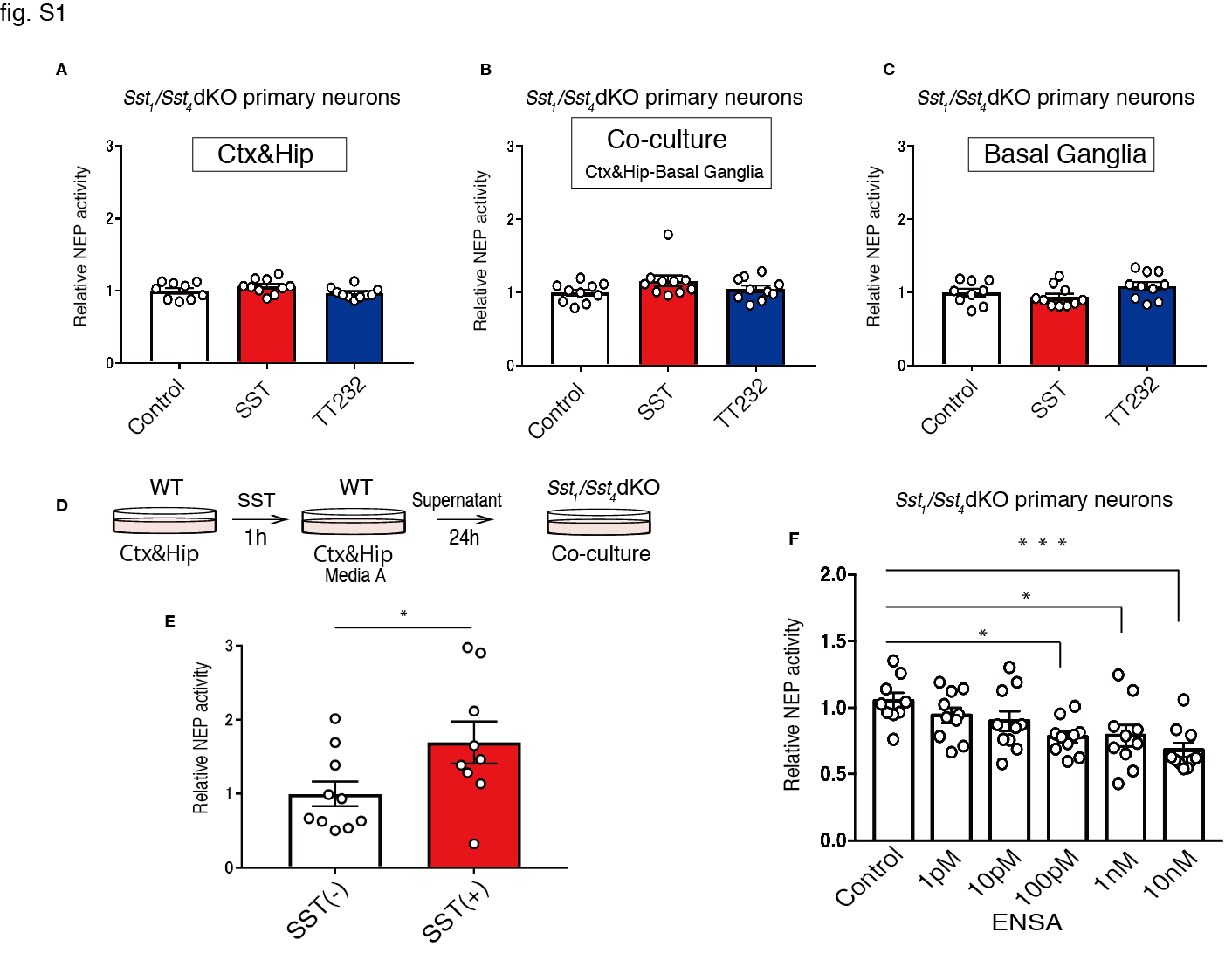


**Supplementary** **Figure S1. NEP activity in primary neurons from *Sst_1_/Sst_4_* dKO mice.**

A-C. NEP activity after treatment of primary neurons derived from *Sst_1_*/*Sst_4_* dKO mice with 1 μM SST or TT232 for 24 hours. (A) Cortical/hippocampal (Ctx&Hip) neurons (n = 9-10 wells per treatment), (B) co-cultured neurons (n = 10 wells per treatment), and (C) basal ganglia neurons (n = 9-10 wells per treatment) were used. D and E. NEP activity of co-cultured neurons from *Sst_1_*/*Sst_4_* dKO mice after replacement of the culture medium with conditioned media derived from SST-treated Ctx&Hip neurons from WT mice (n = 9-10 for each group).

F. NEP activity of co-cultured neurons derived from *Sst_1_*/*Sst_4_* dKO mice after treatment with different doses of recombinant ENSA protein (n = 9-10 for each group). In (E)**,** the data represent the mean ±SEM. **P*<0.05 (Student’s *t*-test). In (F**),** the data represent the mean ±SEM. **P*<0.05, ***P*<0.01, ****P*<0.001 (one-way ANOVA with Dunnett’s post-hoc test).


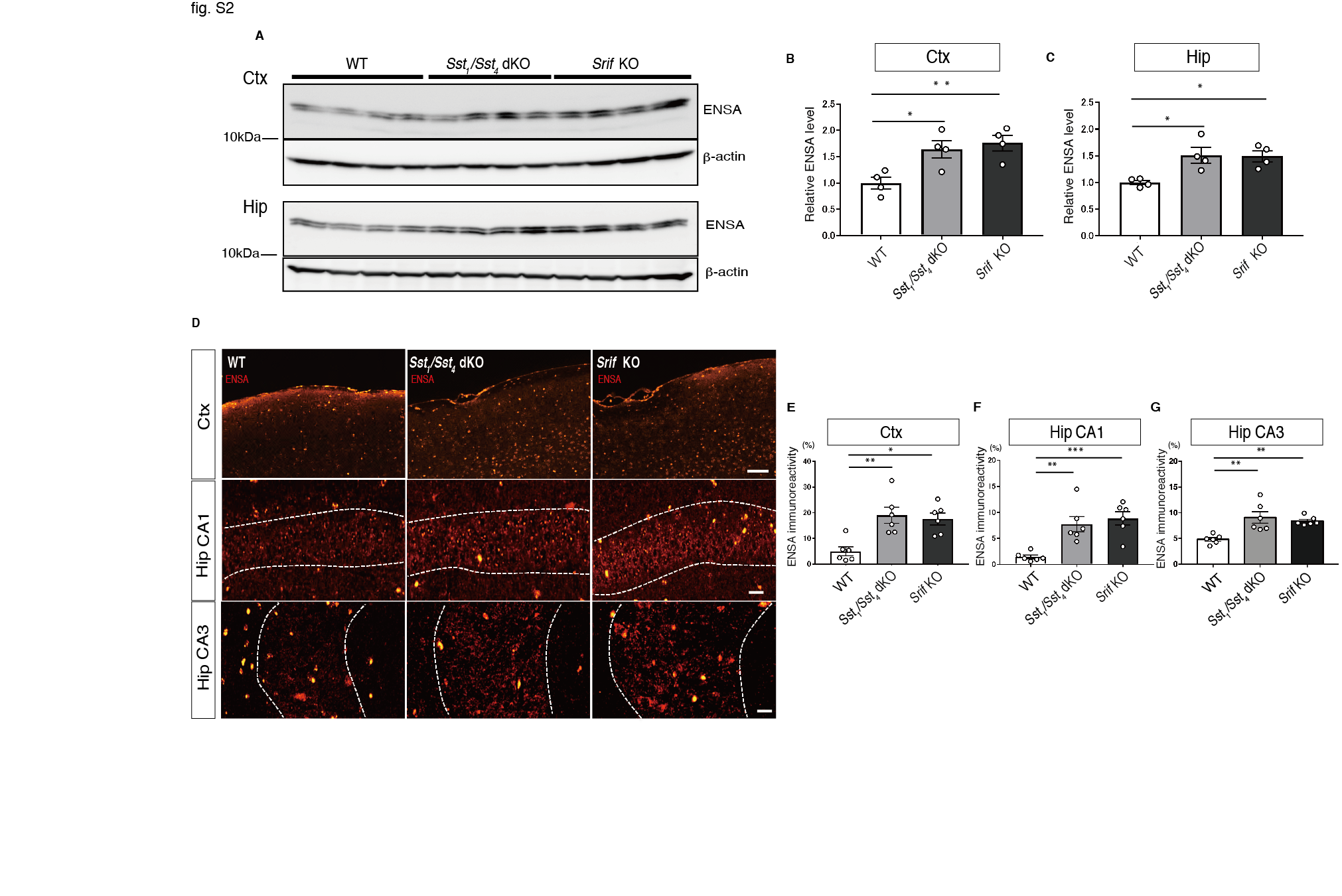


**Supplementary** **Figure S2 ENSA levels in *Sst_1_/Sst_4_*- and *Srif-*deficient mice.**

A-C. Immunoblotting of ENSA in the (B) cortices and (C) hippocampi from 3-month-old WT, *Sst_1_/Sst_4_* dKO and *Srif* KO mice (n = 4 for each group). Values indicated in the graph show ENSA band intensities normalized to that of β-actin. D-G. Immunostaining of ENSA in the (E) cortices and (F) hippocampal CA1 and (G) CA3 regions from 3-month-old WT, *Sst_1_/Sst_4_* dKO, and *Srif* KO mice (n = 6 for each group). Scale bar is 200 µm in cortical image and 20 µm in hippocampal image. Data represent the mean ±SEM. **P*<0.05, ***P*<0.01, ****P*<0.001 (one-way ANOVA with Dunnett’s post-hoc test).

**
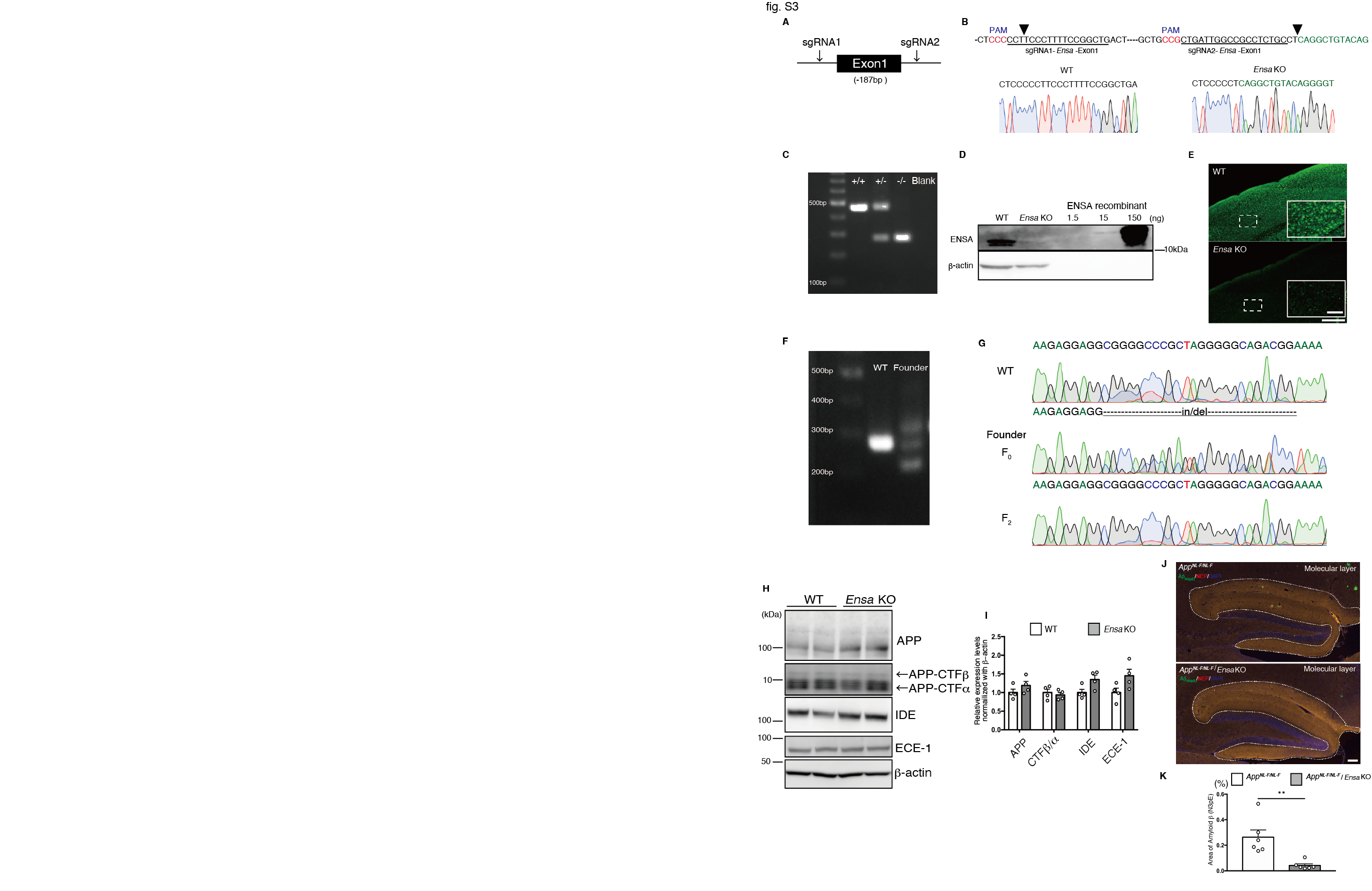
**

**Supplementary** **Figure S3. Generation of ENSA-deficient mouse using CRISPR/Cas9.**

A. Schematic image for CRISPR/Cas9-mediated ENSA deficiency. B. Sanger sequence chromatograms near exon 1 of *Ensa* gene in WT and *Ensa* KO mice. Arrowheads show Cas9 cleavage sites. C. PCR-based genotyping results of WT, heterozygous and homozygous *Ensa* KO mice. Genotyping was performed using mouse tail genome. D. Immunoblotting of ENSA in WT and *Ensa* KO mice. E. Immunostaining of ENSA in WT and *Ensa* KO mice. ENSA immunoreactivity is absent in *Ensa* KO mice. Scale bar = 500 µm. Inset Scale bar = 50µm. F. PCR-based genotyping results of off-target sites in WT and founder *Ensa* KO mice. Genotyping was performed using mouse tail genome. G. Sanger sequence chromatograms of off-target sites in WT, founder mouse and F2 *Ensa* KO mouse. H. Immunoblotting of APP, CTFs, IDE and ECE-1 in 3-month-old WT and *Ensa* KO mice. I. Values indicated in graphs show band intensities for APP, CTFs, IDE and ECE-1 normalized to that of β-actin (n = 4 for each group). J and K. Immunostaining of Aβ_N3(pE)_ (Green), NEP (Red) and DAPI (blue) from 18-month-old *App^NL-F^* and *App^NL-F^*/*Ensa* KO mice. Statistical analysis of amyloid β_N3(pE)_-positive area in 18-month-old *App^NL-F^* and *App^NL-F^*/*Ensa* KO mice (n = 6 for each group). Scale bar is 100 µm. Results are expressed as the mean ±SEM. ***P*<0.01 (Student’s *t*-test).

**
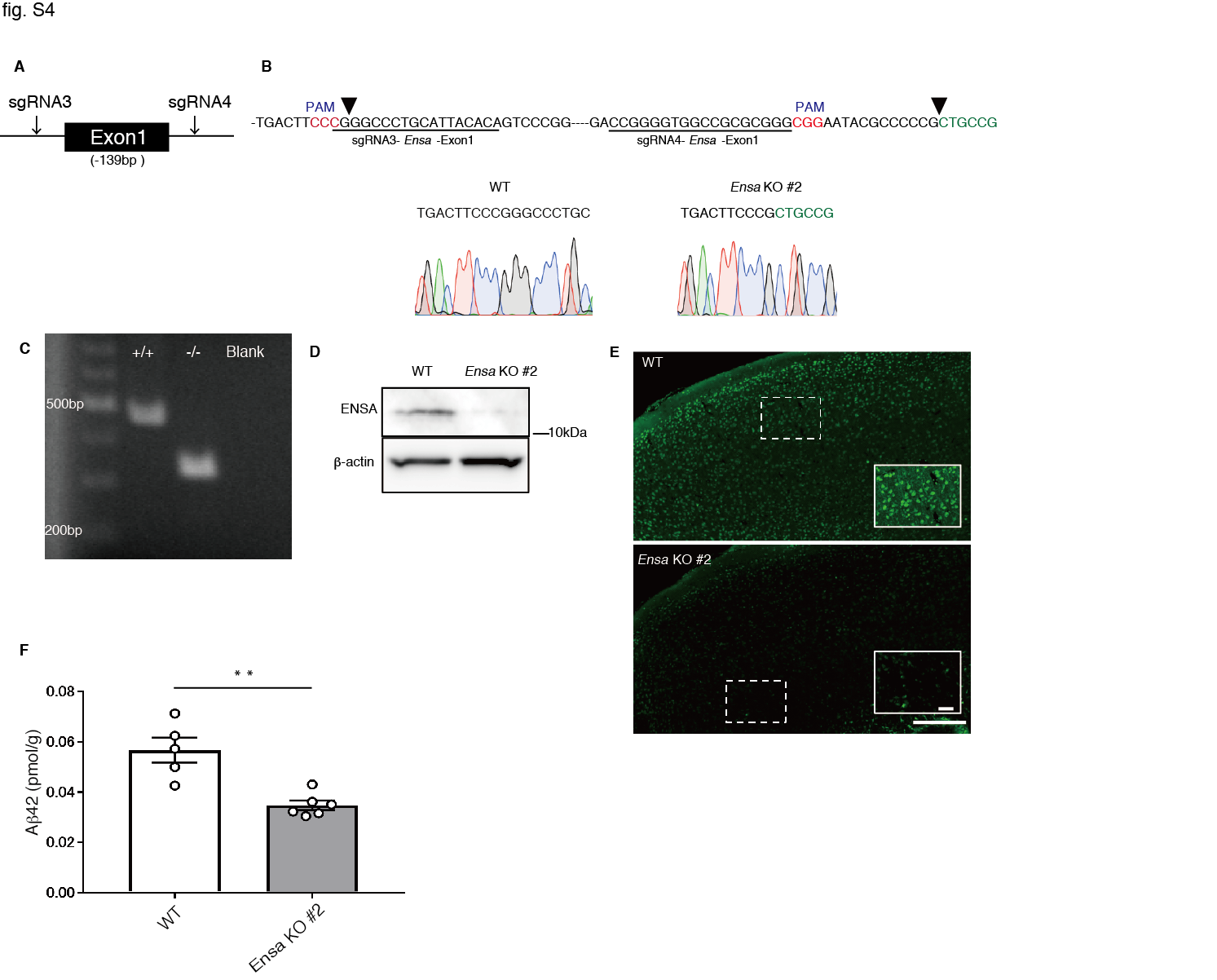
**

**Supplementary** **Figure S4. Generation of 2nd line ENSA-deficient mouse using CRISPR/Cas9.**

A. Schematic image for CRISPR/Cas9-mediated ENSA deficiency. B. Sanger sequence chromatograms near exon 1 of *Ensa* gene in WT and *Ensa* KO #2 mice. Arrowheads show cleavage sites by Cas9. C. PCR-based genotyping results of WT and *Ensa* KO #2 mice. Genotyping was performed using mouse tail genome. D. Immunoblotting of ENSA in WT and *Ensa* KO #2 mice. E. Immunostaining of ENSA in WT and *Ensa* KO #2 mice. ENSA immunoreactivity was absent in *Ensa* KO #2 mice. Scale bar = 500 µm. Inset scale bar = 50 µm. F. Aβ_42_ ELISA of hippocampi from 3-month-old WT and *Ensa* KO #2 mice (WT: n = 5, *Ensa* KO #2: n = 6). Results are expressed as the mean ±SEM. ***P*<0.01 (Student’s *t*-test).


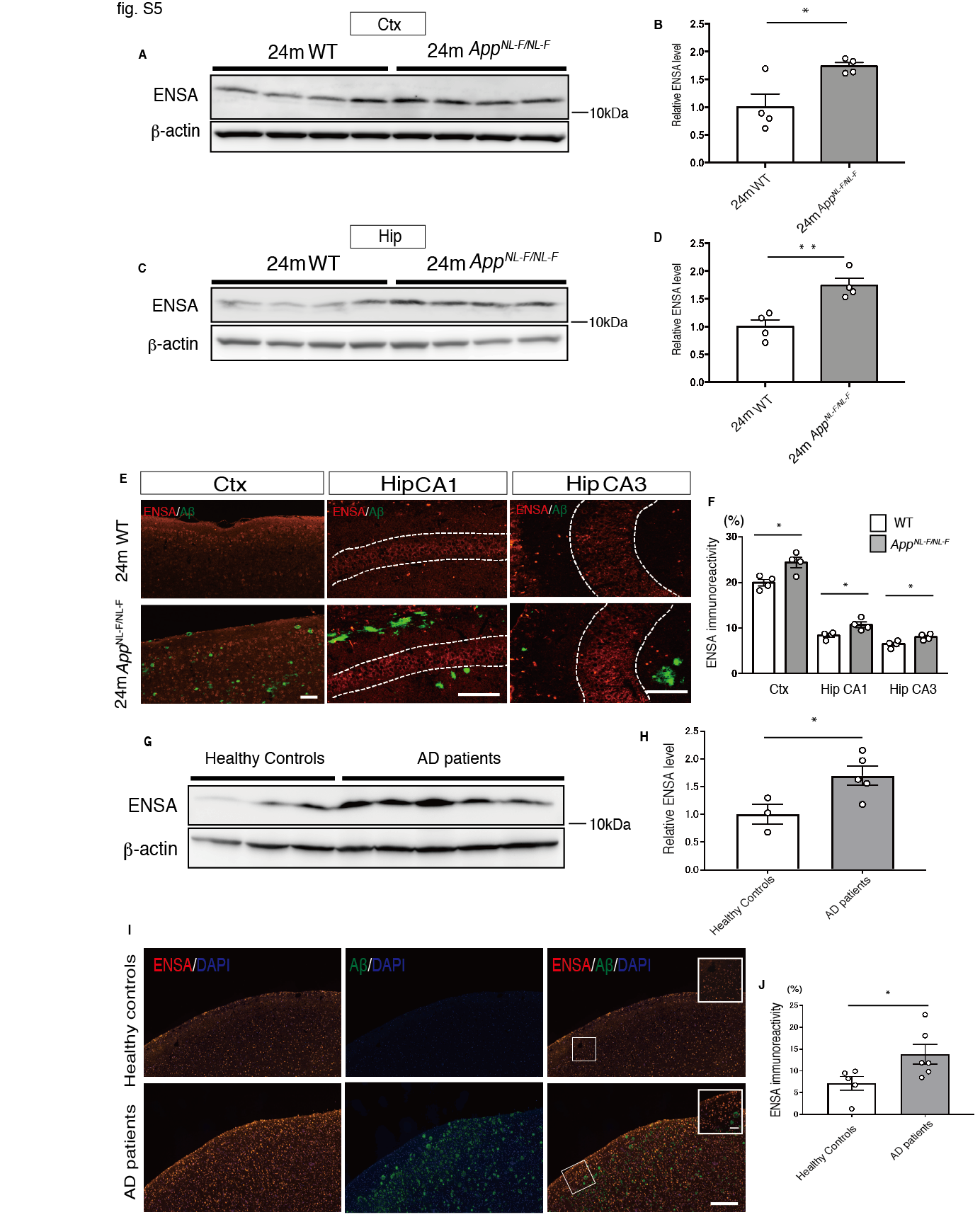


**Supplementary** **Figure S5. Increased levels of ENSA in AD model mouse and postmortem brain tissue from patients with AD.** A-D. Immunoblotting of ENSA in (A and B) cortices and (C and D) hippocampi of 24-month-old WT and *App^NL-F^* mice. Values indicated in the graph show ENSA band intensities normalized to that of β-actin (n = 4 for each group). E and F. Immunostaining of ENSA (Red) and Aβ (Green) in cortex, and hippocampal CA1 and CA3 regions of 24-month-old WT and *App^NL-F^* mice (n = 4 for each group). Scale bar is 100 µm. G and H. Immunoblotting of ENSA in cortices of healthy controls and AD patients. Values indicated in the graph show ENSA band intensities normalized to that of β-actin (healthy controls: n = 3, AD patients: n = 5). I and J. Immunostaining of ENSA in cortices of healthy controls and AD patients (healthy controls: n = 5, AD patients: n = 6). Scale bar is 500 µm in low-magnification image and 100 µm in high-magnification image. Data represent the mean ±SEM. **P*<0.05, ***P*<0.01, (Student’s *t*-test). Information concerning human samples is given in Supplementary Table S9.


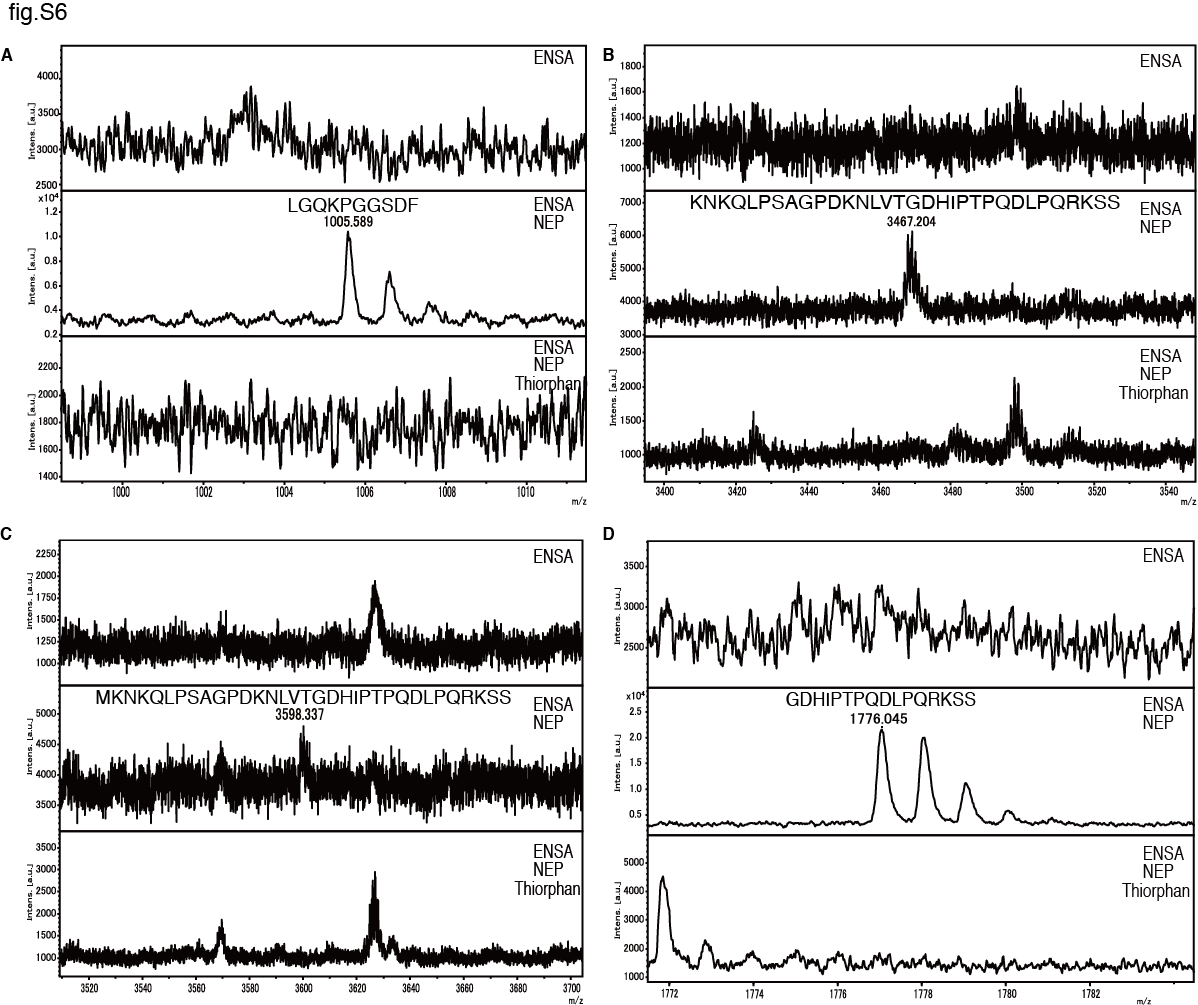


**Supplementary** **Figure S6. Specific peaks of ENSA cleaved by NEP.**

A-D. MALDI-TOF analyses showing specific peaks of cleaved ENSA after incubation in the presence or absence of NEP and thiorphan for 24 hours at 37˚C. LC-MS/MS analysis was used to determine specific amino acid sequences (Supplementary Table S8).

**
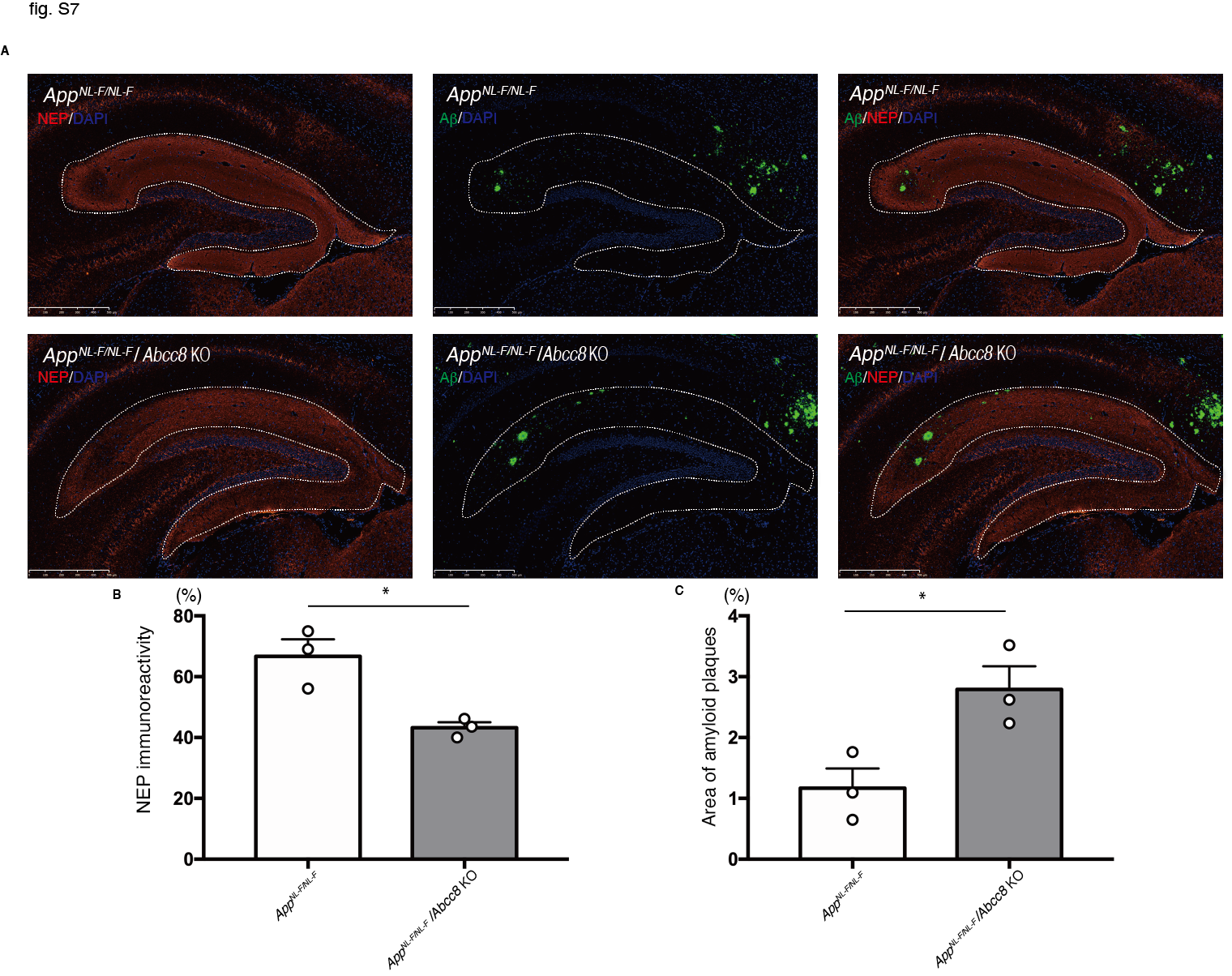
**

**Supplementary** **Figure S7. Increase of amyloid deposition in *App^NL-F^*/*Abcc8* KO mice.**

A. Immunostaining of NEP (Red), Aβ (Green) and DAPI (blue) from 12-month-old *App^NL-F^* and *App^NL-F^*/*Abcc8* KO mice. B and C. Statistical analysis of NEP and amyloid β positive signals in 12-month-old *App^NL-F^* and *App^NL-F^*/*Abcc8* KO mice (n = 3 for each group). Scale bar is 500 µm. Results are expressed as the mean ±SEM. **P*<0.05 (Student’s *t*-test).


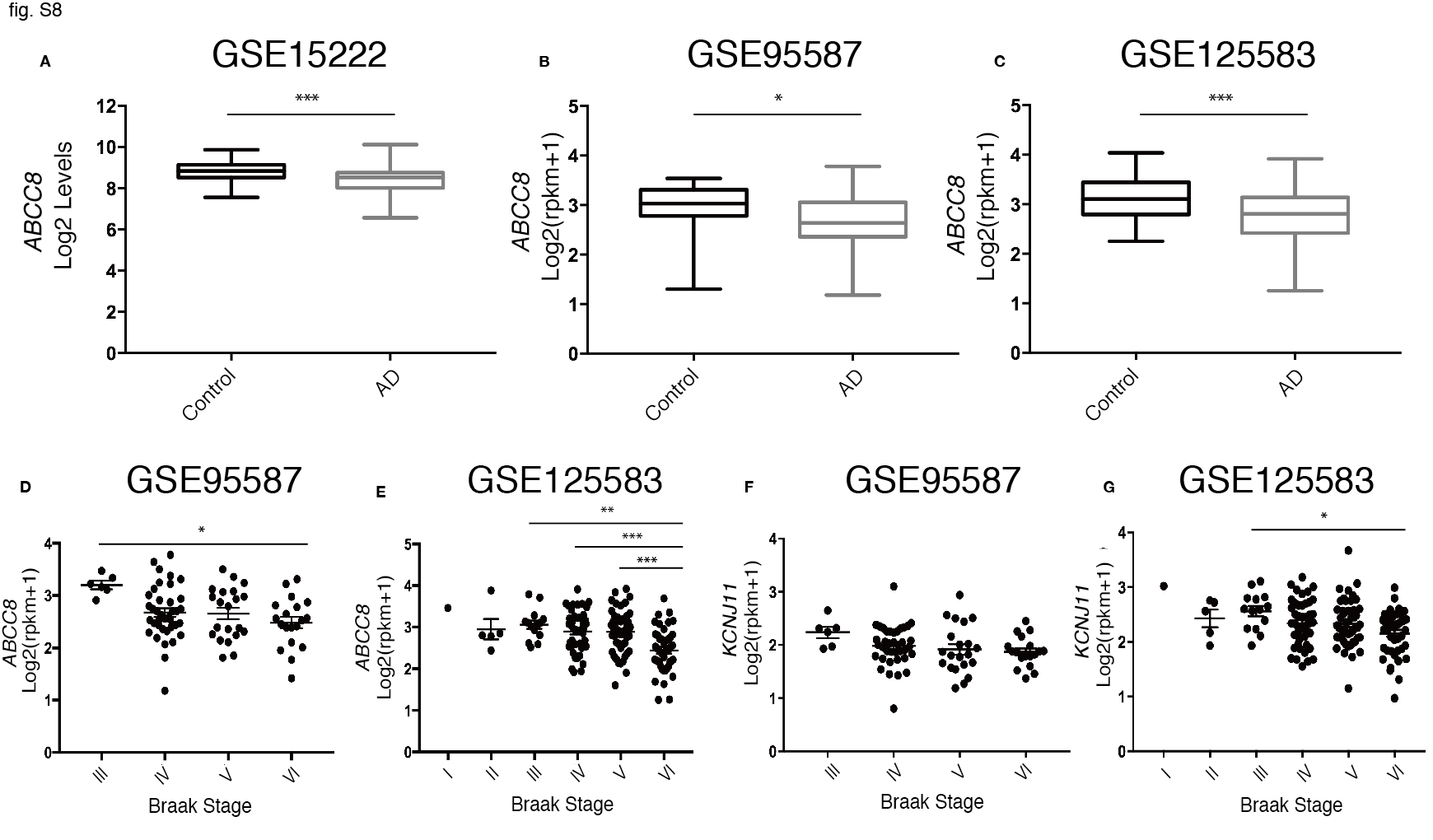


**Supplementary** **Figure S8. Gene expression of K_ATP_ channel components in AD patients.**

A-C. Gene expression of *ABCC8* mRNA in healthy controls and AD patients in the GSE15222, GSE95587 and GSE125583 cohorts. D-G. Gene expression of *ABCC8* and *KCNJ11* mRNA levels with differentiating Braak stage in the GSE95587 and GSE125583 cohorts. Statistical data are summarized in Supplementary Table S10 and S11.


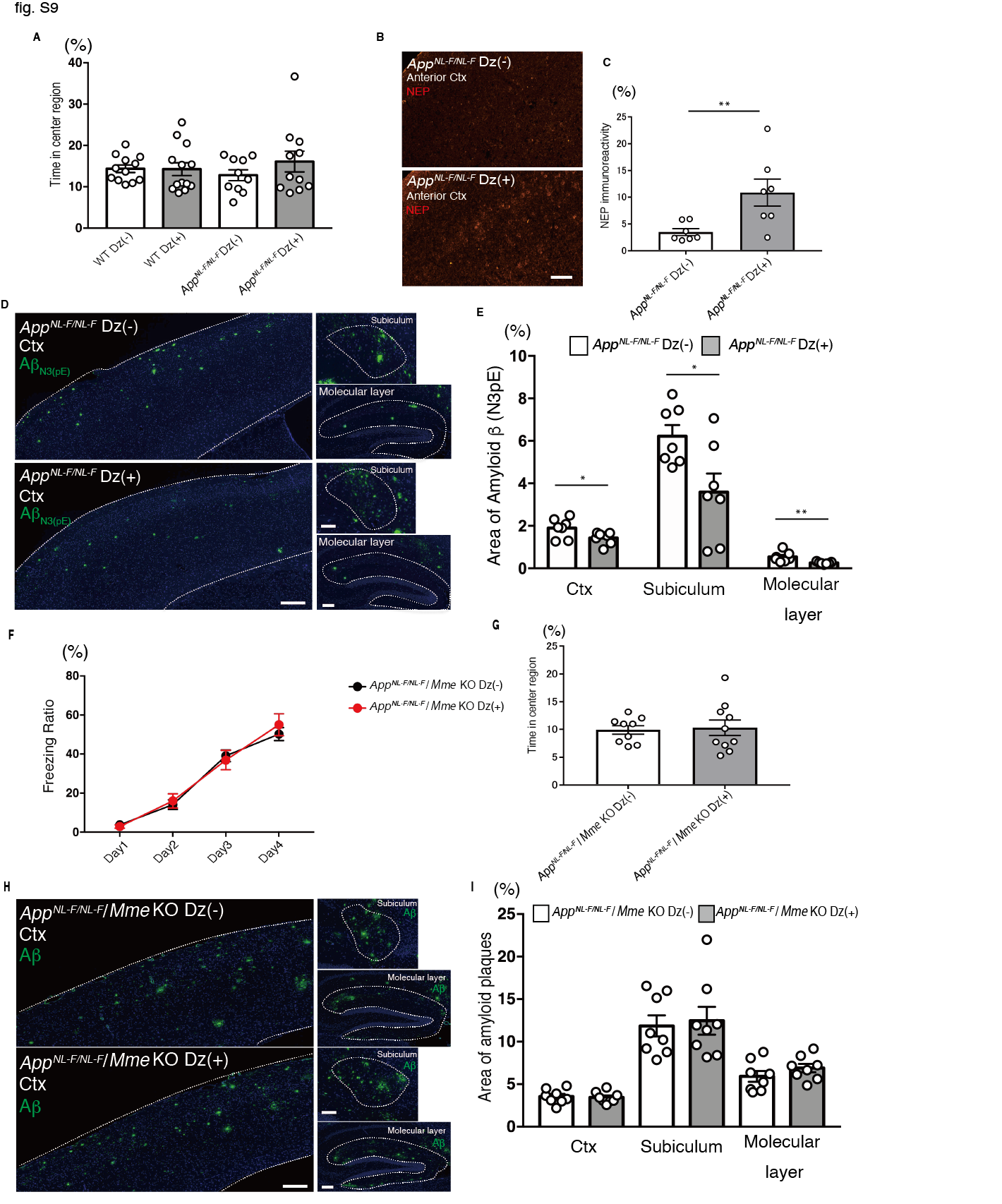


**Supplementary** **Figure S9. Effect of Dz in *App^NL-F^* and *App^NL-F^*/*Mme* KO mice.**

A. Statistical analysis of open field test to measure time in central region. 18-month-old WT and *App^NL-F^* mice were treated with or without Dz for 3 months (WT Dz (-): n = 12, WT Dz (+): n = 13, *App^NL-F^* Dz (-): n = 10, *App^NL-F^* Dz (+): n = 11). B and C. Immunostaining of NEP in cortices of 18-month-old *App^NL-F^* mice treated with or without Dz for 3 months (n = 7 for each group). Scale bar = 500 µm. D. Immunostaining of Aβ_N3(pE)_ (Green) in cortex, subiculum and molecular layer of 18-month-old *App^NL-F^* mice with or without Dz for 3 months. Scale bar in cortical image = 500 µm and in hippocampal image = 200µm. E. Statistical analysis of amyloid β_N3(pE)_ positive area in 18-month-old *App^NL-F^* treated with or without Dz for 3 months (n = 7 for each group). F. Freezing ratio of 15-month-old *App^NL-F^*/*Mme* KO mice treated with or without Dz for 3 months (*App^NL-F^*/*Mme* KO Dz (-): n = 9, *App^NL-F^*/*Mme* KO Dz (+): n = 10). G. Statistical analysis of open field test to measure time in central region of maze. 15-month-old *App^NL-F^*/*Mme* KO were treated with or without Dz for 3 months (*App^NL-F^*/*Mme* KO Dz (-): n = 9, *App^NL-F^*/*Mme* KO Dz (+): n = 10). H. Immunostaining of Aβ (Green) in cortex, subiculum and molecular layer of 15-month-old *App^NL-F^*/*Mme* KO mice with or without Dz for 3 months. Scale bar in cortical image = 500 µm and in hippocampal image = 200µm. I. Statistical analysis of amyloid plaque area in 15-month-old *App^NL-F^*/*Mme* KO treated with or without Dz for 3 months (n = 8 for each group). In (C)**,** the data represent the mean ±SEM. ***P*<0.01 (Mann-Whitney test). In (E**),** the data represent the mean ±SEM. **P*<0.05 (Student’s *t*-test).

**Supplementary Table S1. Antibodies information.**

The table shows the list of antibodies used in this study. Dilutions for Western blotting and immunostaining are described.

**Supplementary Table S2. The list of proteins identified in LC-MS/MS analysis of conditioned media from WT neuron.**

Candidate proteins regulating NEP activity were screened by the absence or presence only in the media of the SST- and TT232-treated WT neurons in the range of 10 to 30 kDa molecular weight, but not in the media of *Sst_1_/Sst_4_* dKO neurons (Supplementary Table S3). Red: Control samples, Blue: SST-treated samples, Green: TT232-treated samples. ENSA, NSG1 and NUCKS1 were highlighted in yellow.

 **Supplementary Table S3. The list of proteins identified in LC-MS/MS analysis of conditioned media from *Sst_1_/Sst_4_* dKO neuron.**

Candidate proteins regulating NEP activity were screened by the absence or presence only in the media of the SST- and TT232-treated WT neurons in the range of 10 to 30 kDa molecular weight (Supplementary Table S2), but not in the media of *Sst_1_/Sst_4_* dKO neurons. Red: Control samples, Blue: SST-treated samples, Green: TT232-treated samples. ENSA, NSG1 and NUCKS1 were highlighted in yellow.

**Supplementary Table S4. The list of primers for *in vitro* transcription.**

The table shows the list of primers for *in vitro* transcription. *In vitro* transcription was performed as previously described ^32^.

**Supplementary Table S5. Predicted Off-target regions in *Ensa* KO mice generated by CRISPR/Cas9.**

The table shows the list of off-target regions predicted by COSMID in *Ensa* KO mice ^33^.

**Supplementary Table S6. The list of primers to search for off-target in *Ensa* KO mice.**

The table shows the list of primers for off-target analysis in *Ensa* KO mice.

**Supplementary Table S7. Primers for Genotyping and RT-PCR.**

The table shows the list of primers for genotyping and RT-PCR.

**Supplementary Table S8. Amino acid sequences of ENSA cleaved by NEP.**

The table shows the list of amino acid sequences of ENSA cleaved by NEP.

**Supplementary Table S9. The list of human samples.**

The table shows information of human samples from left to right: Sample, Catalog/Sample ID, Lot, Gender, Age, Postmortem time, Region, Neuropathological diagnosis, Import source.

**Supplementary Table S10. Comparison of gene expression for each K_ATP_ channel component between AD patients and controls.**

Table showing the microarray and RNA sequencing (RNA-seq) statistical data for gene expression relating to each K_ATP_ channel component between AD patients and controls as derived from public cohort studies.

**Supplementary Table S11. Gene expression of *ABCC8* and *LCNJ11* with differentiating Braak stages.**

Table showing RNA-seq statistical data for *ABCC8* and *KCNJ11* gene expression with differentiating Braak stages as derived from public cohort studies.
